# Systemic Anticancer Therapy and Thromboembolic Outcomes in Hospitalized Patients With Cancer and COVID-19

**DOI:** 10.1001/jamaoncol.2023.2934

**Published:** 2023-08-17

**Authors:** Shuchi Gulati, Chih-Yuan Hsu, Surbhi Shah, Pankil K. Shah, Rebecca Zon, Susan Alsamarai, Joy Awosika, Ziad El-Bakouny, Babar Bashir, Alicia Beeghly, Stephanie Berg, Daniel de-la-Rosa-Martinez, Deborah B. Doroshow, Pamela C. Egan, Joshua Fein, Daniel B. Flora, Christopher R. Friese, Ariel Fromowitz, Elizabeth A. Griffiths, Clara Hwang, Chinmay Jani, Monika Joshi, Hina Khan, Elizabeth J. Klein, Natalie Knox Heater, Vadim S. Koshkin, Daniel H. Kwon, Chris Labaki, Tahir Latif, Rana R. McKay, Gayathri Nagaraj, Elizabeth S. Nakasone, Taylor Nonato, Hyma V. Polimera, Matthew Puc, Pedram Razavi, Erika Ruiz-Garcia, Renee Maria Saliby, Aditi Shastri, Sunny R. K. Singh, Vicky Tagalakis, Diana Vilar-Compte, Lisa B. Weissmann, Cy R. Wilkins, Trisha M. Wise-Draper, Michael T. Wotman, James J. Yoon, Sanjay Mishra, Petros Grivas, Yu Shyr, Jeremy L. Warner, Jean M. Connors, Dimpy P. Shah, Rachel P. Rosovsky

**Affiliations:** 1 University of California Davis Comprehensive Cancer Center, Sacramento; 2 University of Cincinnati Cancer Center, Cincinnati, Ohio; 3 Department of Biostatistics, Vanderbilt University Medical Center, Nashville, Tennessee; 4 Division of Hematology/Oncology, Department of Medicine, Mayo Clinic Arizona, Phoenix; 5 Mays Cancer Center at University of Texas Health San Antonio MD Anderson; 6 Dana-Farber Cancer Institute and Massachusetts General Brigham, Boston; 7 Hartford HealthCare Cancer Institute, Hartford, Connecticut; 8 Dana-Farber Cancer Institute, Boston, Massachusetts; 9 Sidney Kimmel Cancer Center at Thomas Jefferson University, Philadelphia, Pennsylvania; 10 Vanderbilt-Ingram Cancer Center, Vanderbilt University, Nashville, Tennessee; 11 Loyola University Medical Center, Chicago, Illinois; 12 Instituto Nacional de Cancerología, Mexico City, Mexico; 13 Tisch Cancer Institute at the Icahn School of Medicine at Mount Sinai, New York, New York; 14 Brown University and Lifespan Cancer Institute, Providence, Rhode Island; 15 St Elizabeth Healthcare, Edgewood, Kentucky; 16 University of Michigan Rogel Cancer Center, Ann Arbor; 17 Montefiore Medical Center, Albert Einstein College of Medicine, New York, New York; 18 Roswell Park Comprehensive Cancer Center, Buffalo, New York; 19 Henry Ford Cancer Institute, Henry Ford Hospital, Detroit, Michigan; 20 Mount Auburn Hospital, Boston, Massachusetts; 21 Penn State Cancer Institute, Hershey, Pennsylvania; 22 Brown University and Lifespan Cancer Institute, Providence, Rhode Island; 23 UCSF Helen Diller Family Comprehensive Cancer Center at the University of California San Francisco; 24 Moores Cancer Center, University of California San Diego; 25 Loma Linda University Cancer Center, Loma Linda, California; 26 Seattle Cancer Care Alliance, Fred Hutchinson Cancer Research Center, University of Washington, Seattle; 27 Virtua Health, Marlton, New Jersey; 28 University of Arkansas for Medical Sciences, Little Rock; 29 Division of Internal Medicine and Centre for Clinical Epidemiology of the Lady Davis Institute for Medical Research, Jewish General Hospital, Montreal, Quebec, Canada; 30 Memorial Sloan Kettering Cancer Center, New York, New York; 31 New York Presbyterian Hospital-Weill Cornell Medicine, New York, New York; 32 Lifespan Cancer Institute, Providence, Rhode Island; 33 Division of Hematology, Brigham and Women’s Hospital, Boston, Massachusetts; 34 Division of Hematology, Department of Medicine, Massachusetts General Hospital and Harvard Medical School, Boston

## Abstract

**Importance:**

Systematic data on the association between anticancer therapies and thromboembolic events (TEEs) in patients with COVID-19 are lacking.

**Objective:**

To assess the association between anticancer therapy exposure within 3 months prior to COVID-19 and TEEs following COVID-19 diagnosis in patients with cancer.

**Design, Setting, and Participants:**

This registry-based retrospective cohort study included patients who were hospitalized and had active cancer and laboratory-confirmed SARS-CoV-2 infection. Data were accrued from March 2020 to December 2021 and analyzed from December 2021 to October 2022.

**Exposure:**

Treatments of interest (TOIs) (endocrine therapy, vascular endothelial growth factor inhibitors/tyrosine kinase inhibitors [VEGFis/TKIs], immunomodulators [IMiDs], immune checkpoint inhibitors [ICIs], chemotherapy) vs reference (no systemic therapy) in 3 months prior to COVID-19.

**Main Outcomes and Measures:**

Main outcomes were (1) venous thromboembolism (VTE) and (2) arterial thromboembolism (ATE). Secondary outcome was severity of COVID-19 (rates of intensive care unit admission, mechanical ventilation, 30-day all-cause mortality following TEEs in TOI vs reference group) at 30-day follow-up.

**Results:**

Of 4988 hospitalized patients with cancer (median [IQR] age, 69 [59-78] years; 2608 [52%] male), 1869 had received 1 or more TOIs. Incidence of VTE was higher in all TOI groups: endocrine therapy, 7%; VEGFis/TKIs, 10%; IMiDs, 8%; ICIs, 12%; and chemotherapy, 10%, compared with patients not receiving systemic therapies (6%). In multivariable log-binomial regression analyses, relative risk of VTE (adjusted risk ratio [aRR], 1.33; 95% CI, 1.04-1.69) but not ATE (aRR, 0.81; 95% CI, 0.56-1.16) was significantly higher in those exposed to all TOIs pooled together vs those with no exposure. Among individual drugs, ICIs were significantly associated with VTE (aRR, 1.45; 95% CI, 1.01-2.07). Also noted were significant associations between VTE and active and progressing cancer (aRR, 1.43; 95% CI, 1.01-2.03), history of VTE (aRR, 3.10; 95% CI, 2.38-4.04), and high-risk site of cancer (aRR, 1.42; 95% CI, 1.14-1.75). Black patients had a higher risk of TEEs (aRR, 1.24; 95% CI, 1.03-1.50) than White patients. Patients with TEEs had high intensive care unit admission (46%) and mechanical ventilation (31%) rates. Relative risk of death in patients with TEEs was higher in those exposed to TOIs vs not (aRR, 1.12; 95% CI, 0.91-1.38) and was significantly associated with poor performance status (aRR, 1.77; 95% CI, 1.30-2.40) and active/progressing cancer (aRR, 1.55; 95% CI, 1.13-2.13).

**Conclusions and Relevance:**

In this cohort study, relative risk of developing VTE was high among patients receiving TOIs and varied by the type of therapy, underlying risk factors, and demographics, such as race and ethnicity. These findings highlight the need for close monitoring and perhaps personalized thromboprophylaxis to prevent morbidity and mortality associated with COVID-19–related thromboembolism in patients with cancer.

## Key Points

**Question:**

Is there an association between systemic anticancer therapy and the risk of thromboembolic events in hospitalized patients with cancer and COVID-19?

**Findings:**

In this cohort study of 4988 hospitalized patients with cancer, patients with active cancer and COVID-19 had a higher risk of developing venous thromboembolism when exposed to systemic anticancer therapies within 3 months prior to COVID-19 diagnosis compared with patients not receiving systemic therapy. Patients with COVID-19–related thromboembolic events who were receiving systemic therapies experienced worse outcomes compared with those not receiving such therapies.

**Meaning:**

Patients with cancer and COVID-19 need close monitoring for thromboembolism and consideration for risk-mitigating thromboprophylaxis, especially if recently exposed to systemic anticancer drugs.

## Teaser

This cohort study assesses the association between anticancer therapy exposure and thromboembolic events following COVID-19 diagnosis in patients with cancer.

## Introduction

As of October 2022, COVID-19 had resulted in more than 6 million deaths globally.^1^ Outcomes vary from mild illness to those requiring critical care. Even though a thromboinflammatory state in patients diagnosed with COVID-19 was described early in the pandemic,^2^ initial studies provided conflicting data.^3,4^ Subsequent large population-based studies, however, uniformly confirmed the association of COVID-19 and venous thromboembolism (VTE).^5,6,7^

Patients with cancer are a population of interest because of their higher predisposition to clot formation, with exposure to systemic anticancer therapy a well-described risk factor.^8,9,10,11,12,13,14^ Li et al^15^ previously published data on elevated thrombotic risk in hospitalized patients with cancer with COVID-19. In the current study, our objective was to evaluate the association between exposure to anticancer therapies within 90 days prior to COVID-19 diagnosis and thromboembolic events (TEEs) following COVID-19 in patients with cancer. We hypothesized that patients with cancer receiving certain high-risk systemic therapies would have a higher incidence of TEEs when compared with patients with cancer who were not receiving any systemic therapies. We leveraged data from a cohort of more than 12 000 patients in the COVID-19 and Cancer Consortium (CCC19) registry. We also aimed to identify other prognostic factors associated with TEEs in patients with cancer hospitalized for COVID-19, including sociodemographic and disease profiles. Additionally, we evaluated the severity of COVID-19 outcomes, including the 30-day case fatality rate in patients with COVID-19–related TEEs.

## Methods

### Study Cohort

**Figure 1.**
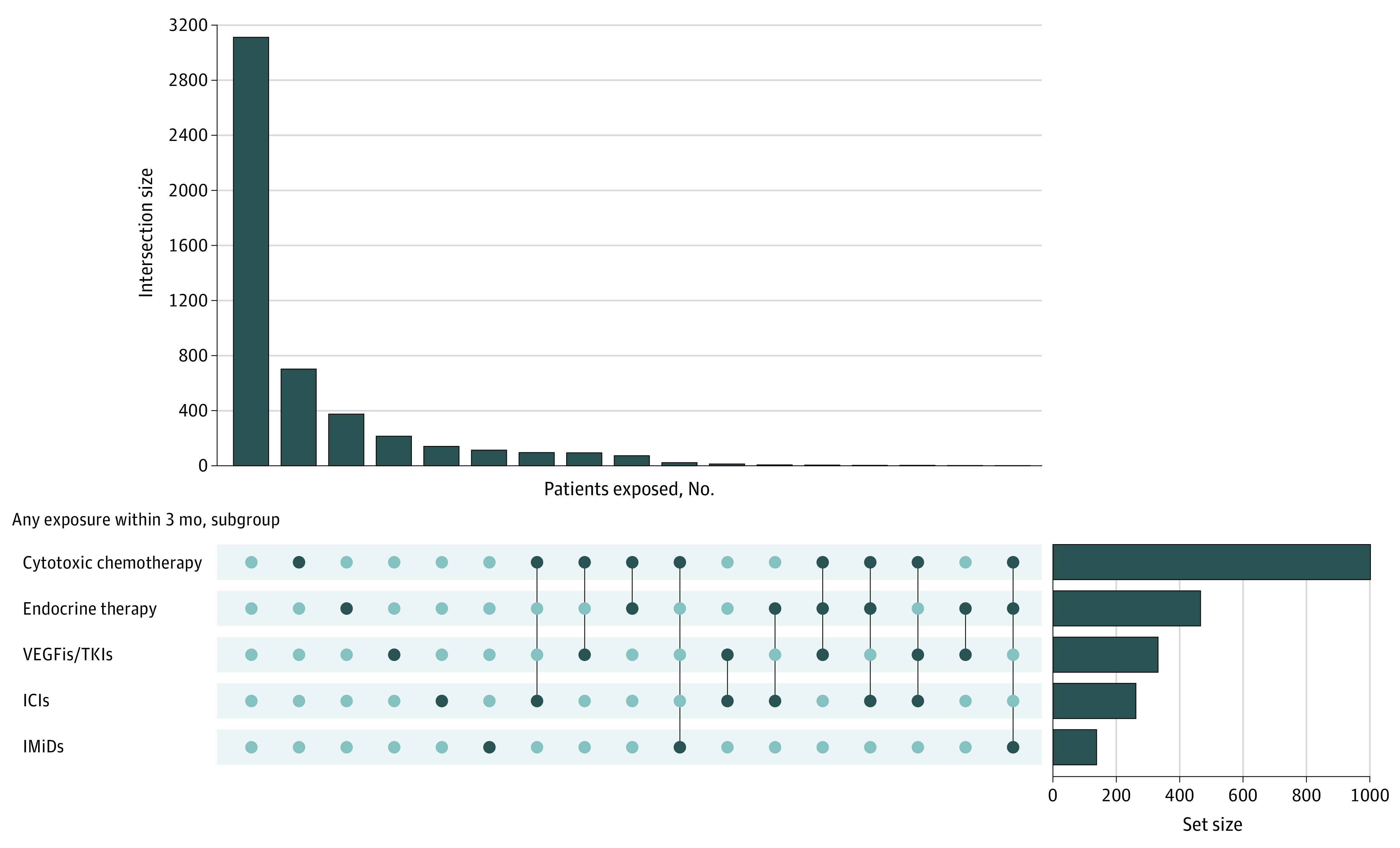
UpSet Plot of Treatment Exposures of Interest The largest subgroup (n = 3119) did not have any of the treatment exposures of interest within 3 months prior to COVID-19 diagnosis; the next 4 largest subgroups each consist of patients exposed to 1 of the exposure groups. Dual exposures, eg, cytotoxic chemotherapy plus ICI within 3 months, comprise the remainder of the common subgroups. No patient was simultaneously exposed to 4 or more of the 5 exposure groups. Across all patients, cytotoxic chemotherapy (n = 1002) was the most common exposure. ICIs indicates immune checkpoint inhibitors; IMiDs, immunomodulators; TKIs, tyrosine kinase inhibitors; VEGFis, vascular endothelial growth factor inhibitors.

The CCC19 maintains an international multi-institutional registry of patients with COVID-19 with a current or past invasive cancer diagnosis. Prospectively collected data from electronic health records at multiple institutions are housed in a REDCap database at Vanderbilt University. A list of participating institutions (n = 126) is provided in the eAppendix in Supplement 1. Follow-up data are collected at 30, 90, and 180 days after COVID-19 diagnosis and then annually.^16^ The CCC19 data dictionary is available in eTable 1 in Supplement 1.^16^ For our study, we used data on patients with cancer accrued from March 2020 to December 2021 who had laboratory-confirmed SARS-CoV-2 infection by polymerase chain reaction and/or serology testing, a minimum 30-day follow-up, and met quality standards (score ≤4).^16^ We assigned patients into 5 groups according to exposure to anticancer therapies (treatments of interest [TOIs]) in the 3 months before COVID-19: (1) endocrine therapy, (2) vascular endothelial growth factor inhibitors/tyrosine kinase inhibitors (VEGFis/TKIs), (3) immunomodulators (IMiDs), (4) immune checkpoint inhibitors (ICIs), and (5) chemotherapy. Patients in each group were not mutually exclusive (Figure 1). Outcomes were compared with a reference group of patients with cancer who had not received any systemic therapy in the 3 months before COVID-19 diagnosis. This study was exempt from institutional review board (IRB) review (VUMC IRB No. 200467) because it used only deidentified data; informed consent was waived for the same reason. Local IRB approval was left to site discretion. This study is registered on ClinicalTrials.gov (NCT04354701).

### Outcome Definitions and Statistical Analysis

The primary outcomes were incidence of (1) VTE and (2) arterial thromboembolism (ATE) following COVID-19 in hospitalized patients with cancer within 30 and 90 days. Data on ATE/VTE were collected through binary checkboxes in a standard survey sent out to all sites. We compared the incidence of (1) VTE and (2) ATE in patients who had received TOIs within 3 months prior to a COVID-19 diagnosis vs those who had not received any systemic therapy (reference group). Secondary outcomes were rates of intensive care unit admission, mechanical ventilation after COVID-19, and 30-day all-cause mortality following VTE/ATE in the 2 groups (TOIs vs reference).

Before regression analyses, we applied multiple imputations with 10 imputations for all missing values of covariates using additive regression, bootstrapping, and predictive mean matching. The imputation method took all aspects of uncertainty into account by using bootstrap to approximate the process of drawing predicted values from a full bayesian predictive distribution.^17^ We conducted multivariable log-binomial regression analyses and computed adjusted risk ratio (aRR) for TEEs for TOI vs reference arm. To identify other prognostic factors, we performed multivariable regression analyses for (1) VTE, (2) ATE, and (3) combined VTE/ATE in each of the imputed data sets. For VTE, we adjusted for age, sex, race and ethnicity, obesity (body mass index [BMI, calculated as weight in kilograms divided by height in meters squared] ≥30), Eastern Cooperative Oncology Group (ECOG) status, cancer status (active, stable/responding, active/progressing), metastatic cancer (yes vs no), prior history of VTE, cancer site by VTE risk adapted from the Khorana score^18^ (stomach, pancreas, lung, lymphoma, gynecologic, bladder, kidney/pelvis, and germ cell/testicular cancers were included as high-risk cancers) (eTable 2 in Supplement 1), and baseline anticoagulation, aspirin, and antiplatelet use. The categories for race and ethnicity were Asian, Asian American or Pacific Islander, non-Hispanic Black (hereafter, *Black*), Hispanic, and non-Hispanic White (hereafter, *White*), and other; options were predefined in the survey that was sent out to each site. For ATE, additional covariates included a history of diabetes, atrial fibrillation, and past ATE (coronary artery disease, cerebrovascular disorders, peripheral arterial disease). Variables were selected based on prior studies.^19,20,21^ Diabetes was not included in the VTE model because the increased risk of VTE associated with diabetes has been attributed to other concomitant comorbidities, rather than to diabetes itself.^22,23^ Recent surgery was added as a covariate in a scenario analysis (eTables 8A and 8B in Supplement 1) and not the main model because surgical data were not reliable in this registry-based study (eTable 3 in Supplement 1).

Further, for secondary outcomes, we performed regression analysis aiming to identify 30-day mortality risk after TEE, in patients exposed to TOIs vs the reference group. We also compared outcomes at different cut points following COVID-19 diagnosis (ever, 30 days, and 90 days). Multicollinearity diagnostic testing was performed for all models, with variance inflation factor (VIF) of 3 or higher to suggest potential existence of multicollinearity.

Separate exploratory multivariable analyses were performed where (1) recent surgery was included as a covariate, and (2) patients receiving multiple therapies (n = 319 [14%]) were excluded. Additionally, we explored whether an interaction existed between TOIs and severity of COVID-19 (by symptoms: moderate vs mild and severe vs mild) for ATE/VTE risk by performing a multivariable analysis that included interaction terms between these 2 variables.

Analyses were performed using R, version 4.0.3 (R Foundation for Statistical Computing), and R packages Hmisc4.5-0, rms6.2-0, and forestplot1.10.1.

## Results

### Patient Characteristics

From March 2020 to December 2021, 12 257 patients with cancer and laboratory-confirmed SARS-CoV-2 infection were enrolled in CCC19. After excluding nonhospitalized cases (n = 5721), unknown/missing exposure of interest (n = 997), quality scores greater than 4 (n = 209), unknown/missing primary outcomes ATE/VTE (n = 175), and patients with a systemic treatment other than TOIs (n = 167), 4988 patients were included in the analyses (eFigure 1 in Supplement 1). Median (IQR) age was 69 (59-78) years, and 48% (n = 2376) were female. The cohort included 21% (n = 1032) Black patients, 16% (n = 803) Hispanic patients, and 51% (n = 2533) White patients. Current or former smoking was reported in 49% (n = 2454), and 36% (n = 1796) of patients were reported to be obese (BMI ≥30). A total of 44% (n = 2179) had active cancer, and 31% (n = 1541) had metastatic cancer. Risk stratification adapted from the Khorana score^18^ revealed 34% (n = 1675) of patients with high risk for VTE (eTable 2 in Supplement 1). Prehospitalization anticoagulant use was reported in 19% (n = 963), and aspirin use in 30% (n = 1489) (Table 1; eTable 4A in Supplement 1).

**Table 1.** Baseline Characteristics and Laboratory Parameters of Hospitalized Patients With Cancer and COVID-19 Exposed to Systemic Therapies of Interest in the 3 Months Preceding COVID-19 Diagnosis

Characteristic	No. (%)						
	Total	Endocrine therapy	VEGFis/TKIs	IMiDs	ICIs	Cytotoxic chemotherapy	No exposure to systemic therapy
Total No.^a^^,^^b^	4988	466	332	138	262	1002	3119
Median (IQR) age, y	69 (59-78)	72 (61-80)	65 (54-74)	67 (59-75)	68 (58-76)	63 (53-72)	71 (61-79)
Sex							
Female	2376 (48)	249 (53)	154 (46)	54 (39)	114 (44)	531(53)	1440 (46)
Male	2608 (52)	217 (47)	178 (54)	84 (61)	148 (56)	470 (47)	1676 (54)
Missing	4 (<1)	0	0	0	0	1 (<1)	3 (<1)
Race and ethnicity							
Asian American or Pacific Islander	185 (4)	17 (4)	16 (5)	4 (3)	12 (5)	33 (3)	116 (4)
Black	1032 (21)	110 (24)	52 (16)	45 (33)	36 (14)	204 (20)	644 (21)
Hispanic	803 (16)	94 (20)	77 (23)	24 (17)	43 (16)	220 (22)	420 (13)
White	2533 (51)	210 (45)	165 (50)	52 (38)	150 (57)	465 (46)	1655 (53)
Other^c^	363 (7)	29 (6)	19 (6)	8 (6)	16 (6)	68 (7)	240 (8)
Missing	72 (1)	6 (1)	3 (1)	5 (4)	5 (2)	12 (1)	44 (1)
BMI ≥30							
Yes	1796 (36)	191 (41)	108 (33)	50 (36)	67 (26)	316 (32)	1163 (37)
No	3168 (64)	274 (59)	218 (66)	88 (64)	195 (74)	682 (68)	1942 (62)
Missing	24 (<1)	1 (<1)	6 (1)	0	0	4 (<1)	14 (1)
ECOG status							
0	1182 (24)	129 (28)	83 (25)	35 (25)	47 (18)	210 (21)	745 (24)
1	1350 (27)	136 (29)	126 (38)	49 (36)	124 (47)	402 (40)	645 (21)
≥2	1045 (21)	126 (38)	68 (20)	27 (20)	68 (26)	248 (25)	590 (19)
Unknown	1406 (28)	81 (17)	54 (16)	27 (20)	22 (8)	141 (14)	1136 (36)
Missing	5 (<1)	0	1 (<1)	0	1 (<1)	1 (<1)	3 (<1)
Smoking status							
Never	2351 (47)	221 (47)	184 (55)	78 (57)	89 (34)	492 (49)	1431 (46)
Current or former	2454 (48)	225 (48)	131 (39)	51 (37)	476 (48)	476 (48)	1578 (51)
Missing	183 (4)	20 (4)	17 (5)	9 (7)	4 (2)	34 (3)	110 (4)
Cancer status							
Remission/NED	2296 (46)	140 (30)	34 (10)	22 (16)	9 (3)	89 (9)	2021 (67)
Active, stable/responding	1341 (27)	200 (43)	174 (52)	87 (63)	134 (51)	423 (15)	478 (15)
Active, progressing	838 (17)	94 (27)	253 (26)	15 (11)	85 (32)	341 (34)	325 (10)
Unknown	510 (10)	32 (7)	33 (10)	14 (10)	34 (13)	148 (15)	293 (9)
Missing	3 (<1)	0	0	0	0	1 (<1)	2 (<1)
Metastatic cancer							
No	3172 (64)	218 (42)	86 (26)	30 (22)	55 (21)	338 (34)	2511 (81)
Yes	1541 (31)	225 (48)	229 (69)	86 (62)	191 (73)	603 (60)	460 (15)
Missing	275 (6)	23 (5)	17 (5)	22 (16)	16 (6)	61 (6)	148 (5)
Cancer risk (Khorana score)^d^							
High risk for VTE	1675 (34)	29 (6)	103 (31)	11 (8)	173 (66)	427 (43)	1060 (34)
Low risk for VTE	3313 (66)	437 (94)	229 (69)	127 (92)	89 (34)	575 (57)	2059 (66)
Prior VTE							
No	4426 (89)	415 (89)	282 (85)	118 (86)	232 (89)	851 (85)	2813 (90)
Yes	537 (11)	50 (11)	43 (13)	20 (14)	30 (11)	146 (15)	293 (9)
Missing	25 (1)	1 (<1)	7 (2)	0	0	5 (<1)	13 (1)
Preadmission anticoagulation							
None	3794 (76)	339 (73)	240 (72)	103 (75)	193 (74)	721 (72)	2426 (78)
Prophylactic dosing	445 (9)	42 (9)	32 (10)	15 (11)	31 (12)	118 (12)	252 (8)
Therapeutic dosing	518 (10)	59 (13)	44 (13)	15 (11)	29 (11)	115 (11)	301 (11)
Unknown	231 (5)	26 (6)	16 (5)	5 (4)	9 (3)	48 (5)	140 (4)
Preadmission aspirin use							
None	3418 (69)	304 (65)	264 (80)	62 (45)	196 (75)	806 (80)	2033 (65)
Low dose (<200 mg/d)	1397 (28)	148 (32)	59 (18)2	65 (47)	60 (23)	173 (17)	967 (31)
Full dose	92 (2)	7 (2)	2 (1)	9 (7)	2 (1)	9 (1)	65 (2)
Unknown	81 (2)	7 (2)	7 (2)	2 (1)	4 (2)	14 (1)	54 (2)
Preadmission antiplatelet agent (other than aspirin)							
None	4655 (93)	433 (93)	314 (95)	133 (96)	254 (97)	965 (96)	2871 (92)
Yes	284 (6)	30 (6)	12 (4)	5 (4)	7 (3)	28 (3)	215 (6)
Missing	49 (1)	3 (1)	6 (1)	0	1 (<1)	9 (1)	33 (1)
D-Dimer							
Normal	409 (8)	37 (8)	29 (9)	16 (12)	22 (8)	62 (6)	263 (8)
Abnormal	2242 (45)	247 (53)	139 (42)	55 (40)	100 (38)	433 (43)	1416 (45)
Unknown	399 (8)	39 (8)	39 (12)	17 (12)	23 (9)	117 (12)	204 (7)
Missing	1938 (39)	143 (31)	125 (38)	50 (36)	117 (45)	390 (39)	1236 (40)
WBC count							
Normal	2685 (54)	284 (61)	161 (48)	67 (49)	123 (47)	416 (42)	1794 (58)
High	796 (16)	55 (12)	63 (19)	4 (3)	47 (18)	151 (15)	527 (17)
Low	935 (19)	78 (17)	70 (21)	55 (40)	50 (19)	335 (33)	436 (14)
Missing	572 (11)	49 (11)	38 (11)	12 (9)	42 (16)	100 (10)	362 (12)
Platelet count							
Normal	2831 (57)	298 (64)	165 (50)	60 (43)	126 (48)	461 (46)	1889 (61)
High	208 (4)	12 (3)	10 (3)	4 (3)	16 (6)	48 (5)	135 (4)
Low	1323 (27)	98 (21)	114 (34)	61 (44)	75 (29)	376 (38)	707 (23)
Missing	626 (12)	58 (12)	43 (13)	13 (9)	45 (17)	117 (12)	388 (12)
IL-6							
Normal	93 (2)	8 (2)	4 (1)	1 (1)	7 (3)	16 (2)	61 (2)
Abnormal	559 (11)	63 (14)	52 (16)	23 (17)	31 (12)	148 (15)	304 (10)
Unknown	701 (14)	67 (14)	56 (17)	26 (19)	51 (19)	168 (17)	399 (13)
Missing	3635 (73)	328 (70)	220 (66)	88 (64)	173 (66)	670 (67)	2355 (76)

Abbreviations: BMI, body mass index, calculated as weight in kilograms divided by height in meters squared; ECOG, Eastern Cooperative Oncology Group; ICIs, immune checkpoint inhibitors; IL-6, interleukin 6; IMiDs, immunomodulators; NED, no evidence of disease; VTE, venous thromboembolism; WBC, white blood cell.

^a^ Numbers do not add up to 100% because treatments were not mutually exclusive. Some patients could have received 2 drugs at the same time.

^b^ “No exposure to systemic therapy” group included patients who had not received any cancer treatment in the 3 months prior to COVID-19.

^c^ Patient reported “other” on the survey if they did not identify with any of the categories available.

^d^ Adapted from Khorana score and literature^24^: high-risk group included stomach, esophagus, lung, pancreatic, gynecological, germ-cell tumors, kidney, bladder, and lymphomas. Low-risk group included all other cancers, including “other solid” and “other hematologic.”

Of the TOIs, 466 (9%) patients received endocrine therapy, 332 (7%) VEGFis/TKIs, 138 (3%) IMiDs, 262 (5%) ICIs, and 1002 (20%) chemotherapy in the 3 months preceding COVID-19. In addition, 3119 (62%) patients who had not received any systemic therapy served as the reference group (eTable 4B in Supplement 1). Patients receiving TOIs were not mutually exclusive (Figure 1), with dual exposures, eg, cytotoxic chemotherapy plus ICI, comprising common subgroups. No patient was simultaneously exposed to 4 or more TOIs. Missing rates of variables (Table 1; eTable 4A in Supplement 1) were less than 6% in the regression analysis models, while for 5 laboratory variables used for descriptive statistics, they ranged from 11% to 79%. A comparison of clinical characteristics between patients receiving systemic therapies compared with those not receiving these therapies is shown in eTable 5 in Supplement 1.

### VTE and ATE Rates in Patients With Cancer and COVID-19

Incidence of overall TEEs among the 4988 hospitalized patients was 11% (362 [7%; 95% CI, 7%-8%] patients with VTE and 198 [4%; 95% CI, 3%-4%] with ATE). In the reference group, incidence of TEEs was 11% (VTE: 6% [95% CI, 5%-7%] and ATE: 5% [95% CI, 4%-5%]). Individually, the incidence of VTE was uniformly higher in all TOI groups: endocrine therapy, 7% (95% CI, 5%-9%); VEGFis/TKIs, 10% (95% CI, 7%-13%); IMiDs, 8% (95% CI, 4%-13%); ICIs, 12% (95% CI, 8%-16%); and chemotherapy, 10% (95% CI, 9%-12%). In contrast, ATE incidence was the same or lower: endocrine therapy, 5% (95% CI, 3%-7%); VEGFis/TKIs, 2% (95% CI, 5%-7%); IMiDs, 4% (95% CI, 1%-7%); ICIs, 2% (95% CI, 0%-3%); and chemotherapy, 3% (95% CI, 2%-4%) (Table 2).

**Table 2.** Incidence of VTE and ATE in Patients Exposed to 5 Anticancer Therapies and Reference Group Unexposed to Any Systemic Therapy

Agent/agent group	Incidence of VTE (95% CI)	Incidence of ATE (95% CI)^a^
No.	4988	4988
Total population	362/4988 = 0.07 (0.07-0.08)	198/4988 = 0.04 (0.03-0.04)
Endocrine therapy	32/466 = 0.07 (0.05-0.09)	22/466 = 0.05 (0.03-0.07)
VEGFis/TKIs	33/332 = 0.10 (0.07-0.13)	7/332 = 0.02 (0.01-0.04)
IMiDs	11/138 = 0.08 (0.04-0.13)	5/138 = 0.04 (0.01-0.07)
ICIs	31/262 = 0.12 (0.08-0.16)	4/262 = 0.02 (0.00-0.03)
Cytotoxic chemotherapy	105/1002 = 0.10 (0.09-0.12)	30/1002 = 0.03 (0.02-0.04)
Unexposed to any systemic treatment^b^	190/3119 = 0.06 (0.05-0.07)	141/3119 = 0.05 (0.04-0.05)

Abbreviations: ATE, arterial thromboembolism; ICIs, immune checkpoint inhibitors; IMiDs, immunomodulators; VEGFis/TKIs, vascular endothelial growth factor inhibitors/tyrosine kinase inhibitors; VTE, venous thromboembolism.

^a^ The total sum of ATE events exceeds 198 because the 5 treatment cohorts were not mutually exclusive (eFigure 2 in Supplement 1).

^b^ Reference group.

### Relative Risk for VTE in Patients Exposed to TOIs vs Not

**Figure 2.**
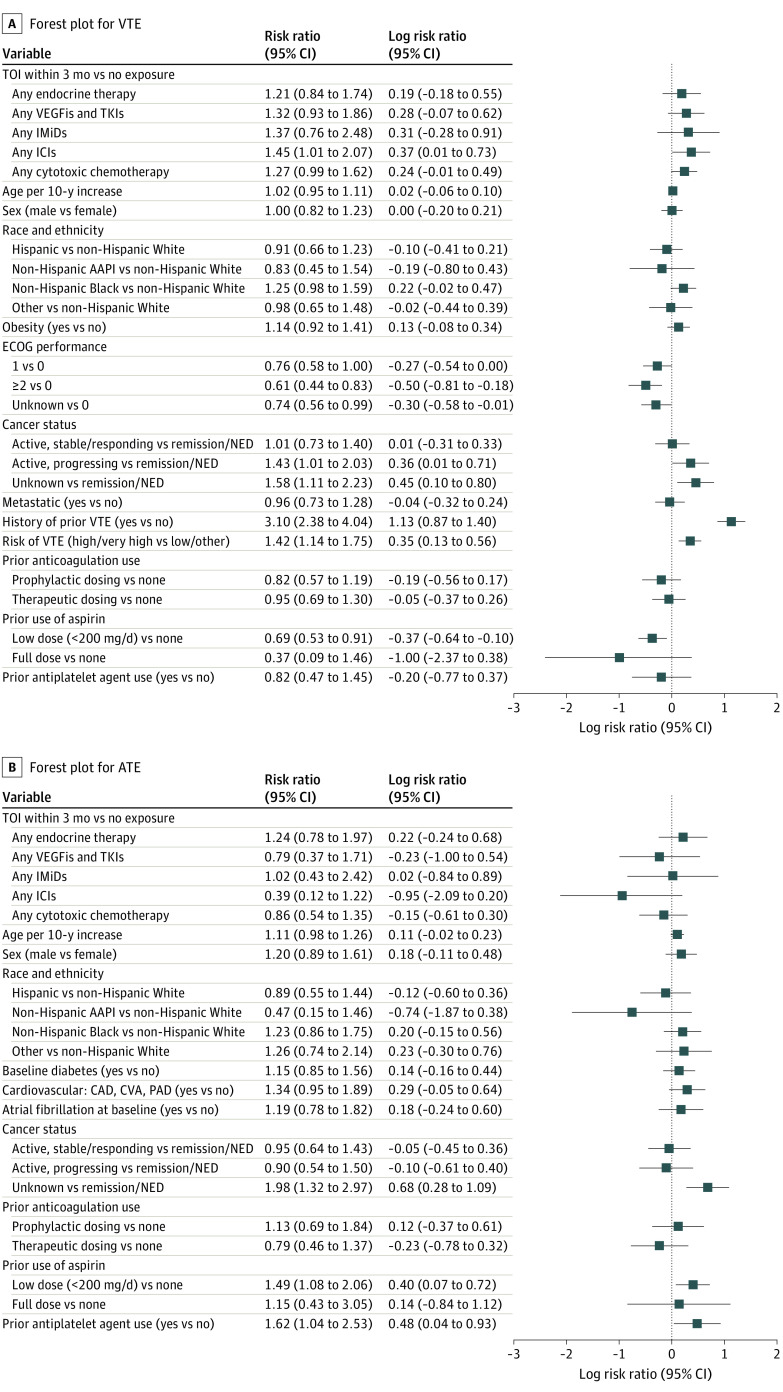
Forest Plots for Venous Thromboembolism (VTE) and Arterial Thromboembolism (ATE) Forest plots for the estimates of log risk ratios with the 95% CIs for exposure to the treatments of interest (TOI) within 3 months vs those not receiving systemic therapies in these 3 months. AAPI indicates Asian American and Pacific Islander; CAD, coronary artery disease; CVA, cerebrovascular accident; ECOG, Eastern Cooperative Oncology Group; ICIs, immune checkpoint inhibitors; IMiDs, immunomodulators; NED, no evidence of disease; PAD, peripheral arterial disease; TKIs, tyrosine kinase inhibitors; VEGFis, vascular endothelial growth factor inhibitors.

In the log-binomial regression model for VTE, exposure to TOIs pooled together was associated with significantly higher risk of VTE (aRR, 1.33; 95% CI, 1.04-1.69) (eFigure 4A in Supplement 1). When evaluated individually, exposure to ICIs was significantly associated with VTE (aRR, 1.45; 95% CI, 1.01-2.07). Although other TOIs were associated with higher risk of VTE, these associations were not significant (Table 3 and Figure 2A). Other variables that were associated with significantly higher risk of VTE included active/progressing cancer (aRR, 1.43; 95% CI, 1.01-2.03), history of VTE (aRR, 3.10; 95% CI, 2.38-4.04), and cancers at high risk of VTE (Khorana score) (aRR, 1.42; 95% CI, 1.14-1.75).

**Table 3.** Results of Regression Analysis for Primary Outcome of ATE and VTE in Patients Treated With Each of the 5 Anticancer Therapies in the Last 3 Months vs Not Receiving Any Systemic Treatments Within 3 Months, With Adjustment for Covariates

Treatment	aRR for VTE within 3 mo of treatment (95% CI)^a^	aRR for ATE within 3 mo of treatment (95% CI)^b^
All TOIs pooled together	1.33 (1.04-1.69)	0.81 (0.56-1.16)
Endocrine therapy	1.21 (0.84-1.74)	1.24 (0.78-1.97)
VEGFi/TKIs	1.32 (0.93-1.86)	0.79 (0.37-1.71)
IMiDs	1.37 (0.76-2.48)	1.02 (0.43-2.42)
ICIs	1.45 (1.01-2.07)	0.39 (0.12-1.22)
Cytotoxic chemotherapy	1.27 (0.99-1.62)	0.86 (0.54-1.35)

Abbreviations: aRR, adjusted risk ratio; ATE, arterial thromboembolism; ICIs, immune checkpoint inhibitors; IMiDs, immunomodulators; TOIs, treatments of interest; VEGFis/TKIs, vascular endothelial growth factor inhibitors/tyrosine kinase inhibitors; VTE, venous thromboembolism.

^a^ Adjusted for age, sex, race and ethnicity, obesity, Eastern Cooperative Oncology Group performance status, cancer status, metastatic cancer status, prior VTE, cancer type according to VTE risk, prior anticoagulation use, prior use of aspirin, prior antiplatelet agent use.

^b^ Adjusted for age, sex, race and ethnicity, baseline diabetes, baseline history of atrial fibrillation, prior history of cardiovascular comorbidities (coronary artery disease, cerebrovascular accident, peripheral arterial diseases), cancer status at the time of COVID-19 diagnosis, and prior use of anticoagulation, aspirin, and baseline use of other antiplatelet agents.

### Relative Risk for ATE in Patients Exposed to TOIs vs Not

In the log-binomial regression model for ATE, relative risk was not significantly different between patients exposed to TOIs in the preceding 3 months vs reference (aRR, 0.81; 95% CI, 0.56-1.16) (eFigure 4B in Supplement 1). There was no association between ATE and individual drugs either (endocrine therapy: aRR, 1.24; 95% CI, 0.78-1.97; VEGFis/TKIs: aRR, 0.79; 95% CI, 0.37-1.71; IMIDs: aRR, 1.02; 95% CI, 0.43-2.42; ICIs: aRR, 0.39; 95% CI, 0.12-1.22; chemotherapy: aRR, 0.86; 95% CI, 0.54-1.35) (Table 3 and Figure 2B). While baseline low-dose aspirin use (<200 mg/d; aRR, 1.49; 95% CI, 1.08-2.06) and antiplatelet use (aRR, 1.62; 95% CI, 1.04- 2.53) were associated with significantly higher risk of ATE, full-dose aspirin use (325 mg/d) was not (aRR, 1.15; 95% CI, 0.43-3.05).

### Relative Risk for TEEs in Patients Exposed to TOIs vs Not

Relative risk of TEE was higher (but not statistically significant) in patients treated with TOIs (aRR, 1.10; 95% CI, 0.91-1.33). Other significantly associated variables with TEE included race (aRR, 1.24; 95% CI, 1.03-1.50, for Black patients compared with White patients), history of VTE (aRR, 2.33; 95% CI, 1.88-2.89), and high-risk cancer site (aRR, 1.20; 95% CI, 1.02-1.42) (eTable 6, eFigure 2 in Supplement 1).

All variables included in the models for relative risk for VTE, ATE, and TEEs in patients exposed to TOIs vs not had a VIF of 3 or less (eg, cancer status [active/progressing cancer]: VIF, 2.39), indicating the absence of multicollinearity.

### Secondary Outcomes of Interest

In patients who developed TEEs, 30-day mortality was 25% (95% CI, 21%-29%), the rate of admission to the intensive care unit was 46% (95% CI, 42%-51%), and the rate of mechanical ventilation was 31% (95% CI, 27%-35%) (eTable 7A in Supplement 1). Importantly, the incidence rate of death as a clinical outcome following ATE/VTE was higher in the TOI group vs reference (46% [95% CI, 39%-52%] vs 38% [95% CI, 32%-44%]) (eTable 7B, eFigure 3 in Supplement 1). Relative risk of death in those exposed to systemic therapies was high (aRR, 1.12; 95% CI, 0.91-1.38), although not statistically significant. However, patients receiving TOIs with progressing cancer (aRR, 1.55; 95% CI, 1.13-2.13) and those with poor performance status (aRR, 1.44; 95% CI, 1.05-1.97 for ECOG 1; and aRR, 1.77; 95% CI, 1.30-2.40 for ECOG ≥2) had a significantly higher risk of death.

### Scenario Analyses

In the first scenario, we included recent surgery as a covariate. This was not included in the main model due to a lack of reliable data on the type of surgery in this registry. We did not see a significant association between recent surgery and risk of VTE (aRR, 0.99; 95% CI, 0.62-1.57) or ATE (aRR, 0.90; 95% CI, 0.45-1.79) (eTables 8A and 8B in Supplement 1). In the second scenario, we excluded patients (14%; n = 319) who were receiving more than 1 systemic therapy at the time data were collected, and none of the associations were significant, including for the ICI group. Other significantly associated risk factors, however, remained the same (eTable 9A and 9B in Supplement 1). In an additional analysis, we did not find an interaction between the initial severity of COVID-19 and systemic therapies for the risk of ATE/VTE in the regression models (eTables 10A and 10B in Supplement 1). Lastly, we performed additional regression analysis at 30-day and 90-day follow-up for both VTE and ATE, which revealed similar outcomes (eTables 11A and 11B in Supplement 1).

## Discussion

We report the association between systemic TOIs (endocrine therapy, VEGFis/TKIs, IMiDs, ICIs, and chemotherapy) and TEEs in hospitalized patients with cancer and COVID-19. We found that exposure to TOIs pooled together within the 3 months prior to COVID-19 diagnosis was associated with a higher risk of VTE but not ATE than those who did not receive any recent systemic therapy. Importantly, among individual TOIs, exposure to ICIs in the previous 3 months was significantly associated with VTE. Further, as has been demonstrated in previous studies,^15,25^ we report a high overall 30-day mortality of approximately 25% in patients with cancer who developed TEEs. Importantly, the relative risk of death in patients who developed TEEs and were exposed to systemic therapies was high compared with those not receiving any recent systemic treatment.

In addition to our main findings, an association between TEEs and an individual’s race and ethnicity was identified in this study. Black patients had a significantly higher risk of developing TEEs compared with White patients. The ongoing association between adverse COVID-19–related outcomes based on an individual’s race or ethnicity, accounting for measurable confounders, has been a consistent theme throughout the pandemic.^26,27,28^ Addressing modifiable factors that can account for these findings is an ongoing unmet need and should be one of the highest priorities. Of note, our study did not measure ancestry, which has been shown in prior research to be associated with a generally increased risk of TEEs^29^; the interaction of ancestry and TEEs in patients with COVID-19 should be the subject of future study.

Our study also provides updated results from the CCC19 registry, describing the overall burden of TEEs in patients diagnosed with cancer and COVID-19. The incidence of VTE and ATE described is consistent with our prior report.^15^ We provide additional follow-up data, with the 3-month ATE and VTE incidence of 4% and 7%, respectively, demonstrating that most COVID-19–related TEEs occur within the first 3 months of COVID-19. Although not designed as a validation study, our multivariable model used components from our previously published CoVID-TE risk prediction model^15^ and supported the significance of VTE history and high-risk cancer subtype as adapted from the Khorana score^18^ as risk factors for the development of VTE.

Additionally, we found a significant association between the risk of ATE and prior usage of low-dose aspirin and antiplatelet agents, which has not been examined before, to our knowledge. One possible explanation is a higher underlying risk of arterial clots in patients receiving these drugs. From this registry-based data set, complete information on whether these drugs were continued during hospitalization is not available; however, it appears that prior use could not prevent clot formation in this high-risk patient population. To draw effective conclusions, a clinical trial may be warranted, especially using a higher dose of aspirin while balancing the risk of bleeding.

Patients with cancer have a higher baseline risk of VTE, which is further influenced by stage, type of cancer, and systemic anticancer therapies. While COVID-19 has been shown to enhance the risk of TEEs in patients with cancer, the contribution of systemic therapies has not been reported previously to our knowledge. Additionally, drugs are often combined in the metastatic setting and could further enhance the risk of TEEs, especially if a patient is infected with SARS-CoV-2. When we excluded patients who were receiving more than 1 systemic therapy, individual drugs were still seen to be associated with a higher risk of VTE; however, these associations were not significant, likely due to a smaller number of patients in each group.

Our study benefits from the large sample size from the largest crowdsourced cancer and COVID-19 registry, ie, CCC19, designed to identify risks and trends in patients with cancer during the pandemic. While the use of outpatient thromboprophylaxis in patients with all cancer types is currently advocated for patients receiving systemic therapy with a Khorana risk score of 2 or higher,^30,31,32^ there is lack of clinical implementation of these recommendations. In a survey, more than 50% of surveyed oncologists were unfamiliar with the Khorana score or current VTE guidelines.^33,34^ Our findings allow us to understand the natural history of TEEs in patients with cancer and COVID-19 and stress the importance of education and awareness to continue anticoagulant use in these high-risk situations.

### Limitations

Limitations of the study include reporting bias and the lack of precision on the exact timing of events and outcome intervals, as well as the inherent potential for bias and confounding associated with observational studies. The lack of granularity for some variables, such as timing of cancer diagnosis, use of systemic therapy for localized vs metastatic cancer, intensity of smoking, and missing data, presented a challenge. To circumvent some—for example, recent surgery—we performed scenario analysis including it as a covariate, where no association was noted with VTE. The database has spanned a timeline both before and after vaccine approval; however, we are unable to confirm the association of vaccines with TEEs, as only 3% of patients in the data set had received at least 1 dose of the COVID-19 vaccine. Despite these limitations, CCC19 is among the largest cohorts of patients with cancer and COVID-19 currently available for investigating outcomes and contains important covariates, eg, cancer status and ECOG performance status, which are not easily available in electronic medical record databases.

## Conclusions

This cohort study confirms prior findings for the increased propensity for individuals with cancer to experience TEEs following a diagnosis of COVID-19, and we add systematic and detailed information on the association with specific anticancer therapies, which has not been presented before. Specific recommendations regarding the benefit and risk of anticoagulants for the treatment or prevention of VTE in patients with cancer and COVID-19 are not available.^35^ Using novel data from the CCC19 registry, we conclude that patients with cancer and COVID-19 who require hospitalization and have recently received TOIs are at a relatively high risk of VTEs; those patients who experience a TEE are at very high risk of death (approximately 20%-25%). Although not designed to prove causality between systemic therapy exposure and TEEs, our study highlights a potential association, and further investigation with prospective studies and randomized clinical trials is recommended to address risk-mitigation strategies.

## Notes

Supplement 1. **eTable 1.** Data Dictionary used for this analysis **eTable 2.** Cancer site classification for the study **eTable 3.** Patients in each treatment arm who had undergone recent surgery **eTable 4A.** Variable information **eTable 4B.** List of drugs included in each exposure category **eTable 5.** Patients with VTE and ATE: Head-to-head comparison of clinical features in those who were on systemic therapies vs. in those not on these therapies **eTable 6.** Results of regression analysis for combined VTE/ATE outcome, each anti-cancer therapy exposure within 3 months vs not receiving any systemic treatments within 3 months, with adjustment for covariates **eTable 7A.** Secondary outcomes in patients in each group (within 3 months) with no ATE/VTE **eTable 7B.** Results of regression analysis for death outcome after VTE/ATE, exposed within 3 months vs unexposed within 3 months, with adjustment for covariates **eTable 8A.** Results of regression analysis for VTE outcome, each anti-cancer therapy exposure within 3 months vs not receiving any systemic treatments within 3 months, with adjustment for covariates **eTable 8B.** Results of regression analysis for ATE outcome, each anti-cancer therapy exposure within 3 months vs not receiving any systemic treatments within 3 months, with adjustment for covariates **eTable 9A.** Results of regression analysis for VTE outcome, each anti-cancer therapy exposure within 3 months vs not receiving any systemic treatments within 3 months, with adjustment for covariates **eTable 9B.** Results of regression analysis for ATE outcome, each anti-cancer therapy exposure within 3 months vs not receiving any systemic treatments within 3 months, with adjustment for covariates **eTable 10.** Analysis to test for the interaction between the severity of COVID-19 infection (by symptoms of infection) and systemic therapies **eTable 11.** 30 day (column 1) and 90 day (column 2) follow-up outcome **eFigure 1.** Consort diagram **eFigure 2.** Combined VTE/ATE forest plot for the estimates of log odds ratios with the 95% confidence intervals for exposure to the five treatments of interest within 3 months vs. no exposure within 3 months **eFigure 3.** Forest plots for outcome of death after a thromboembolic event **eFigure 4.** Forest plots for the estimates of log risk ratios with the 95% confidence intervals for patients exposed to systemic therapies of interest pooled together within 3 months vs. unexposed within 3 months

Supplement 2.
COVID-19 and Cancer Consortium Nonauthor Collaborators

Supplement 3.
Data Sharing Statement
