# Supplementary material for: Systemic Anticancer Therapy and Thromboembolic Outcomes in Hospitalized Patients With Cancer and COVID-19
Source: JAMA Oncol. 2023 Aug 17;9(10):1390–400. doi: 10.1001/jamaoncol.2023.2934 (PMC10436185; doi:10.1001/jamaoncol.2023.2934)
Supplement: Supplement 1. — eTable 1. Data Dictionary used for this analysis eTable 2. Cancer site classification for the study eTable 3. Patients in each treatment arm who had undergone recent surgery eTable 4A. Variable information eTable 4B. List of drugs included in each exposure category eTable 5. Patients with VTE and ATE: Head-to-head comparison of clinical features in those who were on systemic therapies vs. in those not on these therapies eTable 6. Results of regression analysis for combined VTE/ATE outcome, each anti-cancer therapy exposure within 3 months vs not receiving any systemic treatments within 3 months, with adjustment for covariates eTable 7A. Secondary outcomes in patients in each group (within 3 months) with no ATE/VTE eTable 7B. Results of regression analysis for death outcome after VTE/ATE, exposed within 3 months vs unexposed within 3 months, with adjustment for covariates eTable 8A. Results of regression analysis for VTE outcome, each anti-cancer therapy exposure within 3 months vs not receiving any systemic treatments within 3 months, with adjustment for covariates eTable 8B. Results of regression analysis for ATE outcome, each anti-cancer therapy exposure within 3 months vs not receiving any systemic treatments within 3 months, with adjustment for covariates eTable 9A. Results of regression analysis for VTE outcome, each anti-cancer therapy exposure within 3 months vs not receiving any systemic treatments within 3 months, with adjustment for covariates eTable 9B. Results of regression analysis for ATE outcome, each anti-cancer therapy exposure within 3 months vs not receiving any systemic treatments within 3 months, with adjustment for covariates eTable 10. Analysis to test for the interaction between the severity of COVID-19 infection (by symptoms of infection) and systemic therapies eTable 11. 30 day (column 1) and 90 day (column 2) follow-up outcome eFigure 1. Consort diagram eFigure 2. Combined VTE/ATE forest plot for the estimates of log odds ratios with the 9 [file jamaoncol-e232934-s001.pdf]

Supplemental Online Content

Gulati S, Hsu C, Shah S, et al; for the COVID-19 and Cancer Consortium. Systemic anticancer therapy and thromboembolic outcomes in hospitalized patients with cancer and COVID-19. *JAMA Oncol.* Published online August 17, 2023. doi:10.1001/jamaoncol.2023.2934

eTable 1. Data Dictionary used for this analysis

eTable 2. Cancer site classification for the study

eTable 3. Patients in each treatment arm who had undergone recent surgery

eTable 4A. Variable information

eTable 4B. List of drugs included in each exposure categories

eTable 5. Patients with VTE and ATE: Head-to-head comparison of clinical features in those who were on systemic therapies vs. in those not on these therapies

eTable 6. Results of regression analysis for combined VTE/ATE outcome, each anti-cancer therapy exposure within 3 months vs not receiving any systemic treatments within 3 months, with adjustment for covariates

eTable 7A. Secondary outcomes in patients in each group (within 3 months) with no ATE/VTE

eTable 7B. Results of regression analysis for death outcome after VTE/ATE, exposed within 3 months vs unexposed within 3 months, with adjustment for covariates

eTable 8A. Results of regression analysis for VTE outcome, each anti-cancer therapy exposure within 3 months vs not receiving any systemic treatments within 3 months, with adjustment for covariates

eTable 8B. Results of regression analysis for ATE outcome, each anti-cancer therapy exposure within 3 months vs not receiving any systemic treatments within 3 months, with adjustment for covariates

eTable 9A. Results of regression analysis for VTE outcome, each anti-cancer therapy exposure within 3 months vs not receiving any systemic treatments within 3 months, with adjustment for covariates

© 2023 Gulati S et al. *JAMA Oncology.*

eTable 9B. Results of regression analysis for ATE outcome, each anti-cancer therapy exposure within 3 months vs not receiving any systemic treatments within 3 months, with adjustment for covariates

eTable 10. Analysis to test for the interaction between the severity of COVID-19 infection (by symptoms of infection) and systemic therapies

eTable 11. 30 day (column 1) and 90 day (column 2) follow-up outcome

eFigure 1. Consort diagram

eFigure 2. Combined VTE/ATE forest plot for the estimates of log odds ratios with the 95% confidence intervals for exposure to the five treatments of interest within 3 months vs. no exposure within 3 months

eFigure 3. Forest plots for outcome of death after a thromboembolic event

eFigure 4. Forest plots for the estimates of log risk ratios with the 95% confidence intervals for patients exposed to systemic therapies of interest pooled together within 3 months vs. unexposed within 3 months

This supplemental material has been provided by the authors to give readers additional information about their work.

eTable 1. Data Dictionary used for this analysis
The full CCC19 data dictionary and R code to generate the derived variables is publicly available on GitHub: https://github.com/covidncancer/CCC19_dictionary

Outcome description	Outcome variable name	Outcome values
VTE	der_VTE_comp_v2	0 = No; 1 = Yes; 99 = Unknown
ATE	der_ATE_comp	0 = No; 1 = Yes; 99 = Unknown
30-day all-cause mortality	der_dead30	0 = No; 1 = Yes; 99 = Unknown
ICU admission	der_ICU	0 = No; 1 = Yes; 99 = Unknown
Mechanical ventilation/ pressors	der_MV	0 = No; 1 = Yes; 99 = Unknown
Binary death indicator	der_deadbinary	0 = No; 1 = Yes; 99 = Unknown
VTE within 3-month follow up	der_VTE_comp_within_3mo	0 = No; 1 = Yes; 99 = Unknown
ATE within 3-month follow up	der_ATE_comp_within_3mo	0 = No; 1 = Yes; 99 = Unknown
VTE within 30-day follow up	der_VTE_comp_within_30d	0 = No; 1 = Yes; 99 = Unknown
ATE within 30-day follow up	der_ATE_comp_within_30d	0 = No; 1 = Yes; 99 = Unknown

Exposure description	Variable name	Covariate values
Hospitalization	der_hosp	0 = No; 1 = Yes; 99 = Unknown
Endocrine therapy within 3 months prior to COVID-19	der_any_endo_3mo	0 = No; 1 = Yes; 99 = Unknown
TKI or VEGFi within 3 months prior to COVID-19	der_tki_vegfi_3mo	0 = No; 1 = Yes; 99 = Unknown
Most recent line of therapy includes an IMiD, within 3 months prior to COVID-19	der_imid_3mo	0 = No; 1 = Yes; 99 = Unknown
Most recent line of therapy includes a PD-1 or PD-L1 inhibitor, within 3 months prior to COVID-19	der_pd1_l1_3mo	0 = No; 1 = Yes; 99 = Unknown
Most recent line of therapy includes cytotoxic chemotherapy within 3 months prior to COVID-19	der_any_cyto_3mo	0 = No; 1 = Yes; 99 = Unknown
Any systemic therapy within 3 months prior to COVID-19 (used to define control group)	der_any_systemic_3mo	0 = No; 1 = Yes; 99 = Unknown

Covariate description	Variable name	Covariate values
Age	der_age_trunc	Years, integer 18-90 with 90+ truncated to 90 and 18- truncated to 18
Sex	der_sex	Female; Male
Race	der_race_v2	Hispanic; Non-Hispanic AAPI; Non-Hispanic Black; Non-Hispanic White; Other
BMI (Binary obesity (BMI >= 30)	der_obesity_v2	0 = No; 1 = Yes; 99 = Unknown
Cardiovascular comorbidity (CAD, CHF, Afib, arrhythmia NOS, PVD, CVA, cardiac disease NOS)[1]	der_card	0 = No; 1 = Yes; 99 = Unknown

© 2023 Gulati S et al. *JAMA Oncology.*

Cardiovascular comorbidity (CAD, CVA, PAD)[2]	der_card_v3	0 = No; 1 = Yes; 99 = Unknown
Atrial fibrillation at baseline[2]	der_afib_bl	0 = No; 1 = Yes; 99 = Unknown
Baseline diabetes mellitus, with or without complication[2]	der_dm2	0 = No; 1 = Yes; 99 = Unknown
ECOG Performance status	der_ecogcat2	0; 1; 2+; Unknown
Smoking status	der_smoking2	Never; Current or Former; Unknown
Cancer status	der_cancer_status	0 - Remission/NED; 1 - Active, stable/responding; 2 - Active, progressing; 99 - Unknown
Metastatic cancer status	der_metastatic	0 = No; 1 = Yes; 99 = Unknown
History of prior VTE	der_VTE_baseline	0 = No; 1 = Yes; 99 = Unknown
Risk of VTE	der_VTE_risk_v4	Low/Other; High/Very high
D-Dimer	der_ddimer	Normal; Abnormal; Not drawn/Not available; Unknown
Neutrophil:Lymphocyte ratio, categorical	der_nlr_cat	HI; LO; Neither; Not drawn/Not available
IL6	der_il6	Normal; Abnormal; Not drawn/Not available; Unknown
WBC count	der_wbc	Normal; High; Low; Not drawn/Not available; Unknown
Platelets	der_plt	Normal; High; Low; Not drawn/Not available; Unknown
Baseline anticoagulation with dosing	der_ac_baseline_dose	0 = None; 1 = Prophylactic dosing; 2 = Therapeutic dosing; 99 = Unknown
Baseline aspirin with dosing	der_asa_baseline_dose	0 = None; 1 = Low-dose (<200 mg/day); 2 = Full dose; 99 = Unknown
Baseline antiplatelet agents other than aspirin	der_apa_baseline_v2	0 = No; 1 = Yes; 99 = Unknown
Initial Severity and Course of Illness of COVID-19	severity_of_covid_19_v2	Mild (no hospitalization required); Moderate (hospitalization indicated); Severe (ICU admission indicated)
Surgery	Surgery	Yes: surgery_timing = 1;<br>No: recent_surgery = 0 or surgery_timing %in% c(2, 3, 88);<br>Unknown: recent_surgery = 99 or surgery_timing = UNK

1: Used for VTE analysis

2: Used for ATE analysis

Venous thromboembolism (VTE) definition:

- PE
- DVT
- Thrombosis NOS
- Free text: DVT, PE, (deep) vein thrombosis, venous thrombosis, thromboses, thrombosis, thromboembolism, thrombus, (pulmonary or lung) embolism, (pulmonary or lung) embolus, blood clot, clot, occlusion (if venous).

Arterial Thromboembolism (ATE) definition:

© 2023 Gulati S et al. *JAMA Oncology*.

- Cerebral vascular accident
- Myocardial infarction
- Free text: CVA, stroke, MI, heart attack, PAD

© 2023 Gulati S et al. *JAMA Oncology.*

eTable 2. Cancer site classification for the study (adapted from Khorana score [1])

TUMOR TYPE	Number of patients (N)	VTE (N)	ATE (N)
**High risk cancers N=1675**		159	60
Bladder	148	22	5
Esophagus	38	5	1
Stomach (gastric) cancer	55	3	1
Germ-cell tumors and testicular cancer	35	1	1
Lung cancer	472	42	19
Ovarian Cancer	68	10	1
Pancreatic Cancer	81	8	5
Renal cell cancer	199	17	5
Lymphomas	422	38	18
Uterine cancers	157	13	4
**Others and “low” risk cancers N=3313**		203	138
GI tract cancer (colon, rectum and anal cancers, small intestine cancer, appendix)	390	26	20
Bile Duct Cancer (cholangiocarcinoma) and gall bladder cancer	33	2	0
Brain Cancers (benign e.g., meningioma, low-grade glioma, high-grade glioma (e.g., GBM), neuroblastoma, retinoblastoma)	80	8	6
Breast Cancer	655	49	24
Gynecologic cancers (cervical cancer, fallopian tube cancer, vagina and vulva cancer, placenta cancer (including choriocarcinoma)	71	5	1
Sarcomas and bone cancers (Ewing Sarcoma, soft tissue sarcoma, vascular Sarcoma, GIST, osteosarcoma, rhabdomyosarcoma)	107	10	1
Head and neck cancer, nasopharyngeal carcinoma	154	8	4
Invasive skin cancers (SCC and BCC)	25	1	3

© 2023 Gulati S et al. *JAMA Oncology.*

Mesothelioma and peritoneum cancer	12	0	1
Ill-defined/ cancer of unknown primary	15	2	2
Liver cancer	68	2	3
Melanoma	71	1	3
Merkel Cell	7	0	0
Neuroendocrine tumor (NET), carcinoid	68	5	3
Endocrine cancers (parathyroid Cancer, thyroid, adrenocortical carcinoma)	117	3	1
Penis and scrotum cancer	5	0	0
Prostate cancer	668	33	36
Thymus cancer	11	2	0
Wilms tumor	0	0	0
Acute leukemia (AML, ALL)	176	14	6
Other malignant hematologic neoplasms (myeloproliferative neoplasm, MDS, CML, T-cell and NK-cell neoplasm)	148	5	6
Chronic lymphocytic leukemia (CLL) and lymphoproliferative disorder	176	13	5
Plasma cell dyscrasia, multiple myeloma, AL-amyloidosis	237	13	13
Histiocyte disorders	0	0	0
Malignant Solid Neoplasm, NOS	4	0	0
Others	15	1	0

© 2023 Gulati S et al. *JAMA Oncology.*

eTable 3. Patients in each treatment arm who had undergone recent surgery

Agent / agent group	Total	Endocrine	Tki_Vegfi	IMiDs	ICIs	Chemo	unexposed
	N = 4988	N = 466	N = 332	N = 138	N = 262	N = 1002	N = 3119
**Surgery**							
No	91% (4517)	94% (438)	95% (316)	92% (127)	90% (235)	93% (928)	89% (2781)
Yes	5% (237)	3% (16)	2% (5)	1% (2)	5% (14)	4% (45)	5% (171)
Missing	5% (234)	3% (12)	3% (11)	7% (9)	5% (13)	3% (29)	5% (167)

© 2023 Gulati S et al. *JAMA Oncology.*

eTable 4A. Variable information (N=4988, exposed within 3 months)

Agent / agent group	Total	Endocrine	Tki_Vegfi	IMiDs	ICIs	Cyto	Control
	N = 4988	N = 466	N = 332	N = 138	N = 262	N = 1002	N = 3119
**der_VTE_comp_v2**							
0	93% (4626)	93% (434)	90% (299)	92% (127)	88% (231)	90% (897)	94% (2929)
1	7% (362)	7% (32)	10% (33)	8% (11)	12% (31)	10% (105)	6% (190)
**der_ATE_comp**							
0	96% (4790)	95% (444)	98% (325)	96% (133)	98% (258)	97% (972)	95% (2978)
1	4% (198)	5% (22)	2% (7)	4% (5)	2% (4)	3% (30)	5% (141)
**der_VTE_ATE**							
0	89% (4448)	89% (413)	88% (292)	88% (122)	87% (228)	87% (873)	90% (2801)
1	11% (540)	11% (53)	12% (40)	12% (16)	13% (34)	13% (129)	10% (318)
**der_dead30**							
0	79% (3925)	80% (373)	76% (252)	83% (114)	79% (206)	75% (755)	80% (2481)
1	21% (1056)	20% (93)	23% (77)	17% (24)	21% (56)	24% (243)	20% (637)
Missing	0% (7)	0% (0)	1% (3)	0% (0)	0% (0)	0% (4)	0% (1)
**der_ICU**							
0	70% (3505)	74% (347)	67% (224)	63% (87)	74% (193)	72% (723)	70% (2168)
1	28% (1392)	23% (106)	31% (102)	36% (50)	25% (66)	27% (266)	29% (894)
Missing	2% (91)	3% (13)	2% (6)	1% (1)	1% (3)	1% (13)	2% (57)
**der_mv**							
0	82% (4067)	86% (399)	78% (259)	79% (109)	86% (226)	82% (818)	81% (2529)
1	17% (862)	14% (64)	20% (67)	20% (28)	11% (29)	17% (168)	18% (559)
Missing	1% (59)	1% (3)	2% (6)	1% (1)	3% (7)	2% (16)	1% (31)
**der_age_trunc**							
**der_age_trunc**	59/69/78	61/72/80	54/65/74	59/66.5/74.9	58.3/68/75.8	53/63/72	61/71/79
**der_sex**							
Female	48% (2376)	53% (249)	46% (154)	39% (54)	44% (114)	53% (531)	46% (1440)
Male	52% (2608)	47% (217)	54% (178)	61% (84)	56% (148)	47% (470)	54% (1676)
Missing	0% (4)	0% (0)	0% (0)	0% (0)	0% (0)	0% (1)	0% (3)
**der_race_v2**							
Non-Hispanic White	51% (2533)	45% (210)	50% (165)	38% (52)	57% (150)	46% (465)	53% (1655)
Hispanic	16% (803)	20% (94)	23% (77)	17% (24)	16% (43)	22% (220)	13% (420)
Non-Hispanic AAPI	4% (185)	4% (17)	5% (16)	3% (4)	5% (12)	3% (33)	4% (116)
Non-Hispanic Black	21% (1032)	24% (110)	16% (52)	33% (45)	14% (36)	20% (204)	21% (644)
Other	7% (363)	6% (29)	6% (19)	6% (8)	6% (16)	7% (68)	8% (240)
Missing	1% (72)	1% (6)	1% (3)	4% (5)	2% (5)	1% (12)	1% (44)
**der_obesity_v2**							
0	64% (3168)	59% (274)	66% (218)	64% (88)	74% (195)	68% (682)	62% (1942)
1	36% (1796)	41% (191)	33% (108)	36% (50)	26% (67)	32% (316)	37% (1163)
Missing	0% (24)	0% (1)	2% (6)	0% (0)	0% (0)	0% (4)	0% (14)
**der_card**							

© 2023 Gulati S et al. *JAMA Oncology.*

0	62% (3094)	60% (278)	70% (233)	76% (105)	69% (181)	74% (743)	58% (1796)
1	37% (1869)	40% (187)	28% (92)	24% (33)	31% (81)	25% (254)	42% (1310)
Missing	1% (25)	0% (1)	2% (7)	0% (0)	0% (0)	0% (5)	0% (13)
**der_card_v3**							
0	77% (3842)	76% (355)	83% (277)	86% (119)	84% (219)	85% (853)	74% (2297)
1	22% (1121)	24% (110)	14% (48)	14% (19)	16% (43)	14% (144)	26% (809)
Missing	1% (25)	0% (1)	2% (7)	0% (0)	0% (0)	0% (5)	0% (13)
**der_afib_bl**							
0	87% (4337)	86% (401)	89% (297)	94% (130)	90% (235)	92% (918)	85% (2658)
1	13% (626)	14% (64)	8% (28)	6% (8)	10% (27)	8% (79)	14% (448)
Missing	1% (25)	0% (1)	2% (7)	0% (0)	0% (0)	0% (5)	0% (13)
**der_renal**							
0	80% (3967)	80% (373)	85% (281)	80% (111)	85% (223)	87% (876)	77% (2387)
1	20% (996)	20% (92)	13% (44)	20% (27)	15% (39)	12% (121)	23% (719)
Missing	1% (25)	0% (1)	2% (7)	0% (0)	0% (0)	0% (5)	0% (13)
**der_dm2**							
0	66% (3311)	63% (292)	70% (232)	69% (95)	72% (189)	70% (705)	65% (2025)
1	33% (1652)	37% (173)	28% (93)	31% (43)	28% (73)	29% (292)	35% (1081)
Missing	1% (25)	0% (1)	2% (7)	0% (0)	0% (0)	0% (5)	0% (13)
**der_ecogcat2**							
0	24% (1182)	28% (129)	25% (83)	25% (35)	18% (47)	21% (210)	24% (745)
1	27% (1350)	29% (136)	38% (126)	36% (49)	47% (124)	40% (402)	21% (645)
2+	21% (1045)	26% (120)	20% (68)	20% (27)	26% (68)	25% (248)	19% (590)
Unknown	28% (1406)	17% (81)	16% (54)	20% (27)	8% (22)	14% (141)	36% (1136)
Missing	0% (5)	0% (0)	0% (1)	0% (0)	0% (1)	0% (1)	0% (3)
**der_smoking2**							
Never	47% (2351)	47% (221)	55% (184)	57% (78)	34% (89)	49% (492)	46% (1431)
Current or Former	49% (2454)	48% (225)	39% (131)	37% (51)	65% (169)	48% (476)	51% (1578)
Missing	4% (183)	4% (20)	5% (17)	7% (9)	2% (4)	3% (34)	4% (110)
**der_cancer_status**							
0 - Remission/NED	46% (2296)	30% (140)	10% (34)	16% (22)	3% (9)	9% (89)	65% (2021)
1 - Active, stable/responding	27% (1341)	43% (200)	52% (174)	63% (87)	51% (134)	42% (423)	15% (478)
2 - Active, progressing	17% (838)	20% (94)	27% (91)	11% (15)	32% (85)	34% (341)	10% (325)
99 - Unknown	10% (510)	7% (32)	10% (33)	10% (14)	13% (34)	15% (148)	9% (293)
Missing	0% (3)	0% (0)	0% (0)	0% (0)	0% (0)	0% (1)	0% (2)
**der_metastatic**							
0	64% (3172)	47% (218)	26% (86)	22% (30)	21% (55)	34% (338)	81% (2511)
1	31% (1541)	48% (225)	69% (229)	62% (86)	73% (191)	60% (603)	15% (460)
Missing	6% (275)	5% (23)	5% (17)	16% (22)	6% (16)	6% (61)	5% (148)
**der_VTE_baseline**							
0	89% (4426)	89% (415)	85% (282)	86% (118)	89% (232)	85% (851)	90% (2813)
1	11% (537)	11% (50)	13% (43)	14% (20)	11% (30)	15% (146)	9% (293)
Missing	1% (25)	0% (1)	2% (7)	0% (0)	0% (0)	0% (5)	0% (13)

© 2023 Gulati S et al. *JAMA Oncology.*

**der_VTE_risk_v4**							
Low/Other	66% (3313)	94% (437)	69% (229)	92% (127)	34% (89)	57% (575)	66% (2059)
High/Very high	34% (1675)	6% (29)	31% (103)	8% (11)	66% (173)	43% (427)	34% (1060)
Missing	0% (0)	0% (0)	0% (0)	0% (0)	0% (0)	0% (0)	0% (0)
**der_ddimer**							
Normal	8% (409)	8% (37)	9% (29)	12% (16)	8% (22)	6% (62)	8% (263)
Abnormal	45% (2242)	53% (247)	42% (139)	40% (55)	38% (100)	43% (433)	45% (1416)
Unknown	8% (399)	8% (39)	12% (39)	12% (17)	9% (23)	12% (117)	7% (204)
Missing	39% (1938)	31% (143)	38% (125)	36% (50)	45% (117)	39% (390)	40% (1236)
**der_nlr_cat**							
HI	49% (2434)	50% (231)	46% (154)	53% (73)	55% (145)	49% (493)	49% (1521)
LO	3% (165)	2% (9)	6% (20)	4% (5)	2% (4)	3% (30)	4% (111)
Neither	29% (1435)	31% (143)	32% (106)	30% (41)	22% (58)	30% (302)	28% (869)
Missing	19% (954)	18% (83)	16% (52)	14% (19)	21% (55)	18% (177)	20% (618)
**der_il6**							
Normal	2% (93)	2% (8)	1% (4)	1% (1)	3% (7)	2% (16)	2% (61)
Abnormal	11% (559)	14% (63)	16% (52)	17% (23)	12% (31)	15% (148)	10% (304)
Unknown	14% (701)	14% (67)	17% (56)	19% (26)	19% (51)	17% (168)	13% (399)
Missing	73% (3635)	70% (328)	66% (220)	64% (88)	66% (173)	67% (670)	76% (2355)
**der_wbc**							
Normal	54% (2685)	61% (284)	48% (161)	49% (67)	47% (123)	42% (416)	58% (1794)
High	16% (796)	12% (55)	19% (63)	3% (4)	18% (47)	15% (151)	17% (527)
Low	19% (935)	17% (78)	21% (70)	40% (55)	19% (50)	33% (335)	14% (436)
Missing	11% (572)	11% (49)	11% (38)	9% (12)	16% (42)	10% (100)	12% (362)
**der_plt**							
Normal	57% (2831)	64% (298)	50% (165)	43% (60)	48% (126)	46% (461)	61% (1889)
High	4% (208)	3% (12)	3% (10)	3% (4)	6% (16)	5% (48)	4% (135)
Low	27% (1323)	21% (98)	34% (114)	44% (61)	29% (75)	38% (376)	23% (707)
Missing	13% (626)	12% (58)	13% (43)	9% (13)	17% (45)	12% (117)	12% (388)
**der_ac_baseline_dose**							
0	76% (3794)	73% (339)	72% (240)	75% (103)	74% (193)	72% (721)	78% (2426)
1	9% (445)	9% (42)	10% (32)	11% (15)	12% (31)	12% (118)	8% (252)
2	10% (518)	13% (59)	13% (44)	11% (15)	11% (29)	11% (115)	10% (301)
Missing	5% (231)	6% (26)	5% (16)	4% (5)	3% (9)	5% (48)	4% (140)
**der_asa_baseline_dose**							
0	69% (3418)	65% (304)	80% (264)	45% (62)	75% (196)	80% (806)	65% (2033)
1	28% (1397)	32% (148)	18% (59)	47% (65)	23% (60)	17% (173)	31% (967)
2	2% (92)	2% (7)	1% (2)	7% (9)	1% (2)	1% (9)	2% (65)
Missing	2% (81)	2% (7)	2% (7)	1% (2)	2% (4)	1% (14)	2% (54)
**der_apa_baseline_v2**							
0	93% (4655)	93% (433)	95% (314)	96% (133)	97% (254)	96% (965)	92% (2871)
1	6% (284)	6% (30)	4% (12)	4% (5)	3% (7)	3% (28)	7% (215)
Missing	1% (49)	1% (3)	2% (6)	0% (0)	0% (1)	1% (9)	1% (33)

© 2023 Gulati S et al. *JAMA Oncology.*

der_ac_c19_dose							
0	24% (1206)	23% (108)	27% (90)	17% (23)	25% (65)	27% (266)	23% (730)
1	51% (2545)	54% (250)	47% (155)	51% (71)	46% (120)	46% (462)	53% (1645)
2	18% (896)	20% (92)	19% (64)	20% (27)	19% (49)	19% (186)	18% (549)
88	1% (55)	0% (0)	1% (3)	4% (5)	1% (3)	1% (9)	1% (38)
Missing	6% (286)	3% (16)	6% (20)	9% (12)	10% (25)	8% (79)	5% (157)
**der_cancer_tx_timing_v2**							
0	46% (2319)	0% (0)	0% (0)	0% (0)	0% (0)	0% (0)	74% (2319)
1	34% (1719)	84% (392)	87% (289)	91% (126)	85% (223)	82% (818)	5% (152)
2	8% (419)	16% (74)	13% (43)	9% (12)	15% (39)	18% (184)	4% (117)
88	11% (531)	0% (0)	0% (0)	0% (0)	0% (0)	0% (0)	17% (531)
Missing	0% (0)	0% (0)	0% (0)	0% (0)	0% (0)	0% (0)	0% (0)

© 2023 Gulati S et al. *JAMA Oncology.*

eTable 4B. List of drugs included in each exposure categories

1. Endocrine therapy: Prostate cancer: Gonadotrophin release hormone (GnRH) agonists, GnRH antagonists, orchiectomy); Breast cancer: aromatase inhibitors, tamoxifen
2. Targeted therapies: antiangiogenic therapies (vascular endothelial growth factor receptor inhibitors, tyrosine kinase inhibitors)
3. Immunomodulatory drugs (IMiDs): Thalidomide, Lenalidomide, Pomalidomide
4. Immune checkpoint inhibitors (ICIs): Nivolumab, Pembrolizumab, Atezolizumab, Avelumab, Durvalumab, Cemiplimab

Drugs were not mutually exclusive. For example 29 patients were on both TKI and IO (see below)

© 2023 Gulati S et al. *JAMA Oncology.*

eTable 5. Patients with VTE and ATE: Head-to-head comparison of clinical features in those who were on systemic therapies vs. in those not on these therapies

	VTE in exposed (N= 172)	VTE in unexposed (N= 190)	ATE in exposed (N=57)	ATE in unexposed (N=141)
**Median age (years, IQR)**	66 (56-73)	67 (60-77)	70 (61-78)	72 (66-80)
**Sex**				
Female	49% (85)	48% (92)	51% (29)	36% (51)
Male	51% (87)	52% (98)	49% (28)	64% (90)
Missing	0% (0)	0% (0)	0% (0)	0% (0)
**Race**				
Non-Hispanic White	47% (81)	51% (96)	53% (30)	52% (73)
Hispanic	14% (24)	14% (26)	11% (6)	11% (15)
Non-Hispanic AAPI	4% (7)	2% (4)	0% (0)	3% (4)
Non-Hispanic Black	28% (48)	24% (45)	26% (15)	26% (37)
Other	5% (9)	8% (16)	11% (6)	8% (11)
Missing	2% (3)	2% (3)	0% (0)	1% (1)
**BMI ≥ 30 kg/m2**				
No	62% (106)	59% (112)	72% (41)	67% (95)
Yes	38% (66)	40% (76)	28% (16)	33% (46)
Missing	0% (0)	1% (2)	0% (0)	0% (0)
**Cardiovascular comorbidity** [1]				
No	78% (134)	67% (128)	53% (30)	41% (58)
Yes	22% (38)	32% (60)	47% (27)	59% (83)
Missing	0% (0)	1% (2)	0% (0)	0% (0)
**History of arterial thromboembolic events CAD, CVA, PAD)**				
No	91% (156)	82% (156)	63% (36)	62% (88)
Yes	9% (16)	17% (32)	37% (21)	38% (53)
Missing	0% (0)	1% (2)	0% (0)	0% (0)
**History of atrial fibrillation**				
No	94% (162)	92% (175)	88% (50)	83% (117)
Yes	6% (10)	7% (13)	12% (7)	17% (24)
Missing	0% (0)	1% (2)	0% (0)	0% (0)
**History of renal disease**				
No	90% (154)	83% (157)	79% (45)	71% (100)
Yes	10% (18)	16% (31)	21% (12)	29% (41)
Missing	0% (0)	1% (2)	0% (0)	0% (0)
**History of diabetes mellites**				
No	72% (123)	67% (128)	63% (36)	58% (82)
Yes	28% (49)	32% (60)	37% (21)	42% (59)
Missing	0% (0)	1% (2)	0% (0)	0% (0)
**ECOG performance status**				
0	20% (35)	34% (64)	14% (8)	18% (26)
1	40% (68)	21% (40)	32% (18)	26% (36)
2+	26% (45)	13% (24)	44% (25)	23% (32)
Unknown	14% (24)	33% (62)	11% (6)	33% (47)
Missing	0% (0)	0% (0)	0% (0)	0% (0)
**History of smoking**				
Never	47% (81)	53% (100)	51% (29)	42% (59)
Current or Former	49% (85)	46% (88)	47% (27)	55% (78)
Missing	3% (6)	1% (2)	2% (1)	3% (4)
**Cancer status**				
Remission/NED	10% (18)	60% (114)	12% (7)	62% (88)
Active, stable/responding	37% (63)	16% (31)	49% (28)	13% (19)
Active, progressing	37% (64)	13% (24)	23% (13)	8% (11)
Unknown	16% (27)	11% (21)	16% (9)	16% (23)
Missing	0% (0)	0% (0)	0% (0)	0% (0)
**Presence of metastatic cancer**				
No	30% (52)	80% (152)	26% (15)	79% (111)
Yes	65% (112)	16% (30)	56% (32)	16% (22)
Missing	5% (8)	4% (8)	18% (10)	6% (8)
**History of VTE at baseline**				
No	66% (114)	76% (144)	82% (47)	92% (130)

© 2023 Gulati S et al. *JAMA Oncology.*

Yes	34% (58)	23% (44)	18% (10)	8% (11)
Missing	0% (0)	1% (2)	0% (0)	0% (0)
**Risk of VTE based on site of cancer adapted from Khorana Score [2]**				
Low/Other	55% (94)	57% (109)	74% (42)	68% (96)
High/Very high	45% (78)	43% (81)	26% (15)	32% (45)
Missing	0% (0)	0% (0)	0% (0)	0% (0)
**D-Dimer**				
Normal	5% (9)	5% (10)	7% (4)	9% (12)
Abnormal	51% (87)	54% (102)	51% (29)	45% (64)
Unknown	8% (13)	6% (12)	5% (3)	6% (8)
Missing	37% (63)	35% (66)	37% (21)	40% (57)
**Neutrophil/ lymphocyte ratio**				
High	47% (81)	57% (108)	60% (34)	43% (61)
Low	3% (6)	2% (4)	0% (0)	4% (6)
Neither	29% (50)	26% (49)	28% (16)	30% (42)
Missing	20% (35)	15% (29)	12% (7)	23% (32)
**IL-6 level**				
Normal	3% (5)	1% (2)	4% (2)	1% (1)
Abnormal	10% (17)	13% (24)	11% (6)	10% (14)
Unknown	20% (35)	13% (25)	5% (3)	9% (12)
Missing	67% (115)	73% (139)	81% (46)	81% (114)
**WBC Count**				
Normal	49% (84)	62% (118)	51% (29)	65% (91)
High	16% (27)	19% (37)	14% (8)	18% (25)
Low	24% (41)	11% (21)	30% (17)	10% (14)
Missing	12% (20)	7% (14)	5% (3)	8% (11)
**Platelet count**				
Normal	48% (83)	66% (126)	51% (29)	62% (88)
High	7% (12)	5% (9)	4% (2)	2% (3)
Low	33% (57)	22% (41)	40% (23)	28% (39)
Missing	12% (20)	7% (14)	5% (3)	8% (11)
**Baseline anticoagulation**				
None	65% (112)	73% (138)	74% (42)	79% (112)
Prophylactic dosing	8% (13)	9% (17)	9% (5)	9% (13)
Therapeutic dosing	22% (37)	14% (27)	9% (5)	9% (13)
Missing	6% (10)	4% (8)	9% (5)	2% (3)
**Baseline use of aspirin**				
None	85% (146)	75% (142)	61% (35)	52% (73)
Low-dose (<200mg)	14% (24)	24% (46)	37% (21)	45% (63)
Full dose	1% (1)	1% (1)	0% (0)	3% (4)
Missing	1% (1)	1% (1)	2% (1)	1% (1)
**Baseline anti-platelet agents**				
No	98% (169)	94% (178)	88% (50)	87% (122)
yes	1% (2)	6% (11)	12% (7)	13% (18)
Missing	1% (1)	1% (1)	0% (0)	1% (1)

© 2023 Gulati S et al. *JAMA Oncology.*

eTable 6
Results of regression analysis for combined VTE/ATE outcome, each anti-cancer therapy exposure within 3 months vs not receiving any systemic treatments within 3 months, with adjustment for covariates

Treatment	aRR for ATE/VTE within 3 months of treatment [a]	95% CI
**Pooled**	1.10	0.91-1.33
**Endocrine**	**1.17**	**0.89-1.54**
**TKI_VEGFi**	**1.12**	**0.82-1.52**
**IMid**	**1**.09	**0.67-1.77**
**ICI**	1.11	0.80-1.55
**Chemotherapy**	**1.14**	**0.93-1.40**

[a] Adjusted for age, sex, race, obesity (defined as BMI >=30), Eastern Cooperative Oncology Group (ECOG) performance status, cancer status at the time of COVID-19 diagnosis (active, stable/responding, active/ progressing, unknown), metastatic cancer status, prior history of VTE, cancer type by VTE risk, prior anticoagulation use, prior aspirin use, and prior antiplatelet agent use.

© 2023 Gulati S et al. *JAMA Oncology.*

eTable 7A. Secondary outcomes in patients in each group (within 3 months) with no ATE/ VTE. Secondary outcomes of 30-day mortality, rate of admission to the intensive care unit and rate of mechanical ventilation in patients who develop ATE/ VTE in a) the entire cohort b) in those exposed to individual systemic therapies

SECONDARY OUTCOME	Total Cohort (%, 95%CI)	Endocrine (%, 95%CI)	VEGFi/TKIs (%, 95%CI)	IMiDs (%, 95%CI)	ICIs (%, 95%CI)	Chemotherapy (%, 95%CI)
**30-day mortality**	25% (0.21-0.29)	17% (0.08-0.28)	35% (0.20-0.50)	12% (0.00-0.31)	21% (0.09-0.35)	30% (0.22-0.38)
**ICU admission**	46% (0.42-0.51)	37% (0.25-0.50)	50% (0.35-0.65)	62% (0.38-0.88)	38% (0.23-0.56)	41% (0.33-0.50)
**Mechanical ventilation**	31% (0.27-0.35)	17% (0.08-0.28)	42% (0.26-0.58)	25% (0.06-0.44)	15% (0.03-0.27)	30% (0.22-0.39)

© 2023 Gulati S et al. *JAMA Oncology.*

eTable 7B. Results of regression analysis for death outcome after VTE/ATE, exposed within 3 months vs unexposed within 3 months, with adjustment for covariates (N = 537)

Characteristics	Multivariable aRR (95% CI)	SE of RR
Exposed to TOIs vs not on any systemic therapies	1.12 (0.91-1.38)	0.120
Age, per 10 years increase	1.01 (0.95-1.07)	0.031
Sex (Male vs Female)	1.12 (0.95-1.31)	0.092
Race (Hispanic vs Non-Hispanic White)	0.69 (0.49-0.97)	0.121
Race1 (Non-Hispanic AAPI vs Non-Hispanic White)	0.89 (0.64-1.24)	0.150
Race2 (Non-Hispanic Black vs Non-Hispanic White)	0.98 (0.83-1.17)	0.088
Race3 (Other vs Non-Hispanic White)	1.01 (0.77-1.31)	0.136
BMI > 30 (yes vs no)	0.91 (0.75-1.11)	0.091
ECOG status (1 vs 0)	1.44 (1.05-1.97)	0.231
ECOG status (2 vs 0)	1.77 (1.30-2.40)	0.277
Unknown	1.28 (0.92-1.80)	0.221
Cancer status (Active, stable/responding vs Remission/NED)	0.73 (0.51-1.03)	0.129
Cancer status2 (Active, progressing vs Remission/NED)	1.55 (1.13-2.13)	0.253
Cancer status3 (Unknown vs Remission/NED)	1.10 (0.79-1.54)	0.187
Metastatic cancer (yes vs. no)	1.11 (0.84-1.47)	0.160
History of VTE at baseline (yes vs. no)	0.78 (0.62-0.98)	0.091
Risk of VTE based on site (High/Very high vs Low/Other)	0.95 (0.81-1.12)	0.078
Anticoagulation at baseline (prophylactic vs. none)	0.88 (0.62-1.26)	0.162
Anticoagulation at baseline (therapeutic vs. none)	1.04 (0.83-1.32)	0.125
Baseline ASA (low dose vs none)	0.99 (0.80-1.23)	0.108
Baseline ASA (high dose vs none)	1.02 (0.44-2.37)	0.440
Baseline antiplatelet (yes vs none)	1.34 (1.03-1.74)	0.179

© 2023 Gulati S et al. *JAMA Oncology.*

eTable 8A. Results of regression analysis for VTE outcome, each anti-cancer therapy exposure within 3 months vs not receiving any systemic treatments within 3 months, with adjustment for covariates. This analysis excluded ATE cases. This analysis included recent surgery as an additional variable.

Characteristics	Multivariable aRR (95% CI)	SE of RR
**Exposure to endocrine therapy in past 3 months vs. no exposure**	1.20 (0.83-1.73)	0.225
**Exposure to VEGFi/TKI in past 3 months vs. no exposure**	1.31 (0.92-1.85)	0.233
**Exposure to IMIDs in past 3 months vs. no exposure**	1.35 (0.75-2.45)	0.410
**Exposure to ICIs in past 3 months vs. no exposure**	1.44 (1.01-2.07)	0.265
**Exposure to chemotherapy in past 3 months vs. no exposure**	1.27 (0.99-1.62)	0.160
**Surgery (ref = No)**		
**Yes**	0.99 (0.62-1.57)	0.233
**Age, per 10 years increase**	1.02 (0.95-1.11)	0.041
**Sex** (ref = Female)		
Male	1.00 (0.82-1.23)	0.104
**Race** (ref = Non-Hispanic White)		
Hispanic	0.93 (0.68-1.26)	0.146
Non-Hispanic AAPI	0.82 (0.44-1.52)	0.258
Non-Hispanic Black	1.27 (0.99-1.62)	0.159
Other	0.99 (0.66-1.50)	0.209
**BMI ≥** (ref = 0)		
1	1.14 (0.92-1.41)	0.124
**ECOG performance status** (ref = 0)		
1	0.76 (0.58-1.00)	0.105
2+	0.61 (0.44-0.83)	0.097
Unknown	0.74 (0.56-0.99)	0.108
**Cancer status** (ref = Remission/NED)		
1 - Active, stable/responding	1.00 (0.72-1.38)	0.164
2 - Active, progressing	1.41 (0.99-2.02)	0.257
99 - Unknown	1.56 (1.10-2.22)	0.279
**Cancer status** (ref = non-metastatic)		
1	0.99 (0.73-1.33)	0.151
**History of baseline VTE** (ref = No)		
1	3.10 (2.39-4.04)	0.418
**High risk for VTE per site** (ref = Low/Other)		
High/Very high	1.42 (1.14-1.75)	0.154
**On anticoagulation at baseline** (ref = No)		
Prophylactic	0.82 (0.57-1.19)	0.154
Therapeutic	0.95 (0.70-1.30)	0.150
**On baseline aspirin** (ref = No)		
Low dose	0.69 (0.53-0.91)	0.096
High dose	0.39 (0.10-1.55)	0.274
**On baseline anti-platelet agent** (ref = No)		

© 2023 Gulati S et al. *JAMA Oncology.*

eTable 8B. Results of regression analysis for ATE outcome, each anti-cancer therapy exposure within 3 months vs not receiving any systemic treatments within 3 months, with adjustment for covariates. This analysis excluded ATE cases. This analysis included recent surgery as an additional variable.

Characteristics	Multivariable aRR (95% CI)	SE of RR
**Exposure to endocrine therapy in past 3 months vs. no exposure**	1.23 (0.78-1.96)	0.291
**Exposure to VEGFi/TKI in past 3 months vs. no exposure**	0.79 (0.36-1.69)	0.308
**Exposure to IMIDs in past 3 months vs. no exposure**	1.01 (0.43-2.40)	0.446
**Exposure to ICIs in past 3 months vs. no exposure**	0.39 (0.12-1.22)	0.226
**Exposure to chemotherapy in past 3 months vs. no exposure**	0.85 (0.54-1.35)	0.199
**Surgery (ref = No)**		
**Yes**	0.90 (0.45-1.79)	0.317
**Age, per 10 years increase**	1.11 (0.98-1.26)	0.071
**Sex** (ref = Female)		
Male	1.20 (0.89-1.61)	0.180
**Race** (ref = Non-Hispanic White)		
Hispanic	0.88 (0.54-1.42)	0.215
Non-Hispanic AAPI	0.47 (0.15-1.46)	0.272
Non-Hispanic Black	1.22 (0.86-1.75)	0.222
Other	1.27 (0.75-2.15)	0.342
**History of diabetes mellitus** (ref = No)		
Yes	1.15 (0.85-1.56)	0.177
**History of cardiovascular risk factors** (ref = No)		
Yes	1.34 (0.95-1.88)	0.233
**History of atrial fibrillation** (ref = No)		
Yes	1.18 (0.78-1.81)	0.255
**Cancer status** (ref = Remission/NED)		
Active, stable/responding	0.96 (0.64-1.43)	0.197
Active, progressing	0.91 (0.55-1.51)	0.234
Unknown	2.00 (1.33-3.01)	0.417
**On anticoagulation at baseline** (ref = No)		
Prophylactic	1.12 (0.68-1.85)	0.286
Therapeutic	0.82 (0.47-1.42)	0.229
**On baseline aspirin** (ref = No)		
Low dose	1.50 (1.09-2.07)	0.246
High dose	1.09 (0.41-2.91)	0.547
**On baseline anti-platelet agent** (ref = No)		
Yes	1.64 (1.05-2.56)	0.373

© 2023 Gulati S et al. *JAMA Oncology.*

eTable 9A. Results of regression analysis for VTE outcome, each anti-cancer therapy exposure within 3 months vs not receiving any systemic treatments within 3 months, with adjustment for covariates. This analysis excluded ATE cases. This analysis excluded patients (N=319; 14%) who were on more than one systemic therapy at the time data was collected.

Characteristics	Multivariable aRR (95% CI)	SE of RR	VIF
**Exposure to endocrine therapy in past 3 months vs. no exposure**	1.26 (0.83-1.93)	0.273	1.16
**Exposure to VEGFi/TKI in past 3 months vs. no exposure**	1.30 (0.80-2.11)	0.323	1.16
**Exposure to IMIDs in past 3 months vs. no exposure**	1.48 (0.76-2.85)	0.496	1.11
**Exposure to ICIs in past 3 months vs. no exposure**	1.48 (0.89-2.48)	0.389	1.19
**Exposure to chemotherapy in past 3 months vs. no exposure**	1.22 (0.89-1.66)	0.193	1.45
**Age, per 10 years increase**	1.01 (0.93-1.10)	0.042	1.24
**Sex** (ref = Female)			
Male	0.91 (0.74-1.14)	0.101	1.04
**Race (**ref = Non-Hispanic White)			
Hispanic	0.87 (0.62-1.22)	0.148	1.19
Non-Hispanic AAPI	0.54 (0.24-1.21)	0.222	1.03
Non-Hispanic Black	1.22 (0.94-1.59)	0.163	1.16
Other	0.98 (0.64-1.51)	0.214	1.08
**BMI ≥** (ref = 0)			
1	1.07 (0.86-1.34)	0.123	1.08
**ECOG performance status** (ref = 0)			
1	0.73 (0.55-0.97)	0.107	1.48
2+	0.56 (0.40-0.79)	0.097	1.45
Unknown	0.73 (0.54-0.98)	0.110	1.46
**Cancer status** (ref = Remission/NED)			
1 - Active, stable/responding	0.98 (0.70-1.38)	0.171	1.85
2 - Active, progressing	1.46 (1.01-2.13)	0.279	2.31
99 - Unknown	1.47 (1.02-2.14)	0.279	1.42
**Cancer status** (ref = non-metastatic)			
1	1.00 (0.74-1.36)	0.155	1.91
**History of baseline VTE (**ref = No)			
1	3.01 (2.27-3.99)	0.434	1.48
**High risk for VTE per site** (ref = Low/Other)			
High/Very high	1.46 (1.17-1.84)	0.169	1.12
**On anticoagulation at baseline** (ref = No)			
Prophylactic	0.87 (0.59-1.28)	0.172	1.13
Therapeutic	0.89 (0.63-1.25)	0.154	1.49
**On baseline aspirin** (ref = No)			
Low dose	0.70 (0.53-0.93)	0.102	1.11
High dose	0.38 (0.10-1.51)	0.267	1.01
**On baseline anti-platelet agent** (ref = No)			

© 2023 Gulati S et al. *JAMA Oncology.*

eTable 9B. Results of regression analysis for ATE outcome, each anti-cancer therapy exposure within 3 months vs not receiving any systemic treatments within 3 months, with adjustment for covariates. This analysis excluded ATE cases. <u>This analysis excluded patients (N=319; 14%) who were on more than one systemic therapy at the time data was collected.</u>

Characteristics	Multivariable aRR (95% CI)	SE of RR	VIF
**Exposure to endocrine therapy in past 3 months vs. no exposure**	1.02 (0.60-1.73)	0.274	1.11
**Exposure to VEGFi/TKI in past 3 months vs. no exposure**	0.43 (0.13-1.36)	0.253	1.04
**Exposure to IMIDs in past 3 months vs. no exposure**	1.06 (0.45-2.51)	0.466	1.08
**Exposure to ICIs in past 3 months vs. no exposure**	0.42 (0.10-1.68)	0.297	1.03
**Exposure to chemotherapy in past 3 months vs. no exposure**	0.65 (0.37-1.15)	0.188	1.17
**Age, per 10 years increase**	1.09 (0.96-1.23)	0.071	1.21
**Sex (**ref = Female)			
Male	1.24 (0.92-1.68)	0.193	1.03
**Race** (ref = Non-Hispanic White)			
Hispanic	0.93 (0.57-1.53)	0.235	1.18
Non-Hispanic AAPI	0.51 (0.16-1.56)	0.290	1.02
Non-Hispanic Black	1.26 (0.88-1.82)	0.234	1.19
Other	1.34 (0.79-2.28)	0.363	1.09
**History of diabetes mellitus** (ref = No)			
Yes	1.19 (0.88-1.62)	0.188	1.09
**History of cardiovascular risk factors** (ref = No)			
Yes	1.26 (0.89-1.79)	0.226	1.36
**History of atrial fibrillation** (ref = No)			
Yes	1.18 (0.76-1.82)	0.261	1.25
**Cancer status (**ref = Remission/NED)			
Active, stable/responding	1.10 (0.73-1.66)	0.231	1.36
Active, progressing	0.95 (0.56-1.62)	0.259	1.18
Unknown	2.12 (1.41-3.20)	0.446	1.19
**On anticoagulation at baseline** (ref = No)			
Prophylactic	1.15 (0.70-1.90)	0.292	1.08
Therapeutic	0.82 (0.47-1.43)	0.234	1.17
**On baseline aspirin** (ref = No)			
Low dose	1.53 (1.10-2.14)	0.259	1.24
High dose	1.18 (0.44-3.13)	0.588	1.05
**On baseline anti-platelet agent** (ref = No)			
Yes	1.66 (1.05-2.61)	0.386	1.19

© 2023 Gulati S et al. *JAMA Oncology.*

eTable 10

Since this registry only collected data on cancer patients, and we would not be able to understand the contribution of systemic therapies, we performed an analysis to test for the interaction between the severity of COVID-19 infection (by symptoms of infection), and systemic therapies as shown below.

Statistical approach:

To test if there was an interaction between the initial severity of COVID-19 infection and TOIs for the risk of VTE and ATE, we performed multivariable regression analyses and included the interaction terms between the severity and the pooled TOIs in the regression models. The 95% CIs for the aRRs of the interaction terms were used to determine the significance of the interactions. If the 95% CIs did not cover 1, we would claim there might be significant interactions. Otherwise, there was no strong evidence to prove the existence of interactions. It can be seen from Supplemental Table 9A and 9B that all of the 95% CIs covered 1, indicating there were no significant interaction between the initial severity of COVID-19 infection and TOIs for the risk of VTE and ATE.

A) Interaction between baseline severity of COVID-19 infection and pooled anti-cancer therapy in the VTE model:

Characteristics	Multivariable aRR (95% CI)	SE of RR
Severity of Covid-19 (Moderate vs Mild)	0.91 (0.62-1.33)	0.177
Severity of Covid-19 (Severe vs Mild)	1.91 (1.27-2.87)	0.397
Exposed vs unexposed	1.52 (0.97-2.38)	0.347
Age, per 10 years increase	1.01 (0.94-1.09)	0.039
Sex (Male vs Female)	0.96 (0.78-1.18)	0.099
Race (Hispanic vs Non-Hispanic White)	0.90 (0.66-1.23)	0.142
Race1 (Non-Hispanic AAPI vs Non-Hispanic White)	0.83 (0.45-1.54)	0.261
Race2 (Non-Hispanic Black vs Non-Hispanic White)	1.21 (0.94-1.55)	0.153
Race3 (Other vs Non-Hispanic White)	0.99 (0.65-1.49)	0.208
BMI $\geq$30 (yes vs no)	1.09 (0.88-1.35)	0.118
ECOG status (1 vs 0)	0.76 (0.58-0.99)	0.104
ECOG status2 (2+ vs 0)	0.60 (0.44-0.81)	0.095
ECOG status3 (unknown vs 0)	0.73 (0.55-0.97)	0.106
Cancer status (Active, stable/responding vs Remission/NED)	1.03 (0.75-1.42)	0.168
Cancer status2 (Active, progressing vs Remission/NED)	1.44 (1.02-2.04)	0.254
Cancer status3 (Unknown vs Remission/NED)	1.53 (1.09-2.16)	0.269
Metastatic cancer (yes vs. no)	1.01 (0.77-1.33)	0.142
History of VTE at baseline (yes vs. no)	3.03 (2.33-3.93)	0.403
Risk of VTE based on site (High vs Low/Other)	1.44 (1.17-1.76)	0.148

© 2023 Gulati S et al. *JAMA Oncology.*

Anticoagulation at baseline (prophylactic vs. none)	0.85 (0.60-1.22)	0.156
Anticoagulation at baseline (therapeutic vs. none)	0.95 (0.70-1.30)	0.150
Baseline ASA (low dose vs none)	0.69 (0.52-0.90)	0.095
Baseline ASA (high dose vs none)	0.40 (0.10-1.57)	0.280
Baseline antiplatelet (yes vs none)	0.79 (0.45-1.39)	0.229
**Severity: Moderate (exposed to TOIs in previous 3 months)**	**0.90 (0.54-1.51)**	**0.236**
**Severity: Severe (exposed)**	**0.79 (0.44-1.41)**	**0.234**

B) Interaction between baseline severity of COVID-19 infection and pooled anti-cancer therapy in the ATE model:

**Characteristics**	**Multivariable aRR (95% CI)**	**SE of RR**
Severity of Covid-19 (Moderate vs Mild)	1.30 (0.77-2.20)	0.348
Severity of Covid-19 (Severe vs Mild)	2.50 (1.43-4.38)	0.714
Exposed vs unexposed	1.15 (0.53-2.47)	0.448
Age, per 10 years increase	1.11 (0.98-1.26)	0.071
Sex (Male vs Female)	1.14 (0.85-1.53)	0.171
Race (Hispanic vs Non-Hispanic White)	0.89 (0.55-1.44)	0.218
Race1 (Non-Hispanic AAPI vs Non-Hispanic White)	0.48 (0.16-1.47)	0.274
Race2 (Non-Hispanic Black vs Non-Hispanic White)	1.23 (0.86-1.76)	0.224
Race3 (Other vs Non-Hispanic White)	1.26 (0.75-2.14)	0.338
H/o diabetes mellitus (yes vs none)	1.13 (0.84-1.52)	0.173
H/o cardiovascular risk factors (yes vs none)	1.33 (0.95-1.86)	0.229
H/o atrial fibrillation (yes vs none)	1.19 (0.78-1.82)	0.257
Cancer status (Active, stable/responding vs Remission/NED)	1.02 (0.68-1.54)	0.213
Cancer status2 (Active, progressing vs Remission/NED)	0.93 (0.56-1.54)	0.239
Cancer status3 (Unknown vs Remission/NED)	1.99 (1.33-2.99)	0.412
Anticoagulation at baseline (yes vs. no)	1.14 (0.70-1.85)	0.281
Dose of anticoagulation (therapeutic vs. prophylactic vs. none)	0.82 (0.46-1.46)	0.242
Baseline ASA (low dose vs none)	1.52 (1.11-2.09)	0.247
Baseline ASA (high dose vs none)	1.25 (0.47-3.28)	0.616
Baseline antiplatelet (yes vs none)	1.59 (1.02-2.48)	0.360
**Severity: Moderate (exposed)**	0.68 (0.28-1.60)	0.297
**Severity: Severe (exposed)**	0.62 (0.23-1.67)	0.313

© 2023 Gulati S et al. *JAMA Oncology.*

eTable 11
A) 30 day (column 1) and 90 day (column 2) follow-up outcome. Results of regression analysis for **VTE outcome**, each anti-cancer therapy exposure within 3 months vs not receiving any systemic treatments within 3 months, with adjustment for covariates (N=4688). This analysis excluded ATE cases.

Characteristics	Multivariable aRR (95% CI) at 30 days follow-up	Multivariable aRR (95% CI) at 90 days follow-up
**Exposure to endocrine therapy in past 3 months vs. no exposure**	1.08 (0.73-1.60)	1.12 (0.78-1.62)
**Exposure to VEGFi/TKI in past 3 months vs. no exposure**	1.33 (0.92-1.93)	1.22 (0.85-1.75)
**Exposure to IMIDs in past 3 months vs. no exposure**	1.16 (0.60-2.25)	1.18 (0.63-2.20)
**Exposure to ICIs in past 3 months vs. no exposure**	1.42 (0.96-2.11)	1.38 (0.95-2.00)
**Exposure to chemotherapy in past 3 months vs. no exposure**	1.35 (1.04-1.76)	1.30 (1.01-1.66)
**Age** (per 10 years increase)	1.02 (0.94-1.11)	1.03 (0.95-1.12)
**Sex** (ref = Female)		
Male	1.07 (0.86-1.32)	1.01 (0.83-1.24)
**Race** (ref = Non-Hispanic White)		
Hispanic	0.79 (0.56-1.11)	0.80 (0.58-1.11)
Non-Hispanic AAPI	0.61 (0.30-1.28)	0.64 (0.32-1.27)
Non-Hispanic Black	1.27 (0.98-1.64)	1.18 (0.92-1.51)
Other	0.97 (0.63-1.51)	1.00 (0.67-1.50)
**BMI $\geq$ 30** (ref = 0)		
1	1.06 (0.85-1.33)	1.10 (0.88-1.36)
**ECOG performance status** (ref = 0)		
1	0.74 (0.55-0.98)	0.72 (0.55-0.95)
2+	0.67 (0.48-0.93)	0.63 (0.47-0.86)
Unknown	0.75 (0.56-1.01)	0.71 (0.53-0.95)
**Cancer status** (ref = Remission/NED)		
Active, stable/responding	0.95 (0.68-1.32)	1.03 (0.75-1.41)
Active, progressing	1.29 (0.89-1.87)	1.45 (1.02-2.06)
Unknown	1.54 (1.07-2.22)	1.66 (1.17-2.34)
**Cancer metastatic** (ref = No)		
Yes	0.96 (0.71-1.29)	0.94 (0.71-1.24)
**History of baseline VTE** (ref = No)		
Yes	2.96 (2.24-3.90)	2.81 (2.15-3.66)
**High risk for VTE per site** (ref = Low/Other)		
High/Very high	1.22 (0.97-1.53)	1.26 (1.01-1.56)
**On anticoagulation at baseline** (ref = No)		
Prophylactic	0.92 (0.64-1.33)	0.90 (0.63-1.28)
Therapeutic	0.98 (0.71-1.35)	0.99 (0.73-1.35)
**On baseline aspirin** (ref = No)		
Low dose	0.74 (0.56-0.98)	0.75 (0.57-0.98)
High dose	0.43 (0.11-1.72)	0.39 (0.10-1.54)
**On baseline anti-platelet agent** (ref = No)		
Yes	0.58 (0.29-1.16)	0.60 (0.31-1.15)

© 2023 Gulati S et al. *JAMA Oncology.*

B) 30 day (column 1) and 90 day (column 2) follow-up outcome. Results of regression analysis for **ATE outcome**, each anti-cancer therapy exposure within 3 months vs not receiving any systemic treatments within 3 months, with adjustment for covariates (N=4688). This analysis excluded VTE cases.

Characteristics	Multivariable aRR (95% CI) at 30 days follow-up	Multivariable aRR (95% CI) at 90 days follow-up
**Exposure to endocrine therapy in past 3 months vs. no exposure**	1.12 (0.66-1.90)	1.11 (0.66-1.85)
**Exposure to VEGFi/TKI in past 3 months vs. no exposure**	0.68 (0.27-1.68)	0.63 (0.25-1.55)
**Exposure to IMIDs in past 3 months vs. no exposure**	0.98 (0.37-2.60)	0.91 (0.35-2.39)
**Exposure to ICIs in past 3 months vs. no exposure**	0.30 (0.07-1.21)	0.28 (0.07-1.14)
**Exposure to chemotherapy in past 3 months vs. no exposure**	0.97 (0.60-1.57)	0.93 (0.58-1.50)
**Age**, per 10 years increase	1.11 (0.96-1.27)	1.09 (0.95-1.25)
**Sex** (ref = Female)		
Male	1.21 (0.87-1.68)	1.23 (0.89-1.68)
**Race** (ref = Non-Hispanic White)		
Hispanic	1.04 (0.63-1.73)	0.96 (0.58-1.59)
Non-Hispanic AAPI	0.58 (0.19-1.78)	0.54 (0.18-1.66)
Non-Hispanic Black	1.16 (0.77-1.74)	1.21 (0.82-1.79)
Other	1.17 (0.64-2.16)	1.39 (0.81-2.42)
**History of diabetes mellitus** (ref = No)		
1	1.12 (0.80-1.56)	1.19 (0.86-1.64)
**History of cardiovascular risk factors** (ref = No)		
1	1.42 (0.97-2.07)	1.40 (0.97-2.01)
**History of atrial fibrillation** (ref = No)		
1	1.31 (0.83-2.07)	1.26 (0.81-1.97)
**Cancer status** (ref = Remission/NED)		
Active, stable/responding	0.91 (0.57-1.44)	0.94 (0.60-1.45)
Active, progressing	1.06 (0.62-1.81)	0.99 (0.59-1.68)
Unknown	2.07 (1.32-3.25)	2.06 (1.34-3.17)
**On baseline anticoagulation** (ref = No)		
Prophylactic	1.11 (0.65-1.89)	1.12 (0.67-1.88)
Therapeutic	0.84 (0.46-1.52)	0.80 (0.44-1.47)
**On baseline aspirin** (ref = No)		
Low dose	1.55 (1.08-2.22)	1.48 (1.04-2.09)
High dose	1.04 (0.33-3.23)	1.25 (0.47-3.34)
**On baseline anti-platelet agents** (ref = No)		
Yes	1.79 (1.11-2.88)	1.66 (1.03-2.65)

© 2023 Gulati S et al. *JAMA Oncology.*

eFigure 1. Consort diagram

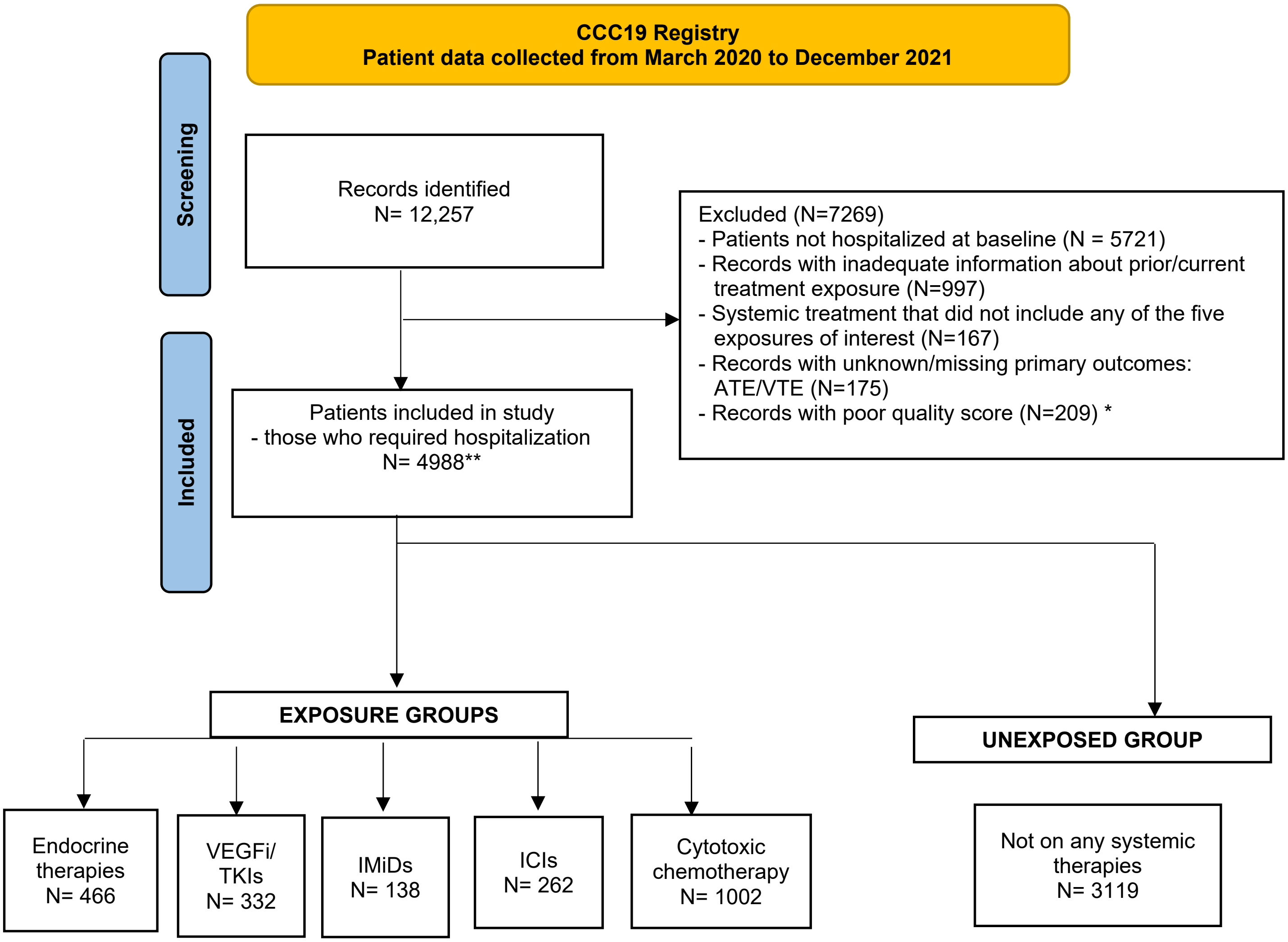

*Quality score >4 as previously defined were excluded 14
** Total number of patients in each of the treatment groups were not mutually exclusive, which is why they do not add up to N= 4988.
Abbreviations: CCC19, COVID-19 and Cancer Consortium; N, number; ATE, arterial thromboembolism; VTE, venous thromboembolism; IMids, immunomodulatory drugs; ICIs, immune checkpoint inhibitors

© 2023 Gulati S et al. *JAMA Oncology.*

eFigure 2. Combined VTE/ATE forest plot for the estimates of log odds ratios with the 95% confidence intervals for exposure to the five treatments of interest within 3 months vs. no exposure within 3 months

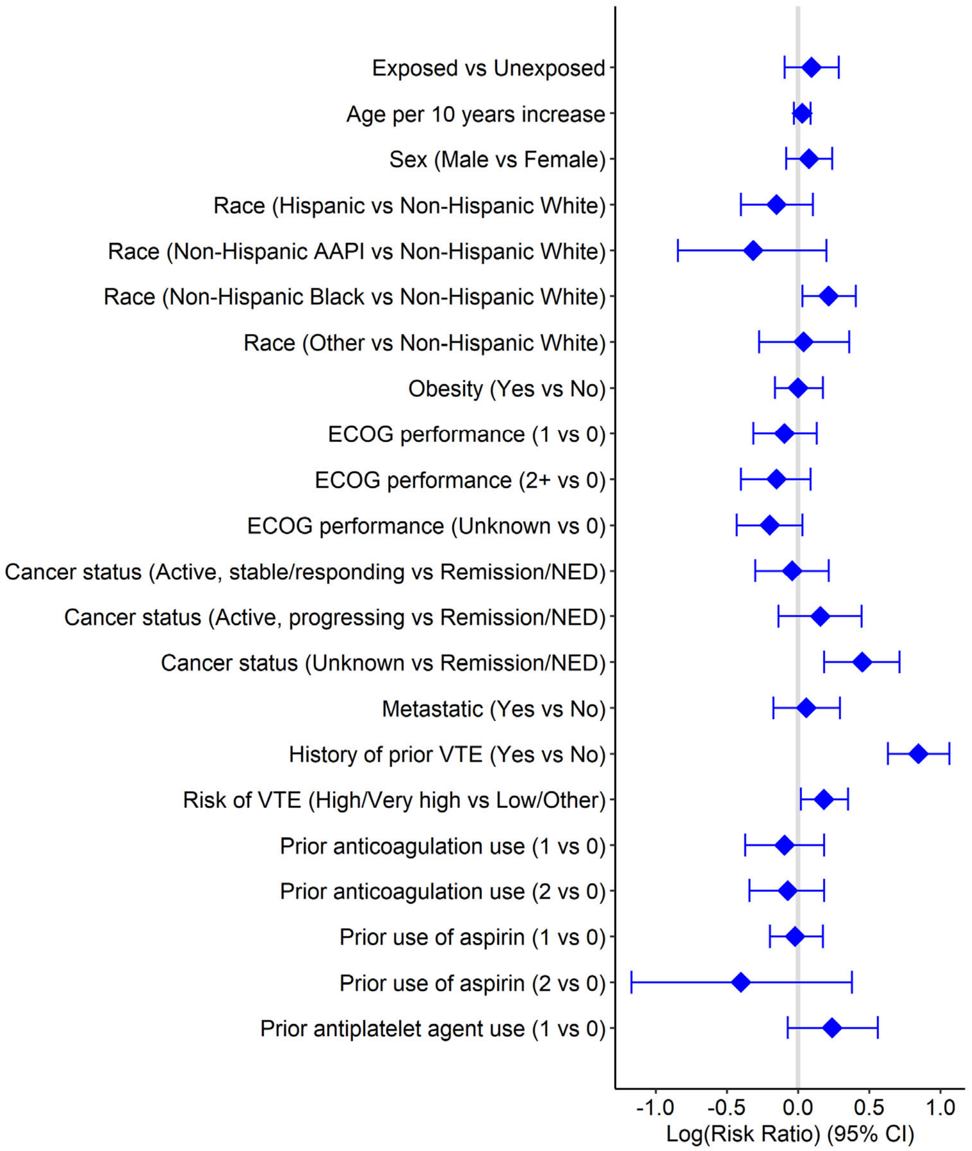

© 2023 Gulati S et al. *JAMA Oncology.*

eFigure 3. Forest plots for outcome of death after a thromboembolic event. Forest plots for the estimates of log risk ratios with the 95% confidence intervals for those on TOIs in the three months before COVID-19 diagnosis versus those not on systemic therapies in these three months.

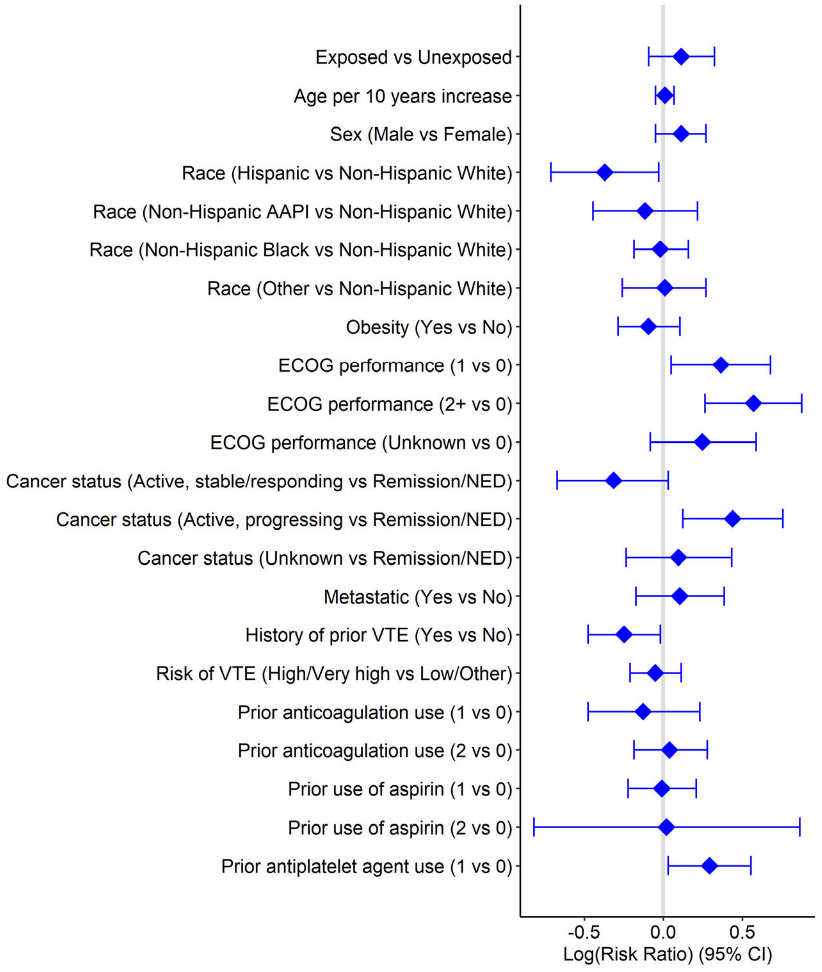

© 2023 Gulati S et al. *JAMA Oncology.*

eFigure 4. Forest plots for the estimates of log risk ratios with the 95% confidence intervals for patients exposed to systemic therapies of interest pooled together within 3 months vs. unexposed within 3 months

A) VTE

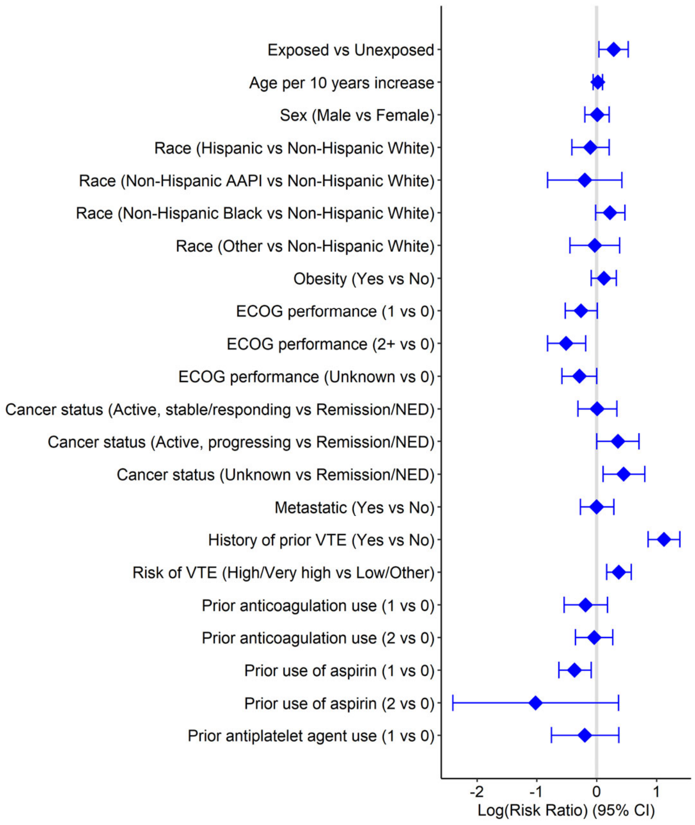

B) ATE

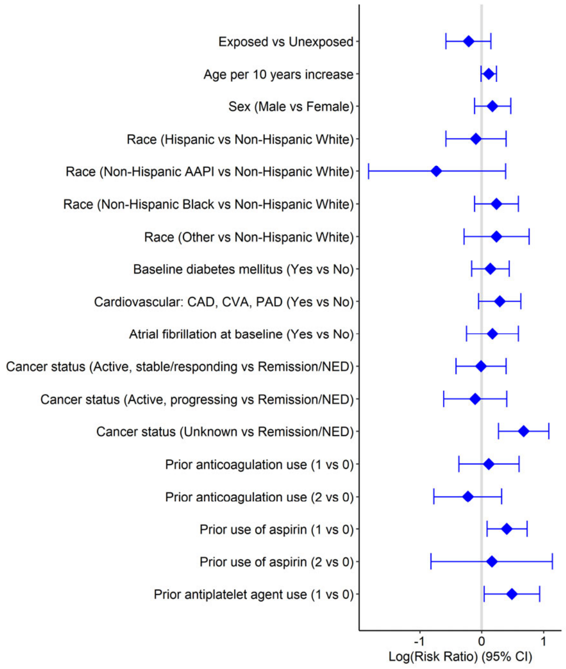

© 2023 Gulati S et al. *JAMA Oncology.*
