# Supplementary material for: Systemic Anticancer Therapy and Thromboembolic Outcomes in Hospitalized Patients With Cancer and COVID-19
Source: JAMA Oncol. 2023 Aug 17;9(10):1390–400. doi: 10.1001/jamaoncol.2023.2934 (PMC10436185; doi:10.1001/jamaoncol.2023.2934)
Supplement: Supplement 2. — COVID-19 and Cancer Consortium Nonauthor Collaborators [file jamaoncol-e232934-s002.pdf]

Supplemental Online Content: Nonauthor Collaborators
*First name, last name, and suffix (if applicable) are required and will appear in PubMed.

*Group Name(s): COVID-19 and Cancer Consortium							
***First Name and Middle Initial(s)**	***Last Name**	***Suffix (eg, Jr, III)**	**Academic Degrees**	**Institution**	**Location (city, state/province, country)**	**Role or Contribution, eg, chair, principal investigator**	**Group (if more than 1 Group listed in the byline) and/or Subgroup (eg, Steering Committee)**
Rikin	Gandhi		MS	Albert Einstein College of Medicine, Montefiore Medical Center	Bronx, New York, USA	Co-investigator	COVID-19 and Cancer Consortium
Benjamin A.	Gartrell		MD	Albert Einstein College of Medicine, Montefiore Medical Center	Bronx, New York, USA	Co-investigator	COVID-19 and Cancer Consortium
Sanjay	Goel		MBBS	Albert Einstein College of Medicine, Montefiore Medical Center	Bronx, New York, USA	Co-investigator	COVID-19 and Cancer Consortium
Balazs	Halmos		MD	Albert Einstein College of Medicine, Montefiore Medical Center	Bronx, New York, USA	Site PI	COVID-19 and Cancer Consortium
Della F.	Makower		MD	Albert Einstein College of Medicine, Montefiore Medical Center	Bronx, New York, USA	Co-investigator	COVID-19 and Cancer Consortium
Darciann	O' Sullivan		RN, MSN-ED, OCN	Albert Einstein College of Medicine, Montefiore Medical Center	Bronx, New York, USA	Co-investigator	COVID-19 and Cancer Consortium
Nitin	Ohri		MD	Albert Einstein College of Medicine, Montefiore Medical Center	Bronx, New York, USA	Co-investigator	COVID-19 and Cancer Consortium
R. Alejandro	Sica		MD	Albert Einstein College of Medicine, Montefiore Medical Center	Bronx, New York, USA	Co-investigator	COVID-19 and Cancer Consortium
Amit K.	Verma		MBBS	Albert Einstein College of Medicine, Montefiore Medical Center	Bronx, New York, USA	Site PI	COVID-19 and Cancer Consortium

Supplemental Online Content: Nonauthor Collaborators
*First name, last name, and suffix (if applicable) are required and will appear in PubMed.

*First Name and Middle Initial(s)	*Last Name	*Suffix (eg, Jr, III)	Academic Degrees	Institution	Location (city, state/province, country)	Role or Contribution, eg, chair, principal investigator	Group (if more than 1 Group listed in the byline) and/or Subgroup (eg, Steering Committee)
Omar	Butt		MD, PhD	Alvin J. Siteman Cancer Center at Washington University School of Medicine and Barnes-Jewish Hospital	St. Louis, Missouri, USA	Co-investigator	COVID-19 and Cancer Consortium
Mark A.	Fiala		MSW	Alvin J. Siteman Cancer Center at Washington University School of Medicine and Barnes-Jewish Hospital	St. Louis, Missouri, USA	Co-investigator	COVID-19 and Cancer Consortium
Jeffrey P.	Henderson		MD, PhD	Alvin J. Siteman Cancer Center at Washington University School of Medicine and Barnes-Jewish Hospital	St. Louis, Missouri, USA	Co-investigator	COVID-19 and Cancer Consortium
Ryan S.	Monahan		MBA	Alvin J. Siteman Cancer Center at Washington University School of Medicine and Barnes-Jewish Hospital	St. Louis, Missouri, USA	Co-investigator	COVID-19 and Cancer Consortium
Keith E.	Stockerl-Goldstein		MD	Alvin J. Siteman Cancer Center at Washington University School of Medicine and Barnes-Jewish Hospital	St. Louis, Missouri, USA	Site PI	COVID-19 and Cancer Consortium
Alice Y.	Zhou		MD, PhD	Alvin J. Siteman Cancer Center at Washington University School of Medicine and Barnes-Jewish Hospital	St. Louis, Missouri, USA	Co-investigator	COVID-19 and Cancer Consortium
Jacob D.	Bitran		MD	Aurora Cancer Care, Advocate Aurora Health	Milwaukee, Wisconsin, USA	Co-investigator	COVID-19 and Cancer Consortium
Sigrun	Hallmeyer		MD	Aurora Cancer Care, Advocate Aurora Health	Milwaukee, Wisconsin, USA	Site PI	COVID-19 and Cancer Consortium
Daniel	Mundt		MD	Aurora Cancer Care, Advocate Aurora Health	Milwaukee, Wisconsin, USA	Co-investigator	COVID-19 and Cancer Consortium

*First name, last name, and suffix (if applicable) are required and will appear in PubMed.

*First Name and Middle Initial(s)	*Last Name	*Suffix (eg, Jr, III)	Academic Degrees	Institution	Location (city, state/province, country)	Role or Contribution, eg, chair, principal investigator	Group (if more than 1 Group listed in the byline) and/or Subgroup (eg, Steering Committee)
Sasirekha	Pandravada		DO	Aurora Cancer Care, Advocate Aurora Health	Milwaukee, Wisconsin, USA	Co-investigator	COVID-19 and Cancer Consortium
Philip V.	Papaioannou		BS	Aurora Cancer Care, Advocate Aurora Health	Milwaukee, Wisconsin, USA	Co-investigator	COVID-19 and Cancer Consortium
Mauli	Patel		DO	Aurora Cancer Care, Advocate Aurora Health	Milwaukee, Wisconsin, USA	Co-investigator	COVID-19 and Cancer Consortium
Mitrianna	Streckfuss		MPH, CCRP	Aurora Cancer Care, Advocate Aurora Health	Milwaukee, Wisconsin, USA	Co-investigator	COVID-19 and Cancer Consortium
Eyob	Tadesse		MD	Aurora Cancer Care, Advocate Aurora Health	Milwaukee, Wisconsin, USA	Co-investigator	COVID-19 and Cancer Consortium
Michael A.	Thompson		MD, PhD, FASCO	Aurora Cancer Care, Advocate Aurora Health	Milwaukee, Wisconsin, USA	Co-investigator; Steering Committee member	COVID-19 and Cancer Consortium
Philip E.	Lammers		MD, MSCI	Baptist Cancer Center	Memphis, Tennessee, USA	Site PI	COVID-19 and Cancer Consortium
Jonathan M.	Loree		MD, MS, FRCPC	BC Cancer	Vancouver, British Columbia, Canada	Site PI	COVID-19 and Cancer Consortium
Irene S.	Yu		MD, FRCPC	BC Cancer	Vancouver, British Columbia, Canada	Co-investigator	COVID-19 and Cancer Consortium
Poorva	Bindal		MD	Beth Israel Deaconess Medical Center	Boston, Massachusetts, USA	Co-investigator	COVID-19 and Cancer Consortium
Barbara	Lam		MD	Beth Israel Deaconess Medical Center	Boston, Massachusetts, USA	Co-investigator	COVID-19 and Cancer Consortium
Mary Linton B.	Peters		MD, MS, FACP	Beth Israel Deaconess Medical Center	Boston, Massachusetts, USA	Site PI	COVID-19 and Cancer Consortium
Andrew J.	Piper-Vallillo		MD	Beth Israel Deaconess Medical Center	Boston, Massachusetts, USA	Co-investigator	COVID-19 and Cancer Consortium
Panos	Arvanitis		MS	Brown University and Lifespan Cancer Institute	Providence, Rhode Island, USA	Co-investigator	COVID-19 and Cancer Consortium
Pamela C.	Egan		MD	Brown University and Lifespan Cancer Institute	Providence, Rhode Island, USA	Co-investigator	COVID-19 and Cancer Consortium

Supplemental Online Content: Nonauthor Collaborators
*First name, last name, and suffix (if applicable) are required and will appear in PubMed.

*First Name and Middle Initial(s)	*Last Name	*Suffix (eg, Jr, III)	Academic Degrees	Institution	Location (city, state/province, country)	Role or Contribution, eg, chair, principal investigator	Group (if more than 1 Group listed in the byline) and/or Subgroup (eg, Steering Committee)
Dimitrios	Farmakiotis		MD, FACP, FIDSA	Brown University and Lifespan Cancer Institute	Providence, Rhode Island, USA	Co-investigator; Steering Committee member	COVID-19 and Cancer Consortium
Adam J.	Olszewski		MD	Brown University and Lifespan Cancer Institute	Providence, Rhode Island, USA	Co-investigator	COVID-19 and Cancer Consortium
Kendra	Vieira		BS	Brown University and Lifespan Cancer Institute	Providence, Rhode Island, USA	Co-investigator	COVID-19 and Cancer Consortium
Anne H.	Angevine		MD	Carl & Dorothy Bennett Cancer Center at Stamford Hospital	Stamford, Connecticut, USA	Co-investigator	COVID-19 and Cancer Consortium
Michael H.	Bar		MD, FACP	Carl & Dorothy Bennett Cancer Center at Stamford Hospital	Stamford, Connecticut, USA	Co-investigator	COVID-19 and Cancer Consortium
Salvatore A.	Del Prete		MD	Carl & Dorothy Bennett Cancer Center at Stamford Hospital	Stamford, Connecticut, USA	Site PI	COVID-19 and Cancer Consortium
Maryann Z.	Fiebach		MPH	Carl & Dorothy Bennett Cancer Center at Stamford Hospital	Stamford, Connecticut, USA	Co-investigator	COVID-19 and Cancer Consortium
Anthony P.	Gulati		MD	Carl & Dorothy Bennett Cancer Center at Stamford Hospital	Stamford, Connecticut, USA	Co-investigator	COVID-19 and Cancer Consortium
Edward	Hatton		RN, BSN	Carl & Dorothy Bennett Cancer Center at Stamford Hospital	Stamford, Connecticut, USA	Co-investigator	COVID-19 and Cancer Consortium
Kaly	Houston			Carl & Dorothy Bennett Cancer Center at Stamford Hospital	Stamford, Connecticut, USA	Co-investigator	COVID-19 and Cancer Consortium
Suzanne J.	Rose		MS, PhD, CCRC, FACRP	Carl & Dorothy Bennett Cancer Center at Stamford Hospital	Stamford, Connecticut, USA	Co-investigator	COVID-19 and Cancer Consortium
K. M.	Steve Lo		MD	Carl & Dorothy Bennett Cancer Center at Stamford Hospital	Stamford, Connecticut, USA	Co-investigator	COVID-19 and Cancer Consortium
Jamie	Stratton		MD	Carl & Dorothy Bennett Cancer Center at Stamford Hospital	Stamford, Connecticut, USA	Co-investigator	COVID-19 and Cancer Consortium
Paul L.	Weinstein		MD	Carl & Dorothy Bennett Cancer Center at Stamford Hospital	Stamford, Connecticut, USA	Co-investigator	COVID-19 and Cancer Consortium

Supplemental Online Content: Nonauthor Collaborators
*First name, last name, and suffix (if applicable) are required and will appear in PubMed.

*First Name and Middle Initial(s)	*Last Name	*Suffix (eg, Jr, III)	Academic Degrees	Institution	Location (city, state/province, country)	Role or Contribution, eg, chair, principal investigator	Group (if more than 1 Group listed in the byline) and/or Subgroup (eg, Steering Committee)
Jorge A.	Garcia		MD, FACP	Case Comprehensive Cancer Center at Case Western Reserve University/University Hospitals	Cleveland, Ohio, USA	Site PI	COVID-19 and Cancer Consortium
Bertrand	Routy		MD, PhD	Centre Hospitalier de l'Université de Montréal (CHUM)	Montreal, Quebec, Canada	Site PI	COVID-19 and Cancer Consortium
Irma	Hoyo-Ulloa		MD	Centro Médico ABC	Mexico City, Mexico City, Mexico	Site PI	COVID-19 and Cancer Consortium
Shilpa	Gupta		MD	Cleveland Clinic	Cleveland, Ohio, USA	Site PI	COVID-19 and Cancer Consortium
Amanda	Nizam		MD	Cleveland Clinic	Cleveland, Ohio, USA	Co-investigator	COVID-19 and Cancer Consortium
Nathan A.	Pennell		MD, PhD, FASCO	Cleveland Clinic	Cleveland, Ohio, USA	Site PI	COVID-19 and Cancer Consortium
Nima	Sharifi		MD	Cleveland Clinic	Cleveland, Ohio, USA	Co-investigator	COVID-19 and Cancer Consortium
Claire	Hoppenot		MD	Dan L Duncan Comprehensive Cancer Center at Baylor College of Medicine	Houston, Texas, USA	Site PI	COVID-19 and Cancer Consortium
Ang	Li		MD, MS	Dan L Duncan Comprehensive Cancer Center at Baylor College of Medicine	Houston, Texas, USA	Site PI	COVID-19 and Cancer Consortium
Danielle S.	Bitterman		MD	Dana-Farber Cancer Institute	Boston, Massachusetts, USA	Co-investigator	COVID-19 and Cancer Consortium
Toni K.	Choueiri		MD	Dana-Farber Cancer Institute	Boston, Massachusetts, USA	Site PI; Steering Committee member	COVID-19 and Cancer Consortium
Jean M.	Connors		MD	Dana-Farber Cancer Institute	Boston, Massachusetts, USA	Co-investigator	COVID-19 and Cancer Consortium
George D.	Demetri		MD, FASCO	Dana-Farber Cancer Institute	Boston, Massachusetts, USA	Co-investigator	COVID-19 and Cancer Consortium
Talal	El Zarif		MD	Dana-Farber Cancer Institute	Boston, Massachusetts, USA	Co-investigator	COVID-19 and Cancer Consortium

*First name, last name, and suffix (if applicable) are required and will appear in PubMed.

*First Name and Middle Initial(s)	*Last Name	*Suffix (eg, Jr, III)	Academic Degrees	Institution	Location (city, state/province, country)	Role or Contribution, eg, chair, principal investigator	Group (if more than 1 Group listed in the byline) and/or Subgroup (eg, Steering Committee)
Narjust	Florez		MD	Dana-Farber Cancer Institute	Boston, Massachusetts, USA	Co-investigator; Steering Committee Member	COVID-19 and Cancer Consortium
Dory A.	Freeman		BS	Dana-Farber Cancer Institute	Boston, Massachusetts, USA	Co-investigator	COVID-19 and Cancer Consortium
Antonio	Giordano		MD, PhD	Dana-Farber Cancer Institute	Boston, Massachusetts, USA	Co-investigator	COVID-19 and Cancer Consortium
Alicia K.	Morgans		MD, MPH	Dana-Farber Cancer Institute	Boston, Massachusetts, USA	Co-investigator	COVID-19 and Cancer Consortium
Anju	Nohria		MD	Dana-Farber Cancer Institute	Boston, Massachusetts, USA	Co-investigator	COVID-19 and Cancer Consortium
Sara M.	Tolaney		MD, MPH	Dana-Farber Cancer Institute	Boston, Massachusetts, USA	Co-investigator	COVID-19 and Cancer Consortium
Eliezer M.	Van Allen		MD	Dana-Farber Cancer Institute	Boston, Massachusetts, USA	Co-investigator	COVID-19 and Cancer Consortium
Wenxin Vincent	Xu		MD	Dana-Farber Cancer Institute	Boston, Massachusetts, USA	Co-investigator	COVID-19 and Cancer Consortium
Susan	Halabi		PhD, FASCO	Duke Cancer Institute at Duke University Medical Center	Durham, North Carolina, USA	Site PI	COVID-19 and Cancer Consortium
Tian	Zhang		MD, MHS	Duke Cancer Institute at Duke University Medical Center	Durham, North Carolina, USA	Co-investigator	COVID-19 and Cancer Consortium
Hannah	Dzimitrowicz		MD	Duke Cancer Institute at Duke University Medical Center; UT Southwestern	Durham, North Carolina, USA	Co-investigator	COVID-19 and Cancer Consortium
John C.	Leighton		MD, FACP	Einstein Healthcare Network	Philadelphia, Pennsylvania, USA	Site PI	COVID-19 and Cancer Consortium
Jerome J.	Graber		MD, MPH	Fred Hutchinson Cancer Research Center/University of Washington/Seattle Cancer Care Alliance	Seattle, Washington, USA	Co-investigator	COVID-19 and Cancer Consortium

Supplemental Online Content: Nonauthor Collaborators
*First name, last name, and suffix (if applicable) are required and will appear in PubMed.

*First Name and Middle Initial(s)	*Last Name	*Suffix (eg, Jr, III)	Academic Degrees	Institution	Location (city, state/province, country)	Role or Contribution, eg, chair, principal investigator	Group (if more than 1 Group listed in the byline) and/or Subgroup (eg, Steering Committee)
Jessica E.	Hawley		MD	Fred Hutchinson Cancer Research Center/University of Washington/Seattle Cancer Care Alliance	Seattle, Washington, USA	Co-investigator	COVID-19 and Cancer Consortium
Elizabeth T.	Loggers		MD, PhD	Fred Hutchinson Cancer Research Center/University of Washington/Seattle Cancer Care Alliance	Seattle, Washington, USA	Co-investigator	COVID-19 and Cancer Consortium
Gary H.	Lyman		MD, MPH, FASCO, FACP, FRCP	Fred Hutchinson Cancer Research Center/University of Washington/Seattle Cancer Care Alliance	Seattle, Washington, USA	Site PI	COVID-19 and Cancer Consortium
Ryan C.	Lynch		MD	Fred Hutchinson Cancer Research Center/University of Washington/Seattle Cancer Care Alliance	Seattle, Washington, USA	Co-investigator	COVID-19 and Cancer Consortium
Andrew J.	Portuguese		MD	Fred Hutchinson Cancer Research Center/University of Washington/Seattle Cancer Care Alliance	Seattle, Washington, USA	Co-investigator	COVID-19 and Cancer Consortium
Michael T.	Schweizer		MD	Fred Hutchinson Cancer Research Center/University of Washington/Seattle Cancer Care Alliance	Seattle, Washington, USA	Co-investigator	COVID-19 and Cancer Consortium
Christopher T.	Su		MD	Fred Hutchinson Cancer Research Center/University of Washington/Seattle Cancer Care Alliance	Seattle, Washington, USA	Co-investigator	COVID-19 and Cancer Consortium

*First name, last name, and suffix (if applicable) are required and will appear in PubMed.

*First Name and Middle Initial(s)	*Last Name	*Suffix (eg, Jr, III)	Academic Degrees	Institution	Location (city, state/province, country)	Role or Contribution, eg, chair, principal investigator	Group (if more than 1 Group listed in the byline) and/or Subgroup (eg, Steering Committee)
Lisa	Tachiki		MD	Fred Hutchinson Cancer Research Center/University of Washington/Seattle Cancer Care Alliance	Seattle, Washington, USA	Co-investigator	COVID-19 and Cancer Consortium
Shaveta	Vinayak		MD, MS	Fred Hutchinson Cancer Research Center/University of Washington/Seattle Cancer Care Alliance	Seattle, Washington, USA	Co-investigator	COVID-19 and Cancer Consortium
Michael J.	Wagner		MD	Fred Hutchinson Cancer Research Center/University of Washington/Seattle Cancer Care Alliance	Seattle, Washington, USA	Co-investigator	COVID-19 and Cancer Consortium
Albert	Yeh		MD	Fred Hutchinson Cancer Research Center/University of Washington/Seattle Cancer Care Alliance	Seattle, Washington, USA	Co-investigator	COVID-19 and Cancer Consortium
Yvonne	Dansoa		DO	Geisinger Health System	Danville, Pennsylvania, USA	Co-investigator	COVID-19 and Cancer Consortium
Na Tosha N.	Gatson		MD, PhD, FAAN	Geisinger Health System	Danville, Pennsylvania, USA	Site PI	COVID-19 and Cancer Consortium
Mina	Makary		MD	Geisinger Health System	Danville, Pennsylvania, USA	Co-investigator	COVID-19 and Cancer Consortium
Jesse J.	Manikowski		MS	Geisinger Health System	Danville, Pennsylvania, USA	Co-investigator	COVID-19 and Cancer Consortium
Joseph	Vadakara		MD	Geisinger Health System	Danville, Pennsylvania, USA	Co-investigator	COVID-19 and Cancer Consortium
Kristena	Yossef		MD	Geisinger Health System	Danville, Pennsylvania, USA	Co-investigator	COVID-19 and Cancer Consortium
Jennifer	Beckerman		MD	George Washington University	Washington, Washington, USA	Co-investigator	COVID-19 and Cancer Consortium

Supplemental Online Content: Nonauthor Collaborators
*First name, last name, and suffix (if applicable) are required and will appear in PubMed.

*First Name and Middle Initial(s)	*Last Name	*Suffix (eg, Jr, III)	Academic Degrees	Institution	Location (city, state/province, country)	Role or Contribution, eg, chair, principal investigator	Group (if more than 1 Group listed in the byline) and/or Subgroup (eg, Steering Committee)
Sharad	Goyal		MD	George Washington University	Washington, Washington, USA	Site PI	COVID-19 and Cancer Consortium
Ian	Messing		MST	George Washington University	Washington, Washington, USA	Co-investigator	COVID-19 and Cancer Consortium
Destie	Provenzano		MS	George Washington University	Washington, Washington, USA	Co-investigator	COVID-19 and Cancer Consortium
Yuan James	Rao		MD	George Washington University	Washington, Washington, USA	Co-investigator	COVID-19 and Cancer Consortium
Lori J.	Rosenstein		MD	Gundersen Health System	La Crosse, Wisconsin, USA	Site PI	COVID-19 and Cancer Consortium
Dawn R.	Steffes			Gundersen Health System	La Crosse, Wisconsin, USA	Co-investigator	COVID-19 and Cancer Consortium
Jessica M.	Clement		MD	Hartford HealthCare Cancer Institute	Hartford, Connecticut, USA	Co-investigator	COVID-19 and Cancer Consortium
Jonathan A.	Cosin		MD	Hartford HealthCare Cancer Institute	Hartford, Connecticut, USA	Co-investigator	COVID-19 and Cancer Consortium
Ahmad	Daher		MD	Hartford HealthCare Cancer Institute	Hartford, Connecticut, USA	Co-investigator	COVID-19 and Cancer Consortium
Mark E.	Dailey		MD	Hartford HealthCare Cancer Institute	Hartford, Connecticut, USA	Co-investigator	COVID-19 and Cancer Consortium
Rawad	Elias		MD	Hartford HealthCare Cancer Institute	Hartford, Connecticut, USA	Co-investigator	COVID-19 and Cancer Consortium
Omar E.	Eton		MD	Hartford HealthCare Cancer Institute	Hartford, Connecticut, USA	Co-investigator	COVID-19 and Cancer Consortium
Wylie	Hosmer		MD	Hartford HealthCare Cancer Institute	Hartford, Connecticut, USA	Co-investigator	COVID-19 and Cancer Consortium
Emily	Hsu		MD	Hartford HealthCare Cancer Institute	Hartford, Connecticut, USA	Co-investigator	COVID-19 and Cancer Consortium
Asha	Jayaraj		MD	Hartford HealthCare Cancer Institute	Hartford, Connecticut, USA	Co-investigator	COVID-19 and Cancer Consortium

*First name, last name, and suffix (if applicable) are required and will appear in PubMed.

*First Name and Middle Initial(s)	*Last Name	*Suffix (eg, Jr, III)	Academic Degrees	Institution	Location (city, state/province, country)	Role or Contribution, eg, chair, principal investigator	Group (if more than 1 Group listed in the byline) and/or Subgroup (eg, Steering Committee)
Jeff	Mather		MS	Hartford HealthCare Cancer Institute	Hartford, Connecticut, USA	Co-investigator	COVID-19 and Cancer Consortium
Alvaro G.	Menendez		MD	Hartford HealthCare Cancer Institute	Hartford, Connecticut, USA	Co-investigator	COVID-19 and Cancer Consortium
Rajani	Nadkarni		MD	Hartford HealthCare Cancer Institute	Hartford, Connecticut, USA	Co-investigator	COVID-19 and Cancer Consortium
Oscar K.	Serrano		MD, MBA, FACS	Hartford HealthCare Cancer Institute	Hartford, Connecticut, USA	Co-investigator	COVID-19 and Cancer Consortium
Peter Paul	Yu		MD, FACP, FASCO	Hartford HealthCare Cancer Institute	Hartford, Connecticut, USA	Site PI	COVID-19 and Cancer Consortium
Shirish M.	Gadgeel		MD	Henry Ford Cancer Institute, Henry Ford Hospital	Detroit, Michigan, USA	Co-investigator	COVID-19 and Cancer Consortium
Sheela	Tejwani		MD	Henry Ford Cancer Institute, Henry Ford Hospital	Detroit, Michigan, USA	Co-investigator	COVID-19 and Cancer Consortium
Melissa K.	Accordino		MD, MS	Herbert Irving Comprehensive Cancer Center at Columbia University	New York, New York, USA	Site PI	COVID-19 and Cancer Consortium
Divaya	Bhutani		MD	Herbert Irving Comprehensive Cancer Center at Columbia University	New York, New York, USA	Co-investigator	COVID-19 and Cancer Consortium
Brianne E.	Bodin		BSN, RN, OCN	Herbert Irving Comprehensive Cancer Center at Columbia University	New York, New York, USA	Co-investigator	COVID-19 and Cancer Consortium
Dawn L.	Hershman		MD, MS, FASCO	Herbert Irving Comprehensive Cancer Center at Columbia University	New York, New York, USA	Co-investigator	COVID-19 and Cancer Consortium
Matthew	Ingham		MD	Herbert Irving Comprehensive Cancer Center at Columbia University	New York, New York, USA	Co-investigator	COVID-19 and Cancer Consortium

Supplemental Online Content: Nonauthor Collaborators
*First name, last name, and suffix (if applicable) are required and will appear in PubMed.

*First Name and Middle Initial(s)	*Last Name	*Suffix (eg, Jr, III)	Academic Degrees	Institution	Location (city, state/province, country)	Role or Contribution, eg, chair, principal investigator	Group (if more than 1 Group listed in the byline) and/or Subgroup (eg, Steering Committee)
Shaheer A.	Khan		DO	Herbert Irving Comprehensive Cancer Center at Columbia University	New York, New York, USA	Co-investigator	COVID-19 and Cancer Consortium
Cynthia	Masson			Herbert Irving Comprehensive Cancer Center at Columbia University	New York, New York, USA	Co-investigator	COVID-19 and Cancer Consortium
Gary K.	Schwartz		MD	Herbert Irving Comprehensive Cancer Center at Columbia University	New York, New York, USA	Co-investigator	COVID-19 and Cancer Consortium
Mariam	Alexander		MD, PhD	Hollings Cancer Center at the Medical University of South Carolina	Charleston, South Carolina, USA	Co-investigator	COVID-19 and Cancer Consortium
Sara	Matar		MD	Hollings Cancer Center at the Medical University of South Carolina	Charleston, South Carolina, USA	Co-investigator	COVID-19 and Cancer Consortium
Daniel Y.	Reuben		MD, MS	Hollings Cancer Center at the Medical University of South Carolina	Charleston, South Carolina, USA	Site PI	COVID-19 and Cancer Consortium
Eric H.	Bernicker		MD	Houston Methodist Cancer Center	Houston, Texas, USA	Site PI	COVID-19 and Cancer Consortium
John F.	Deeken		MD	Inova Schar Cancer Institute	Fairfax, Virginia, USA	Site PI	COVID-19 and Cancer Consortium
Kelly J.	Jeffords		CCRC, CCRP	Inova Schar Cancer Institute	Fairfax, Virginia, USA	Co-investigator	COVID-19 and Cancer Consortium
Danielle	Shafer		DO	Inova Schar Cancer Institute	Fairfax, Virginia, USA	Co-investigator	COVID-19 and Cancer Consortium
Ana I.	Cárdenas-Delgado		BS	Instituto Nacional de Cancerologia	Mexico City, Mexico City, Mexico	Co-investigator	COVID-19 and Cancer Consortium
Rogelio	Cuervo Campos		MD	Instituto Nacional de Cancerologia	Mexico City, Mexico City, Mexico	Co-investigator	COVID-19 and Cancer Consortium

*First name, last name, and suffix (if applicable) are required and will appear in PubMed.

*First Name and Middle Initial(s)	*Last Name	*Suffix (eg, Jr, III)	Academic Degrees	Institution	Location (city, state/province, country)	Role or Contribution, eg, chair, principal investigator	Group (if more than 1 Group listed in the byline) and/or Subgroup (eg, Steering Committee)
Daniel	De-la-Rosa-Martinez		MD	Instituto Nacional de Cancerologia	Mexico City, Mexico City, Mexico	Co-investigator	COVID-19 and Cancer Consortium
Ana	Ramirez		MD	Instituto Nacional de Cancerologia	Mexico City, Mexico City, Mexico	Co-investigator	COVID-19 and Cancer Consortium
Nadia Melissa	Valdez-Reyes		MD	Instituto Nacional de Cancerologia	Mexico City, Mexico City, Mexico	Co-investigator	COVID-19 and Cancer Consortium
David M.	Gill		MD	Intermountain Healthcare	Salt Lake City, Utah, USA	Co-investigator	COVID-19 and Cancer Consortium
Mark A.	Lewis		MD	Intermountain Healthcare	Salt Lake City, Utah, USA	Site PI	COVID-19 and Cancer Consortium
Clarke A.	Low		MD	Intermountain Healthcare	Salt Lake City, Utah, USA	Co-investigator	COVID-19 and Cancer Consortium
Terence D.	Rhodes		MD, PhD	Intermountain Healthcare	Salt Lake City, Utah, USA	Site PI	COVID-19 and Cancer Consortium
Michelle M.	Jones		RN	Kaiser Permanente Northwest	, Washington, USA	Co-investigator	COVID-19 and Cancer Consortium
Abdul-Hai	Mansoor		MD	Kaiser Permanente Northwest	, Washington, USA	Co-investigator	COVID-19 and Cancer Consortium
Sandeep H.	Mashru		MD	Kaiser Permanente Northwest	, Washington, USA	Site PI	COVID-19 and Cancer Consortium
Mica A.	Werner		MSM	Kaiser Permanente Northwest	, Washington, USA	Co-investigator	COVID-19 and Cancer Consortium
Aaron M.	Cohen		MD, MS	Knight Cancer Institute at Oregon Health and Science University	Portland, Oregon, USA	Co-investigator	COVID-19 and Cancer Consortium
Brandon M.	Hayes-Lattin		MD, FACP	Knight Cancer Institute at Oregon Health and Science University	Portland, Oregon, USA	Site PI	COVID-19 and Cancer Consortium
Shannon	McWeeney		PhD	Knight Cancer Institute at Oregon Health and Science University	Portland, Oregon, USA	Co-investigator	COVID-19 and Cancer Consortium
Eneida R.	Nemecek		MD, MS, MBA	Knight Cancer Institute at Oregon Health and Science University	Portland, Oregon, USA	Co-investigator	COVID-19 and Cancer Consortium

Supplemental Online Content: Nonauthor Collaborators
*First name, last name, and suffix (if applicable) are required and will appear in PubMed.

*First Name and Middle Initial(s)	*Last Name	*Suffix (eg, Jr, III)	Academic Degrees	Institution	Location (city, state/province, country)	Role or Contribution, eg, chair, principal investigator	Group (if more than 1 Group listed in the byline) and/or Subgroup (eg, Steering Committee)
Staci P.	Williamson		BS	Knight Cancer Institute at Oregon Health and Science University	Portland, Oregon, USA	Co-investigator	COVID-19 and Cancer Consortium
Grant C.	Lewis		MD	Lewis Cancer & Research Pavilion @ St. Joseph's/Candler	Savannah, Georgia, USA	Site PI	COVID-19 and Cancer Consortium
Stephanie J.	Smith		RN, MSN, OCN	Lewis Cancer & Research Pavilion @ St. Joseph's/Candler	Savannah, Georgia, USA	Co-investigator	COVID-19 and Cancer Consortium
Mojtaba	Akhtari		MD	Loma Linda University Cancer Center	Loma Linda, California, USA	Co-investigator	COVID-19 and Cancer Consortium
Dan R.	Castillo		MD	Loma Linda University Cancer Center	Loma Linda, California, USA	Co-investigator	COVID-19 and Cancer Consortium
Kimberly	Cortez		BS	Loma Linda University Cancer Center	Loma Linda, California, USA	Co-investigator	COVID-19 and Cancer Consortium
Kyu	Park		BA	Loma Linda University Cancer Center	Loma Linda, California, USA	Co-investigator	COVID-19 and Cancer Consortium
Mark E.	Reeves		MD, PhD	Loma Linda University Cancer Center	Loma Linda, California, USA	Co-investigator	COVID-19 and Cancer Consortium
Stephanie	Berg		DO	Loyola University Medical Center	Maywood, Illinois, USA	Site PI	COVID-19 and Cancer Consortium
Timothy E.	O'Connor		MD	Loyola University Medical Center	Maywood, Illinois, USA	Co-investigator	COVID-19 and Cancer Consortium
Jessica	Altman		MD	Lurie Cancer Center at Northwestern University	Chicago, Illinois, USA	Co-investigator	COVID-19 and Cancer Consortium
Michael	Gurley		BA	Lurie Cancer Center at Northwestern University	Chicago, Illinois, USA	Co-investigator	COVID-19 and Cancer Consortium
Mary F.	Mulcahy		MD	Lurie Cancer Center at Northwestern University	Chicago, Illinois, USA	Co-investigator	COVID-19 and Cancer Consortium
Firas H.	Wehbe		MD, PhD	Lurie Cancer Center at Northwestern University	Chicago, Illinois, USA	Site PI	COVID-19 and Cancer Consortium
Eric B.	Durbin		DrPH, MS	Markey Cancer Center at the University of Kentucky	Lexington, Kentucky, USA	Site PI	COVID-19 and Cancer Consortium

*First name, last name, and suffix (if applicable) are required and will appear in PubMed.

*First Name and Middle Initial(s)	*Last Name	*Suffix (eg, Jr, III)	Academic Degrees	Institution	Location (city, state/province, country)	Role or Contribution, eg, chair, principal investigator	Group (if more than 1 Group listed in the byline) and/or Subgroup (eg, Steering Committee)
Amit A.	Kulkarni		MD	Masonic Cancer Center at the University of Minnesota	Minneapolis, Minnesota, USA	Site PI	COVID-19 and Cancer Consortium
Heather H.	Nelson		PhD, MPH	Masonic Cancer Center at the University of Minnesota	Minneapolis, Minnesota, USA	Co-investigator	COVID-19 and Cancer Consortium
Vidhyalakshmi	Ramesh			Masonic Cancer Center at the University of Minnesota	Miami, Florida, USA	Co-investigator	COVID-19 and Cancer Consortium
Zohar	Sachs		MD, PhD	Masonic Cancer Center at the University of Minnesota	Minneapolis, Minnesota, USA	Co-investigator	COVID-19 and Cancer Consortium
Grace	Wilson		BA	Masonic Cancer Center at the University of Minnesota	Miami, Florida, USA	Co-investigator	COVID-19 and Cancer Consortium
Aditya	Bardia		MD	Massachusetts General Hospital Cancer Center	Boston, Massachusetts, USA	Co-investigator	COVID-19 and Cancer Consortium
Genevieve	Boland		MD, PhD, FACS	Massachusetts General Hospital Cancer Center	Boston, Massachusetts, USA	Co-investigator	COVID-19 and Cancer Consortium
Justin F.	Gainor		MD	Massachusetts General Hospital Cancer Center	Boston, Massachusetts, USA	Co-investigator	COVID-19 and Cancer Consortium
Jeffrey	Peppercorn		MD, MPH	Massachusetts General Hospital Cancer Center	Boston, Massachusetts, USA	Co-investigator	COVID-19 and Cancer Consortium
Kerry L.	Reynolds		MD	Massachusetts General Hospital Cancer Center	Boston, Massachusetts, USA	Site PI	COVID-19 and Cancer Consortium
Leyre	Zubiri		MD, PhD	Massachusetts General Hospital Cancer Center	Boston, Massachusetts, USA	Co-investigator	COVID-19 and Cancer Consortium
Jian Li	Campian		MD, PhD	Mayo Cinic	Arizona, Minnesota, USA	Co-investigator	COVID-19 and Cancer Consortium
Katherine E.	Smith		MD	Mayo Cinic	Arizona, Minnesota, USA	Co-investigator	COVID-19 and Cancer Consortium
Tanios S.	Bekaii-Saab		MD, FACP	Mayo Clinic	Arizona, Minnesota, USA	Co-investigator	COVID-19 and Cancer Consortium
Aakash	Desai		MD, MPH	Mayo Clinic	Arizona, Minnesota, USA	Co-investigator	COVID-19 and Cancer Consortium

Supplemental Online Content: Nonauthor Collaborators
*First name, last name, and suffix (if applicable) are required and will appear in PubMed.

*First Name and Middle Initial(s)	*Last Name	*Suffix (eg, Jr, III)	Academic Degrees	Institution	Location (city, state/province, country)	Role or Contribution, eg, chair, principal investigator	Group (if more than 1 Group listed in the byline) and/or Subgroup (eg, Steering Committee)
Thorvardur R.	Halfdanarson		MD	Mayo Clinic	Arizona, Minnesota, USA	Site PI	COVID-19 and Cancer Consortium
Michael J.	Joyner		MD	Mayo Clinic	Arizona, Minnesota, USA	Co-investigator	COVID-19 and Cancer Consortium
Irbaz B.	Riaz		MD, MS	Mayo Clinic	Arizona, Minnesota, USA	Co-investigator	COVID-19 and Cancer Consortium
Jonathon W.	Senefeld		PhD	Mayo Clinic	Arizona, Minnesota, USA	Co-investigator	COVID-19 and Cancer Consortium
Colt	Williams		MD	Mayo Clinic	Arizona, Minnesota, USA	Co-investigator	COVID-19 and Cancer Consortium
Mark	Bonnen		MD	Mays Cancer Center at UT Health San Antonio MD Anderson Cancer Center	San Antonio, Texas, USA	Co-investigator	COVID-19 and Cancer Consortium
Daruka	Mahadevan		MD, PhD	Mays Cancer Center at UT Health San Antonio MD Anderson Cancer Center	San Antonio, Texas, USA	Co-investigator	COVID-19 and Cancer Consortium
Amelie G.	Ramirez		DrPH, MPH	Mays Cancer Center at UT Health San Antonio MD Anderson Cancer Center	San Antonio, Texas, USA	Co-investigator	COVID-19 and Cancer Consortium
Mary	Salazar		DNP, MSN, RN, ANP-BC	Mays Cancer Center at UT Health San Antonio MD Anderson Cancer Center	San Antonio, Texas, USA	Co-investigator	COVID-19 and Cancer Consortium
Chen-Pin	Wang		PhD	Mays Cancer Center at UT Health San Antonio MD Anderson Cancer Center	San Antonio, Texas, USA	Co-investigator	COVID-19 and Cancer Consortium
Ruben A.	Mesa		MD, FACP	Mays Cancer Center at UT Health San Antonio MD Anderson Cancer Center	Winston-Salem, North Carolina, USA	Site-PI	COVID-19 and Cancer Consortium
Nathaniel	Bouganim		MD,FRCP(C)	McGill University Health Centre	Montreal, Quebec, Canada	Site PI	COVID-19 and Cancer Consortium

Supplemental Online Content: Nonauthor Collaborators
*First name, last name, and suffix (if applicable) are required and will appear in PubMed.

*First Name and Middle Initial(s)	*Last Name	*Suffix (eg, Jr, III)	Academic Degrees	Institution	Location (city, state/province, country)	Role or Contribution, eg, chair, principal investigator	Group (if more than 1 Group listed in the byline) and/or Subgroup (eg, Steering Committee)
Arielle	Elkrief		MD,FRCP(C)	McGill University Health Centre	Montreal, Quebec, Canada	Co-investigator	COVID-19 and Cancer Consortium
Feras	Moria		MBBS	McGill University Health Centre	Montreal, Quebec, Canada	Co-investigator	COVID-19 and Cancer Consortium
Justin	Panasci		MD	McGill University Health Centre	Montreal, Quebec, Canada	Co-investigator	COVID-19 and Cancer Consortium
Jesse	Papenburg		MD, MSc	McGill University Health Centre	Montreal, Quebec, Canada	Co-investigator	COVID-19 and Cancer Consortium
Donald C.	Vinh		MD, FRCP(C)	McGill University Health Centre	Montreal, Quebec, Canada	Co-investigator	COVID-19 and Cancer Consortium
Rahul	Nanchal		MD	Medical College of Wisconsin	Milwaukee, Wisconsin, USA	Site PI	COVID-19 and Cancer Consortium
Harpreet	Singh		MD	Medical College of Wisconsin	Milwaukee, Wisconsin, USA	Site PI	COVID-19 and Cancer Consortium
Nadia	Bahadur		MSCR	Memorial Sloan Kettering Cancer Center	New York, New York, USA	Co-investigator	COVID-19 and Cancer Consortium
Ting	Bao		MD, DABMA, MS	Memorial Sloan Kettering Cancer Center	New York, New York, USA	Co-investigator	COVID-19 and Cancer Consortium
Puja H.	Nambiar		MD, MPH	Memorial Sloan Kettering Cancer Center	New York, New York, USA	Co-investigator	COVID-19 and Cancer Consortium
Roisin E.	O'Cearbhaill		MD	Memorial Sloan Kettering Cancer Center	New York, New York, USA	Co-investigator	COVID-19 and Cancer Consortium
Esperanza B.	Papadopoulos		MD	Memorial Sloan Kettering Cancer Center	New York, New York, USA	Co-investigator	COVID-19 and Cancer Consortium
John	Philip		MS	Memorial Sloan Kettering Cancer Center	New York, New York, USA	Co-investigator	COVID-19 and Cancer Consortium
Gregory J.	Riely		MD, PhD	Memorial Sloan Kettering Cancer Center	New York, New York, USA	Site PI	COVID-19 and Cancer Consortium
Mark	Robson		MD	Memorial Sloan Kettering Cancer Center	New York, New York, USA	Co-investigator	COVID-19 and Cancer Consortium

*First name, last name, and suffix (if applicable) are required and will appear in PubMed.

*First Name and Middle Initial(s)	*Last Name	*Suffix (eg, Jr, III)	Academic Degrees	Institution	Location (city, state/province, country)	Role or Contribution, eg, chair, principal investigator	Group (if more than 1 Group listed in the byline) and/or Subgroup (eg, Steering Committee)
Jonathan E.	Rosenberg		MD	Memorial Sloan Kettering Cancer Center	New York, New York, USA	Co-investigator	COVID-19 and Cancer Consortium
Adam J.	Schoenfeld		MD	Memorial Sloan Kettering Cancer Center	New York, New York, USA	Co-investigator	COVID-19 and Cancer Consortium
Rulla	Tamimi		MS	Meyer Cancer Center at Weill Cornell Medicine	New York, New York, USA	Site PI	COVID-19 and Cancer Consortium
Kim	Cerrone		RN	Missouri Baptist Medical Center	St. Louis, Missouri, USA	Co-investigator	COVID-19 and Cancer Consortium
Jennifer	Dill		BS, CCRP	Missouri Baptist Medical Center	St. Louis, Missouri, USA	Co-investigator	COVID-19 and Cancer Consortium
Bryan A.	Faller		MD	Missouri Baptist Medical Center	St. Louis, Missouri, USA	Site PI	COVID-19 and Cancer Consortium
Archana	Ajmera		MSN, ANP-BC, AOCNP	Moores Comprehensive Cancer Center at the University of California, San Diego	La Jolla, California, USA	Co-investigator	COVID-19 and Cancer Consortium
Sharon S.	Brouha		MD, MPH	Moores Comprehensive Cancer Center at the University of California, San Diego	La Jolla, California, USA	Co-investigator	COVID-19 and Cancer Consortium
Sharon	Choi		MD, PhD	Moores Comprehensive Cancer Center at the University of California, San Diego	La Jolla, California, USA	Co-investigator	COVID-19 and Cancer Consortium
Albert	Hsiao		MD, PhD	Moores Comprehensive Cancer Center at the University of California, San Diego	La Jolla, California, USA	Co-investigator	COVID-19 and Cancer Consortium
Erin G.	Reid		MD	Moores Comprehensive Cancer Center at the University of California, San Diego	La Jolla, California, USA	Co-investigator	COVID-19 and Cancer Consortium
Padmanabh S.	Bhatt		MD	Mount Auburn Hospital	Cambridge, Massachusetts, USA	Co-investigator	COVID-19 and Cancer Consortium
Melissa G.	Mariano		DO	Mount Auburn Hospital	Cambridge, Massachusetts, USA	Co-investigator	COVID-19 and Cancer Consortium

Supplemental Online Content: Nonauthor Collaborators
*First name, last name, and suffix (if applicable) are required and will appear in PubMed.

*First Name and Middle Initial(s)	*Last Name	*Suffix (eg, Jr, III)	Academic Degrees	Institution	Location (city, state/province, country)	Role or Contribution, eg, chair, principal investigator	Group (if more than 1 Group listed in the byline) and/or Subgroup (eg, Steering Committee)
Carey C.	Thomson		MD, FCCP, MPH	Mount Auburn Hospital	Cambridge, Massachusetts, USA	Co-investigator	COVID-19 and Cancer Consortium
Mary (Grace)	Glace		RN	Mount Carmel Health System	Columbus, Ohio, USA	Co-investigator	COVID-19 and Cancer Consortium
Jeanna L.	Knoble		MD	Mount Carmel Health System	Columbus, Ohio, USA	Site PI	COVID-19 and Cancer Consortium
Cameron	Rink		PhD, MBA	Mount Carmel Health System	Columbus, Ohio, USA	Co-investigator	COVID-19 and Cancer Consortium
Rosemary	Zacks		RN	Mount Carmel Health System	Columbus, Ohio, USA	Co-investigator	COVID-19 and Cancer Consortium
Sibel H.	Blau		MD	Northwest Medical Specialties	Tacoma, Washington, USA	Site PI	COVID-19 and Cancer Consortium
CarrieAnn	Brown		RN,OCN,CCRC	Northwest Medical Specialties	Tacoma, Washington, USA	Co-investigator	COVID-19 and Cancer Consortium
Swathi	Namburi		MD	Northwest Medical Specialties	Tacoma, Washington, USA	Co-investigator	COVID-19 and Cancer Consortium
Lauren D.	Pomerantz		BS	Penn State Health/Penn State Cancer Institute/St. Joseph Cancer Center	Reading, Pennsylvania, USA	Co-investigator	COVID-19 and Cancer Consortium
Marc A.	Rovito		MD, FACP	Penn State Health/Penn State Cancer Institute/St. Joseph Cancer Center	Reading, Pennsylvania, USA	Co-investigator	COVID-19 and Cancer Consortium
Pragati E.	Advani		MD, MPH, DrPH	Roswell Park Comprehensive Cancer Center	Buffalo, New York, USA	Co-investigator	COVID-19 and Cancer Consortium
Tara	Cronin			Roswell Park Comprehensive Cancer Center	Buffalo, New York, USA	Co-investigator	COVID-19 and Cancer Consortium
Bridget	Fleissner		BA	Roswell Park Comprehensive Cancer Center	Buffalo, New York, USA	Co-investigator	COVID-19 and Cancer Consortium
Shipra	Goel		MBBS, MD	Roswell Park Comprehensive Cancer Center	Buffalo, New York, USA	Co-investigator	COVID-19 and Cancer Consortium

*First name, last name, and suffix (if applicable) are required and will appear in PubMed.

*First Name and Middle Initial(s)	*Last Name	*Suffix (eg, Jr, III)	Academic Degrees	Institution	Location (city, state/province, country)	Role or Contribution, eg, chair, principal investigator	Group (if more than 1 Group listed in the byline) and/or Subgroup (eg, Steering Committee)
Dharmesh	Gopalakrishnan		MD	Roswell Park Comprehensive Cancer Center	Buffalo, New York, USA	Co-investigator	COVID-19 and Cancer Consortium
Francisco	Hernandez-Ilizaliturri		MD	Roswell Park Comprehensive Cancer Center	Buffalo, New York, USA	Co-investigator	COVID-19 and Cancer Consortium
Prantesh	Jain		MD, FACP	Roswell Park Comprehensive Cancer Center	Buffalo, New York, USA	Co-investigator	COVID-19 and Cancer Consortium
Ariffa	Kariapper		BDS, MSHS, CCRP	Roswell Park Comprehensive Cancer Center	Buffalo, New York, USA	Co-investigator	COVID-19 and Cancer Consortium
Ellis	Levine		MD	Roswell Park Comprehensive Cancer Center	Buffalo, New York, USA	Co-investigator	COVID-19 and Cancer Consortium
Melissa	Moffitt		MD	Roswell Park Comprehensive Cancer Center	Buffalo, New York, USA	Co-investigator	COVID-19 and Cancer Consortium
Tracey L.	O'Connor		MD	Roswell Park Comprehensive Cancer Center	Buffalo, New York, USA	Co-investigator	COVID-19 and Cancer Consortium
Igor	Puzanov		MD, MSCI, FACP	Roswell Park Comprehensive Cancer Center	Buffalo, New York, USA	Co-investigator	COVID-19 and Cancer Consortium
Laurie J.	Smith		MA	Roswell Park Comprehensive Cancer Center	Buffalo, New York, USA	Co-investigator	COVID-19 and Cancer Consortium
Camille P.	Wicher		Ph. D. Esq., RN, MSN	Roswell Park Comprehensive Cancer Center	Buffalo, New York, USA	Co-investigator	COVID-19 and Cancer Consortium
Emese	Zsiros		MD, PhD	Roswell Park Comprehensive Cancer Center	Buffalo, New York, USA	Co-investigator	COVID-19 and Cancer Consortium
Salma K.	Jabbour		MD	Rutgers Cancer Institute of New Jersey at Rutgers Biomedical and Health Sciences	New Brunswick, New Jersey, USA	Site PI	COVID-19 and Cancer Consortium
Christian F.	Misdary		MD	Rutgers Cancer Institute of New Jersey at Rutgers Biomedical and Health Sciences	New Brunswick, New Jersey, USA	Co-investigator	COVID-19 and Cancer Consortium

Supplemental Online Content: Nonauthor Collaborators
*First name, last name, and suffix (if applicable) are required and will appear in PubMed.

*First Name and Middle Initial(s)	*Last Name	*Suffix (eg, Jr, III)	Academic Degrees	Institution	Location (city, state/province, country)	Role or Contribution, eg, chair, principal investigator	Group (if more than 1 Group listed in the byline) and/or Subgroup (eg, Steering Committee)
Mansi R.	Shah		MD	Rutgers Cancer Institute of New Jersey at Rutgers Biomedical and Health Sciences	New Brunswick, New Jersey, USA	Co-investigator	COVID-19 and Cancer Consortium
Gerald	Batist		MD, FACP, FRCP (C)	Segal Cancer Centre, Jewish General Hospital, McGill University	Montreal, Quebec, Canada	Site PI	COVID-19 and Cancer Consortium
Erin	Cook		MSN	Segal Cancer Centre, Jewish General Hospital, McGill University	Montreal, Quebec, Canada	Co-investigator	COVID-19 and Cancer Consortium
Khashayar	Esfahani		MDCM, MSc	Segal Cancer Centre, Jewish General Hospital, McGill University	Montreal, Quebec, Canada	Co-investigator	COVID-19 and Cancer Consortium
Cristiano	Ferrario		MD	Segal Cancer Centre, Jewish General Hospital, McGill University	Montreal, Quebec, Canada	Co-investigator	COVID-19 and Cancer Consortium
Susie	Lau		MD, MSc, FRCSC	Segal Cancer Centre, Jewish General Hospital, McGill University	Montreal, Quebec, Canada	Co-investigator	COVID-19 and Cancer Consortium
Kim	Ma		MD, CM	Segal Cancer Centre, Jewish General Hospital, McGill University	Montreal, Quebec, Canada	Co-investigator	COVID-19 and Cancer Consortium
Wilson H.	Miller		MD, PhD	Segal Cancer Centre, Jewish General Hospital, McGill University	Montreal, Quebec, Canada	Co-investigator	COVID-19 and Cancer Consortium
Lawrence	Rudski		MD	Segal Cancer Centre, Jewish General Hospital, McGill University	Montreal, Quebec, Canada	Co-investigator	COVID-19 and Cancer Consortium
Miriam	Santos Dutra		MS, PhD	Segal Cancer Centre, Jewish General Hospital, McGill University	Montreal, Quebec, Canada	Co-investigator	COVID-19 and Cancer Consortium

*First name, last name, and suffix (if applicable) are required and will appear in PubMed.

*First Name and Middle Initial(s)	*Last Name	*Suffix (eg, Jr, III)	Academic Degrees	Institution	Location (city, state/province, country)	Role or Contribution, eg, chair, principal investigator	Group (if more than 1 Group listed in the byline) and/or Subgroup (eg, Steering Committee)
Machelle	Wilchesky		PhD	Segal Cancer Centre, Jewish General Hospital, McGill University	Montreal, Quebec, Canada	Co-investigator	COVID-19 and Cancer Consortium
Sana Z.	Mahmood		BA, BS	Sidney Kimmel Cancer Center at Thomas Jefferson University	Philadelphia, Pennsylvania, USA	Co-investigator	COVID-19 and Cancer Consortium
Christopher	McNair		PhD	Sidney Kimmel Cancer Center at Thomas Jefferson University	Philadelphia, Pennsylvania, USA	Co-investigator	COVID-19 and Cancer Consortium
Vasil	Mico		BS, MD candidate	Sidney Kimmel Cancer Center at Thomas Jefferson University	Philadelphia, Pennsylvania, USA	Co-investigator	COVID-19 and Cancer Consortium
Andrea Verghese	Rivera		MD	Sidney Kimmel Cancer Center at Thomas Jefferson University	Philadelphia, Pennsylvania, USA	Co-investigator	COVID-19 and Cancer Consortium
Becky	Dixon		MSN, BSN, RN	St. Elizabeth Healthcare	Edgewood, Kentucky, USA	Co-investigator	COVID-19 and Cancer Consortium
Hannah	Heilman		BS	St. Elizabeth Healthcare	Edgewood, Kentucky, USA	Co-investigator	COVID-19 and Cancer Consortium
Barbara B.	Logan		MS	St. Elizabeth Healthcare	Edgewood, Kentucky, USA	Co-investigator	COVID-19 and Cancer Consortium
Chaitanya	Mandapakala		MD	St. Elizabeth Healthcare	Edgewood, Kentucky, USA	Co-investigator	COVID-19 and Cancer Consortium
Elwyn C.	Cabebe		MD	Stanford Cancer Institute at Stanford University	Palo Alto, California, USA	Co-investigator	COVID-19 and Cancer Consortium
Michael J.	Glover		MD	Stanford Cancer Institute at Stanford University	Palo Alto, California, USA	Co-investigator	COVID-19 and Cancer Consortium
Alokkumar	Jha		PhD	Stanford Cancer Institute at Stanford University	Palo Alto, California, USA	Co-investigator	COVID-19 and Cancer Consortium
Ali Raza	Khaki		MD	Stanford Cancer Institute at Stanford University	Palo Alto, California, USA	Co-investigator	COVID-19 and Cancer Consortium
Seema	Nagpal		MD	Stanford Cancer Institute at Stanford University	Palo Alto, California, USA	Co-investigator	COVID-19 and Cancer Consortium
Lidia	Schapira		MD, FASCO	Stanford Cancer Institute at Stanford University	Palo Alto, California, USA	Co-investigator	COVID-19 and Cancer Consortium

Supplemental Online Content: Nonauthor Collaborators
*First name, last name, and suffix (if applicable) are required and will appear in PubMed.

*First Name and Middle Initial(s)	*Last Name	*Suffix (eg, Jr, III)	Academic Degrees	Institution	Location (city, state/province, country)	Role or Contribution, eg, chair, principal investigator	Group (if more than 1 Group listed in the byline) and/or Subgroup (eg, Steering Committee)
Sumit A.	Shah		MD, MPH	Stanford Cancer Institute at Stanford University	Palo Alto, California, USA	Site PI	COVID-19 and Cancer Consortium
Julie Tsu-Yu	Wu		MD, PhD	Stanford Cancer Institute at Stanford University	Palo Alto, California, USA	Co-investigator	COVID-19 and Cancer Consortium
Suki	Subbiah		MD	Stanley S. Scott Cancer Center at LSU Health Sciences Center	New Orleans, Louisiana, USA	Site PI	COVID-19 and Cancer Consortium
David	Whaley		JD	Stanley S. Scott Cancer Center at LSU Health Sciences Center	New Orleans, Louisiana, USA	Co-investigator	COVID-19 and Cancer Consortium
Gilberto de Lima	Lopes		MD, MBA, FAMS, FASCO	Sylvester Comprehensive Cancer Center at the University of Miami Miller School of Medicine	Miami, Florida, USA	Site PI; Steering Committee member	COVID-19 and Cancer Consortium
Karen	de Cardenas		RN, BSN	Tallahassee Memorial Healthcare	Tallahassee, Florida, USA	Co-investigator	COVID-19 and Cancer Consortium
Karen	Russell		MD, FACP	Tallahassee Memorial Healthcare	Tallahassee, Florida, USA	Site PI	COVID-19 and Cancer Consortium
Brittany	Stith		RN, BSN, OCN, CCRP	Tallahassee Memorial Healthcare	Tallahassee, Florida, USA	Co-investigator	COVID-19 and Cancer Consortium
Sharona	Taylor		BASc	Tallahassee Memorial Healthcare	Tallahassee, Florida, USA	Co-investigator	COVID-19 and Cancer Consortium
Justin F.	Klamerus		MD, MMM	The Barbara Ann Karmanos Cancer Institute at Wayne State University School of Medicine	Detroit, Michigan, USA	Co-investigator	COVID-19 and Cancer Consortium
Sanjay G.	Revankar		MD, FIDSA	The Barbara Ann Karmanos Cancer Institute at Wayne State University School of Medicine	Detroit, Michigan, USA	Site PI	COVID-19 and Cancer Consortium
Daniel	Addison		MD	The Ohio State University Comprehensive Cancer Center	Columbus, Ohio, USA	Co-investigator	COVID-19 and Cancer Consortium
James L.	Chen		MD	The Ohio State University Comprehensive Cancer Center	Columbus, Ohio, USA	Co-investigator	COVID-19 and Cancer Consortium

Supplemental Online Content: Nonauthor Collaborators
*First name, last name, and suffix (if applicable) are required and will appear in PubMed.

*First Name and Middle Initial(s)	*Last Name	*Suffix (eg, Jr, III)	Academic Degrees	Institution	Location (city, state/province, country)	Role or Contribution, eg, chair, principal investigator	Group (if more than 1 Group listed in the byline) and/or Subgroup (eg, Steering Committee)
Margaret E.	Gatti-Mays		MD	The Ohio State University Comprehensive Cancer Center	Columbus, Ohio, USA	Co-investigator	COVID-19 and Cancer Consortium
Sachin R.	Jhawar		MD	The Ohio State University Comprehensive Cancer Center	Columbus, Ohio, USA	Site PI	COVID-19 and Cancer Consortium
Joshua D.	Palmer		MD	The Ohio State University Comprehensive Cancer Center	Columbus, Ohio, USA	Co-investigator	COVID-19 and Cancer Consortium
Clement	Pillainayagam		MD	The Ohio State University Comprehensive Cancer Center	Columbus, Ohio, USA	Co-investigator	COVID-19 and Cancer Consortium
Daniel G.	Stover		MD	The Ohio State University Comprehensive Cancer Center	Columbus, Ohio, USA	Co-investigator	COVID-19 and Cancer Consortium
Sarah	Wall		MD	The Ohio State University Comprehensive Cancer Center	Columbus, Ohio, USA	Co-investigator	COVID-19 and Cancer Consortium
Nicole O.	Williams		MD	The Ohio State University Comprehensive Cancer Center	Columbus, Ohio, USA	Co-investigator	COVID-19 and Cancer Consortium
Saqib Hussen	Abbasi		MD	The University of Kansas Cancer Center	Kansas City, Kansas, USA	Co-investigator	COVID-19 and Cancer Consortium
Sandy	Annis		BA, CCRP	The University of Kansas Cancer Center	Kansas City, Kansas, USA	Co-investigator	COVID-19 and Cancer Consortium
Steven	Greenland		BA	The University of Kansas Cancer Center	Kansas City, Kansas, USA	Co-investigator	COVID-19 and Cancer Consortium
Regina	Jones			The University of Kansas Cancer Center	Kansas City, Kansas, USA	Co-investigator	COVID-19 and Cancer Consortium
Anup	Kasi		MD, MPH	The University of Kansas Cancer Center	Kansas City, Kansas, USA	Co-investigator	COVID-19 and Cancer Consortium
Crosby D.	Rock		MD	The University of Kansas Cancer Center	Kansas City, Kansas, USA	Co-investigator	COVID-19 and Cancer Consortium
Elizabeth M.	Wulff-Burchfield		MD	The University of Kansas Cancer Center	Kansas City, Kansas, USA	Site PI	COVID-19 and Cancer Consortium
Melissa	Smits		APC	ThedaCare Cancer Care	Appleton, Wisconsin, USA	Co-investigator	COVID-19 and Cancer Consortium

*First name, last name, and suffix (if applicable) are required and will appear in PubMed.

*First Name and Middle Initial(s)	*Last Name	*Suffix (eg, Jr, III)	Academic Degrees	Institution	Location (city, state/province, country)	Role or Contribution, eg, chair, principal investigator	Group (if more than 1 Group listed in the byline) and/or Subgroup (eg, Steering Committee)
Matthias	Weiss		MD, PhD	ThedaCare Cancer Care	Appleton, Wisconsin, USA	Site PI	COVID-19 and Cancer Consortium
David D.	Chism		MD	Thompson Cancer Survival Center	Knoxville, Tennessee, USA	Site PI	COVID-19 and Cancer Consortium
Susie	Owenby		RN, CCRP	Thompson Cancer Survival Center	Knoxville, Tennessee, USA	Co-investigator	COVID-19 and Cancer Consortium
Celina	Ang		MD	Tisch Cancer Institute at the Icahn School of Medicine at Mount Sinai	New York, New York, USA	Co-investigator	COVID-19 and Cancer Consortium
Matthew D.	Galsky		MD	Tisch Cancer Institute at the Icahn School of Medicine at Mount Sinai	New York, New York, USA	Co-investigator	COVID-19 and Cancer Consortium
Megan	Metzger			Tisch Cancer Institute at the Icahn School of Medicine at Mount Sinai	New York, New York, USA	Co-investigator	COVID-19 and Cancer Consortium
Jeffrey	Berenberg		MD, MACP	Tripler Army Medical Center	Honolulu, Hawaii, USA	Site PI	COVID-19 and Cancer Consortium
Catherine	Uyehara		PhD	Tripler Army Medical Center	Honolulu, Hawaii, USA	Co-investigator	COVID-19 and Cancer Consortium
Alyson	Fazio		APRN-BC	Tufts Medical Center Cancer Center	Boston, Massachusetts, USA	Site PI	COVID-19 and Cancer Consortium
Kathryn E.	Huber		MD	Tufts Medical Center Cancer Center	Boston, Massachusetts, USA	Co-investigator	COVID-19 and Cancer Consortium
Latoya N.	Lashley		MPH	Tufts Medical Center Cancer Center	Boston, Massachusetts, USA	Co-investigator	COVID-19 and Cancer Consortium
Mark H.	Sueyoshi		MD	Tufts Medical Center Cancer Center	Boston, Massachusetts, USA	Co-investigator	COVID-19 and Cancer Consortium
Kanishka G.	Patel		MD	UC Davis Comprehensive Cancer Center at the University of California at Davis	Davis, California, USA	Co-investigator	COVID-19 and Cancer Consortium
Jonathan W.	Riess		MD, MS	UC Davis Comprehensive Cancer Center at the University of California at Davis	Davis, California, USA	Site PI	COVID-19 and Cancer Consortium

Supplemental Online Content: Nonauthor Collaborators
*First name, last name, and suffix (if applicable) are required and will appear in PubMed.

*First Name and Middle Initial(s)	*Last Name	*Suffix (eg, Jr, III)	Academic Degrees	Institution	Location (city, state/province, country)	Role or Contribution, eg, chair, principal investigator	Group (if more than 1 Group listed in the byline) and/or Subgroup (eg, Steering Committee)
Hala T.	Borno		MD	UCSF Helen Diller Family Comprehensive Cancer Center at the University of California at San Francisco	San Francisco, California, USA	Co-investigator	COVID-19 and Cancer Consortium
Eric J.	Small		MD	UCSF Helen Diller Family Comprehensive Cancer Center at the University of California at San Francisco	San Francisco, California, USA	Co-investigator	COVID-19 and Cancer Consortium
Sylvia	Zhang		MS	UCSF Helen Diller Family Comprehensive Cancer Center at the University of California at San Francisco	San Francisco, California, USA	Co-investigator	COVID-19 and Cancer Consortium
Tessa M.	Andermann		MD, MPH	UNC Lineberger Comprehensive Cancer Center	Chapel Hill, North Carolina, USA	Co-investigator	COVID-19 and Cancer Consortium
Christopher E.	Jensen		MD	UNC Lineberger Comprehensive Cancer Center	Chapel Hill, North Carolina, USA	Co-investigator	COVID-19 and Cancer Consortium
Samuel M.	Rubinstein		MD	UNC Lineberger Comprehensive Cancer Center	Chapel Hill, North Carolina, USA	Site PI	COVID-19 and Cancer Consortium
William A.	Wood		MD, MPH	UNC Lineberger Comprehensive Cancer Center	Chapel Hill, North Carolina, USA	Site PI	COVID-19 and Cancer Consortium
Syed A.	Ahmad		MD, FACS	University of Cincinnati Cancer Center	Cincinnati, Ohio, USA	Co-investigator	COVID-19 and Cancer Consortium
Punita	Grover		MD	University of Cincinnati Cancer Center	Cincinnati, Ohio, USA	Co-investigator	COVID-19 and Cancer Consortium
Roman	Jandarov		PhD	University of Cincinnati Cancer Center	Cincinnati, Ohio, USA	Co-investigator	COVID-19 and Cancer Consortium
Jordan	Kharofa		MD	University of Cincinnati Cancer Center	Cincinnati, Ohio, USA	Co-investigator	COVID-19 and Cancer Consortium
Ningjing	Li		MD, PhD	University of Cincinnati Cancer Center	Cincinnati, Ohio, USA	Co-investigator	COVID-19 and Cancer Consortium

*First name, last name, and suffix (if applicable) are required and will appear in PubMed.

*First Name and Middle Initial(s)	*Last Name	*Suffix (eg, Jr, III)	Academic Degrees	Institution	Location (city, state/province, country)	Role or Contribution, eg, chair, principal investigator	Group (if more than 1 Group listed in the byline) and/or Subgroup (eg, Steering Committee)
Michelle	Marcum		MS	University of Cincinnati Cancer Center	Cincinnati, Ohio, USA	Co-investigator	COVID-19 and Cancer Consortium
Davendra P. S.	Sohal		MD, MPH	University of Cincinnati Cancer Center	Cincinnati, Ohio, USA	Co-investigator	COVID-19 and Cancer Consortium
Olga	Zamulko		MD	University of Cincinnati Cancer Center	Cincinnati, Ohio, USA	Co-investigator	COVID-19 and Cancer Consortium
Maheen	Abidi		MD	University of Colorado Cancer Center	Aurora, Colorado, USA	Co-investigator	COVID-19 and Cancer Consortium
Nicole B.	Balmaceda		MD	University of Colorado Cancer Center	Aurora, Colorado, USA	Co-investigator	COVID-19 and Cancer Consortium
Daniel W.	Bowles		MD	University of Colorado Cancer Center	Aurora, Colorado, USA	Site PI	COVID-19 and Cancer Consortium
Christopher L.	Geiger		MD	University of Colorado Cancer Center	Aurora, Colorado, USA	Co-investigator	COVID-19 and Cancer Consortium
Merry-Jennifer	Markham		MD, FACP, FASCO	University of Florida Health Cancer Center	Gainesville, Florida, USA	Site PI	COVID-19 and Cancer Consortium
Atlantis D.	Russ		MD, PhD	University of Florida Health Cancer Center	Gainesville, Florida, USA	Co-investigator	COVID-19 and Cancer Consortium
Haneen	Saker		MD	University of Florida Health Cancer Center	Gainesville, Florida, USA	Co-investigator	COVID-19 and Cancer Consortium
Jared D.	Acoba		MD	University of Hawaii Cancer Center	Honolulu, Hawaii, USA	Site PI	COVID-19 and Cancer Consortium
Horyun	Choi		MD	University of Hawaii Cancer Center	Honolulu, Hawaii, USA	Co-investigator	COVID-19 and Cancer Consortium
Young Soo	Rho		MD, CM	University of Hawaii Cancer Center	Honolulu, Hawaii, USA	Co-investigator	COVID-19 and Cancer Consortium
Lawrence E.	Feldman		MD	University of Illinois Hospital & Health Sciences System	Chicago, Illinois, USA	Site PI	COVID-19 and Cancer Consortium
Gerald	Gantt		MD	University of Illinois Hospital & Health Sciences System	Chicago, Illinois, USA	Co-investigator	COVID-19 and Cancer Consortium

Supplemental Online Content: Nonauthor Collaborators
*First name, last name, and suffix (if applicable) are required and will appear in PubMed.

*First Name and Middle Initial(s)	*Last Name	*Suffix (eg, Jr, III)	Academic Degrees	Institution	Location (city, state/province, country)	Role or Contribution, eg, chair, principal investigator	Group (if more than 1 Group listed in the byline) and/or Subgroup (eg, Steering Committee)
Kent F.	Hoskins		MD	University of Illinois Hospital & Health Sciences System	Chicago, Illinois, USA	Site PI	COVID-19 and Cancer Consortium
Mahir	Khan		MD	University of Illinois Hospital & Health Sciences System	Chicago, Illinois, USA	Co-investigator	COVID-19 and Cancer Consortium
Li C.	Liu		PhD	University of Illinois Hospital & Health Sciences System	Chicago, Illinois, USA	Co-investigator	COVID-19 and Cancer Consortium
Ryan H.	Nguyen		DO	University of Illinois Hospital & Health Sciences System	Chicago, Illinois, USA	Site PI	COVID-19 and Cancer Consortium
Mary M.	Pasquinelli		APRN, FNP-BC	University of Illinois Hospital & Health Sciences System	Chicago, Illinois, USA	Co-investigator	COVID-19 and Cancer Consortium
Candice	Schwartz		MD	University of Illinois Hospital & Health Sciences System	Chicago, Illinois, USA	Co-investigator	COVID-19 and Cancer Consortium
Neeta K.	Venepalli		MD, MBA	University of Illinois Hospital & Health Sciences System	Chicago, Illinois, USA	Co-investigator	COVID-19 and Cancer Consortium
Praveen	Vikas		MD, MBBS	University of Iowa Holden Comprehensive Cancer Center	Iowa City, Iowa, USA	Site PI	COVID-19 and Cancer Consortium
Yousef	Zakharia		MD	University of Iowa Holden Comprehensive Cancer Center	Iowa City, Iowa, USA	Co-investigator	COVID-19 and Cancer Consortium
Anne M.	Boldt		BA,MD	University of Michigan Rogel Cancer Center	Ann Arbor, Michigan, USA	Co-investigator	COVID-19 and Cancer Consortium
Leslie A.	Fecher		MD	University of Michigan Rogel Cancer Center	Ann Arbor, Michigan, USA	Site PI	COVID-19 and Cancer Consortium
Chris	Su		MD	University of Michigan Rogel Cancer Center	Ann Arbor, Michigan, USA	Co-investigator	COVID-19 and Cancer Consortium
Ragneel	Bijjula		MD	UPMC Western Maryland	Cumberland, Maryland, USA	Co-investigator	COVID-19 and Cancer Consortium
Blanche H.	Mavromatis		MD	UPMC Western Maryland	Cumberland, Maryland, USA	Site PI	COVID-19 and Cancer Consortium
Mildred E.	Seletyn		RN, BSN, OCN, CCRC	UPMC Western Maryland	Cumberland, Maryland, USA	Co-investigator	COVID-19 and Cancer Consortium

*First name, last name, and suffix (if applicable) are required and will appear in PubMed.

***First Name and Middle Initial(s)**	***Last Name**	***Suffix (eg, Jr, III)**	**Academic Degrees**	**Institution**	**Location (city, state/province, country)**	**Role or Contribution, eg, chair, principal investigator**	**Group (if more than 1 Group listed in the byline) and/or Subgroup (eg, Steering Committee)**
Barbara R.	Wood		RN, BSN,CCRC	UPMC Western Maryland	Cumberland, Maryland, USA	Co-investigator	COVID-19 and Cancer Consortium
Qamar U.	Zaman		MD	UPMC Western Maryland	Cumberland, Maryland, USA	Co-investigator	COVID-19 and Cancer Consortium
Virginia	Kaklamani		MD, DSc	UT Health San Antonio	Loma Linda, California, USA	Co-investigator	COVID-19 and Cancer Consortium
Alaina J.	Brown		MD, MPH	Vanderbilt-Ingram Cancer Center at Vanderbilt University Medical Center	Nashville, Tennessee, USA	Co-investigator	COVID-19 and Cancer Consortium
Lawrence J.	Charles		MD	Vanderbilt-Ingram Cancer Center at Vanderbilt University Medical Center	Nashville, Tennessee, USA	Co-investigator	COVID-19 and Cancer Consortium
Alex	Cheng		PhD	Vanderbilt-Ingram Cancer Center at Vanderbilt University Medical Center	Nashville, Tennessee, USA	Co-investigator	COVID-19 and Cancer Consortium
Marta A.	Crispens		MD, MBA	Vanderbilt-Ingram Cancer Center at Vanderbilt University Medical Center	Nashville, Tennessee, USA	Co-investigator	COVID-19 and Cancer Consortium
Sarah	Croessmann		PhD	Vanderbilt-Ingram Cancer Center at Vanderbilt University Medical Center	Nashville, Tennessee, USA	Co-investigator	COVID-19 and Cancer Consortium
Elizabeth J.	Davis		MD	Vanderbilt-Ingram Cancer Center at Vanderbilt University Medical Center	Nashville, Tennessee, USA	Co-investigator	COVID-19 and Cancer Consortium
Tan	Ding		MD	Vanderbilt-Ingram Cancer Center at Vanderbilt University Medical Center	Nashville, Tennessee, USA	Co-investigator	COVID-19 and Cancer Consortium
Stephany N.	Duda		PhD, MS	Vanderbilt-Ingram Cancer Center at Vanderbilt University Medical Center	Nashville, Tennessee, USA	Co-investigator	COVID-19 and Cancer Consortium

Supplemental Online Content: Nonauthor Collaborators
*First name, last name, and suffix (if applicable) are required and will appear in PubMed.

*First Name and Middle Initial(s)	*Last Name	*Suffix (eg, Jr, III)	Academic Degrees	Institution	Location (city, state/province, country)	Role or Contribution, eg, chair, principal investigator	Group (if more than 1 Group listed in the byline) and/or Subgroup (eg, Steering Committee)
Kyle T.	Enriquez		MSc, BS	Vanderbilt-Ingram Cancer Center at Vanderbilt University Medical Center	Nashville, Tennessee, USA	Co-investigator	COVID-19 and Cancer Consortium
Benjamin	French		PhD	Vanderbilt-Ingram Cancer Center at Vanderbilt University Medical Center	Nashville, Tennessee, USA	Co-investigator	COVID-19 and Cancer Consortium
Erin A.	Gillaspie		MD, MPH	Vanderbilt-Ingram Cancer Center at Vanderbilt University Medical Center	Nashville, Tennessee, USA	Co-investigator	COVID-19 and Cancer Consortium
Daniel J.	Hausrath		MD	Vanderbilt-Ingram Cancer Center at Vanderbilt University Medical Center	Nashville, Tennessee, USA	Co-investigator	COVID-19 and Cancer Consortium
Cassandra	Hennessy		MS	Vanderbilt-Ingram Cancer Center at Vanderbilt University Medical Center	Nashville, Tennessee, USA	Co-investigator	COVID-19 and Cancer Consortium
Douglas B.	Johnson		MD, MSCI	Vanderbilt-Ingram Cancer Center at Vanderbilt University Medical Center	Nashville, Tennessee, USA	Co-investigator	COVID-19 and Cancer Consortium
Judy T.	Lewis		MS, PhD	Vanderbilt-Ingram Cancer Center at Vanderbilt University Medical Center	Nashville, Tennessee, USA	Co-investigator	COVID-19 and Cancer Consortium
Xuanyi (Lexi)	Li		MD	Vanderbilt-Ingram Cancer Center at Vanderbilt University Medical Center	Nashville, Tennessee, USA	Co-investigator	COVID-19 and Cancer Consortium
Lauren S.	Prescott		MD, MPH	Vanderbilt-Ingram Cancer Center at Vanderbilt University Medical Center	Nashville, Tennessee, USA	Co-investigator	COVID-19 and Cancer Consortium
Sonya A.	Reid		MD, MPH	Vanderbilt-Ingram Cancer Center at Vanderbilt University Medical Center	Nashville, Tennessee, USA	Site PI; Steering Committee member	COVID-19 and Cancer Consortium

Supplemental Online Content: Nonauthor Collaborators
*First name, last name, and suffix (if applicable) are required and will appear in PubMed.

*First Name and Middle Initial(s)	*Last Name	*Suffix (eg, Jr, III)	Academic Degrees	Institution	Location (city, state/province, country)	Role or Contribution, eg, chair, principal investigator	Group (if more than 1 Group listed in the byline) and/or Subgroup (eg, Steering Committee)
Sara	Saif		BS	Vanderbilt-Ingram Cancer Center at Vanderbilt University Medical Center	Nashville, Tennessee, USA	Co-investigator	COVID-19 and Cancer Consortium
Yu	Shyr		PhD	Vanderbilt-Ingram Cancer Center at Vanderbilt University Medical Center	Nashville, Tennessee, USA	Co-investigator	COVID-19 and Cancer Consortium
David A.	Slosky		MD	Vanderbilt-Ingram Cancer Center at Vanderbilt University Medical Center	Nashville, Tennessee, USA	Co-investigator	COVID-19 and Cancer Consortium
Carmen C.	Solorzano		MD, FACS	Vanderbilt-Ingram Cancer Center at Vanderbilt University Medical Center	Nashville, Tennessee, USA	Co-investigator	COVID-19 and Cancer Consortium
Tianyi	Sun		MS	Vanderbilt-Ingram Cancer Center at Vanderbilt University Medical Center	Nashville, Tennessee, USA	Co-investigator	COVID-19 and Cancer Consortium
Lucy L.	Wang		BA	Vanderbilt-Ingram Cancer Center at Vanderbilt University Medical Center	Nashville, Tennessee, USA	Co-investigator	COVID-19 and Cancer Consortium
Yuanchu James	Yang		BS	Vanderbilt-Ingram Cancer Center at Vanderbilt University Medical Center	Nashville, Tennessee, USA	Co-investigator	COVID-19 and Cancer Consortium
David M.	Aboulafia		MD	Virginia Mason Cancer Institute	Seattle, Washington, USA	Site PI	COVID-19 and Cancer Consortium
Theresa M.	Carducci		MSN, RN, CCRP	Virtua Health	Marlton, New Jersey, USA	Co-investigator	COVID-19 and Cancer Consortium
Karen J.	Goldsmith		BSN, RN	Virtua Health	Marlton, New Jersey, USA	Co-investigator	COVID-19 and Cancer Consortium
Susan	Van Loon		RN, CTR, CCRP	Virtua Health	Marlton, New Jersey, USA	Co-investigator	COVID-19 and Cancer Consortium
Umit	Topaloglu		PhD, FAMIA	Wake Forest Baptist Comprehensive Cancer Center	Winston-Salem, North Carolina, USA	Site PI	COVID-19 and Cancer Consortium

Supplemental Online Content: Nonauthor Collaborators
*First name, last name, and suffix (if applicable) are required and will appear in PubMed.

*First Name and Middle Initial(s)	*Last Name	*Suffix (eg, Jr, III)	Academic Degrees	Institution	Location (city, state/province, country)	Role or Contribution, eg, chair, principal investigator	Group (if more than 1 Group listed in the byline) and/or Subgroup (eg, Steering Committee)
Joan	Moore		MSN, RN, OCN, CCRP	WellSpan Health	New York, Pennsylvania, USA	Site PI	COVID-19 and Cancer Consortium
Wilhelmina D.	Cabalona		MD	Wentworth-Douglass Hospital	Dover, New Hampshire, USA	Site PI	COVID-19 and Cancer Consortium
Elizabeth	Shisler		CRC	Wentworth-Douglass Hospital	Dover, New Hampshire, USA	Co-investigator	COVID-19 and Cancer Consortium
Briana	Barrow McCollough		BSc, CCRC	Willis-Knighton Cancer Center	Shreveport, Louisiana, USA	Co-investigator	COVID-19 and Cancer Consortium
Prakash	Peddi		MD	Willis-Knighton Cancer Center	Shreveport, Louisiana, USA	Site PI	COVID-19 and Cancer Consortium
Lane R.	Rosen		MD	Willis-Knighton Cancer Center	Shreveport, Louisiana, USA	Site PI	COVID-19 and Cancer Consortium
Mehmet A.	Bilen		MD	Winship Cancer Institute of Emory University	Atlanta, Georgia, USA	Site PI	COVID-19 and Cancer Consortium
Cecilia A.	Castellano		BA	Winship Cancer Institute of Emory University	Atlanta, Georgia, USA	Co-investigator	COVID-19 and Cancer Consortium
Deepak	Ravindranathan		MD, MS	Winship Cancer Institute of Emory University	Atlanta, Georgia, USA	Co-investigator	COVID-19 and Cancer Consortium
Navid	Hafez		MD, MPH	Yale Cancer Center at Yale University School of Medicine	New Haven, Connecticut, USA	Site PI	COVID-19 and Cancer Consortium
Roy S.	Herbst		MD, PhD	Yale Cancer Center at Yale University School of Medicine	New Haven, Connecticut, USA	Co-investigator	COVID-19 and Cancer Consortium
Patricia	LoRusso		DO, PhD	Yale Cancer Center at Yale University School of Medicine	New Haven, Connecticut, USA	Co-investigator	COVID-19 and Cancer Consortium
Maryam B.	Lustberg		MD, MPH	Yale Cancer Center at Yale University School of Medicine	New Haven, Connecticut, USA	Co-investigator	COVID-19 and Cancer Consortium
Tyler	Masters		MS	Yale Cancer Center at Yale University School of Medicine	New Haven, Connecticut, USA	Co-investigator	COVID-19 and Cancer Consortium
Catherine	Stratton		BA, MPH	Yale Cancer Center at Yale University School of Medicine	New Haven, Connecticut, USA	Co-investigator	COVID-19 and Cancer Consortium

Supplemental Online Content: Nonauthor Collaborators

*First name, last name, and suffix (if applicable) are required and will appear in PubMed.

*First Name and Middle Initial(s)	*Last Name	*Suffix (eg, Jr, III)	Academic Degrees	Institution	Location (city, state/province, country)	Role or Contribution, eg, chair, principal investigator	Group (if more than 1 Group listed in the byline) and/or Subgroup (eg, Steering Committee)
Corrie A.	Painter		PhD	Broad Institute of MIT and Harvard	Cambridge, MA, USA	Steering Committee member	COVID-19 and Cancer Consortium
Solange	Peters		MD, PhD	Centre Hospitalier Universitaire Vaudois	Lausanne, Switzerland	Steering Committee member	COVID-19 and Cancer Consortium
