# Supplementary material for: Systemic Anticancer Therapy and Thromboembolic Outcomes in Hospitalized Patients With Cancer and COVID-19
Source: JAMA Oncol. 2023 Aug 17;9(10):1390–400. doi: 10.1001/jamaoncol.2023.2934 (PMC10436185; doi:10.1001/jamaoncol.2023.2934)
Supplement: Supplement 3. — Data Sharing Statement [file jamaoncol-e232934-s003.pdf]

Data Sharing Statement

Gulati. Systemic Anticancer Therapy and Thromboembolic Outcomes in Hospitalized Patients With Cancer and COVID-19. *JAMA Oncol*. Published August 17, 2023. doi:10.1001/jamaoncol.2023.2934

Data
Data available: Yes
Data types: Data dictionary
How to access data: https://github.com/covidncancer/CCC19_dictionary
When available: With publication

Supporting Documents
Document types: Statistical/analytic code
How to access documents: https://github.com/covidncancer/CCC19_dictionary
When available: With publication

Additional Information
Who can access the data: anyone requesting the data
Types of analyses: for any purpose
Mechanisms of data availability: with investigator support
Any additional restrictions: NA
